# Close Contacts Unlocked: Nanopore-Stabilized Microvilli Bypass T Cell Receptor–Ligand-Dependent T Cell Activation

**DOI:** 10.1021/acsnano.5c16841

**Published:** 2026-07-22

**Authors:** Tamara Zünd, Sebastian Lickert, Willi Weber, Rafael Saxer, Marius Wenk, Janina Zünd, Tatiana Kovalchuk, Lucrezia Baldi, Nozie D. Aghaizu, Michael Daskalakis, Viola Vogel, Enrico Klotzsch

**Affiliations:** † Laboratory of Applied Mechanobiology, Department of Health Sciences and Technology, ETH Zürich, Gloriastrasse 37-39, 8092 Zurich, Switzerland; ‡ Institute for Biology, 9373 Humboldt Universität zu Berlin, 10099 Berlin, Germany; § Department of Hematology and Central Hematological Laboratory, Inselspital, Bern University Hospital, University of Bern, 3010 Bern, Switzerland; ∥ Berlin Institute of Health (BIH) at Charité−Universitätsmedizin Berlin, BIH Center for Regenerative Therapies (BCRT), Augustenburger Platz 1, 13353 Berlin, Germany; ⊥ Institute of Active Polymers, Helmholtz-Zentrum Hereon, 14513 Teltow, Germany

**Keywords:** nanopores, T cell signaling, mechanobiology, close contact, kinetic segregation, cell protrusions, nanoimmunotherapy

## Abstract

The biophysical microenvironment critically shapes T cell activation, yet how nanoscale geometry regulates signaling remains poorly understood. Here, we demonstrate that microvilli insertion into nanopores robustly activates primary human T cells in the absence of TCR ligands, in a pore-size-dependent manner. Nanopores of ∼240 nm in diameter elicit strong ERK phosphorylation, Ca^2+^ influx, and NFAT nuclear translocation, reaching levels comparable to biochemical stimulation using antibodies against the TCR complex and CD28. Although TCR knockdown attenuates responses, residual CD69 expression upon nanopore engagement indicates that nanoscale confinement lowers the activation threshold. Perturbation of membrane mechanics with GsMTx4 or methyl-β-cyclodextrin, as well as disruption of extracellular Ca^2+^-dependent interactions by EDTA, markedly impaired signaling, implicating extracellular calcium and membrane organization as key regulators of signaling. Together, these findings support a model in which ∼240 nm-sized nanopores promote stable close-contact patches that seed TCR signaling. Finally, we show that nanoporous stimulation combined with CD28 costimulation activates patient-derived T cells comparably to conventional methods, highlighting a strategy with translational potential for immunotherapy.

## Graphical abstract

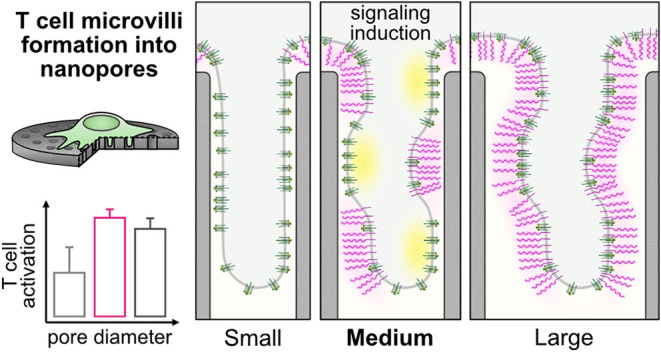

T cell activation is crucial for immune therapies,^1,2^ driving advancements in cancer treatment.^3^ Interdisciplinary approaches in immunology, material science, and nanotechnology have enabled the development of engineered substrates,^4^ nanoparticles,^5^ and three-dimensional (3D) matrices^6^ for better control and mimicry of *in vivo* T cell activation. These innovations enhance the T cell differentiation, function, and therapeutic effectiveness.^3^

T cell activation is typically initiated by binding of T cell receptors (TCRs) to specific peptide–MHC complexes on antigen-presenting cells (APCs).^7^ To scan for peptide-MHCs, T cells must overcome electrostatic and steric repulsion from the negatively charged glycocalyces of both T cells and APCs,^8,9^ largely imposed by bulky glycoproteins such as CD45 and CD43 that can extend up to 45 nm^10,11^ and prevent nonspecific activation.^8,9,12,13^ The T cell actin cytoskeleton helps overcome these barriers by probing and pressing against opposing surfaces, displacing the glycocalyx, and creating ∼15 nm close contacts that position TCRs near their ligands.^9,10,14–19^ Close contacts, edged by membrane undulations, lower TCR diffusion and exclude the bulky CD45 phosphatase, thereby shifting the local kinase-phosphatase balance and lowering the threshold for signaling.^20–24^ This kinetic segregation can seed low-level signaling even in the absence of a specific antigen.^23^ Ligand engagement further slows TCR diffusion,^7,20,23^ and allows forces to be applied onto the TCR complex,^25,26^ subsequently leading to initiation of signaling cascades.

Phosphorylation of ITAM motifs on the TCR-associated CD3 chains, driven by the kinase Lck,^27^ leads to the recruitment and activation of ZAP-70, which further recruits and phosphorylates adapter and scaffolding proteins, leading to the assembly of the signalosome.^27,28^ Together with costimulatory signals, particularly through CD28, key pathways, including MAPK, NFκB, and calcium signaling, are activated,^29^ leading to T cell activation indicated by CD69 expression, interleukine-2 (IL-2) secretion and proliferation.^30^

Within the T cell signaling cascades, PLCγ1 is a crucial effector protein.^31^ Upon recruitment to the signalosome, it cleaves phosphatidylinositol-4,5-bisphosphate (PIP_2_), thereby producing diacylglycerol (DAG) and inositol-1,4,5-trisphosphate (IP_3_). While DAG is important for inducing the MAPK pathway and activating the Protein Kinase C (PKC), leading to NFκB activity, IP_3_ triggers calcium release from the endoplasmic reticulum (ER).^28^ Depletion of calcium stores in the ER induces calcium influx into the cell through store-operated calcium entry (SOCE),^32^ driving nuclear translocation of NFAT.^33^

Beyond standard biochemical signaling, T cell function is influenced by various biophysical cues such as stiffness and viscoelasticity.^4,34–36^ Using their actin cytoskeleton, T cells generate forces to probe and engage their environment.^13,35,36^ We recently discovered that T cells sense nanopores through small membrane protrusions, called microvilli.^37^ Microvilli are highly dynamic structures that allow rapid scanning for antigens;^13,38^ moreover, they can function as signaling hubs.^39,40^ Surprisingly, microvilli formation into nanopores stabilized these protrusions (>10 min) and induced signaling without biochemical receptor stimulation using ligands.^37^ However, how microvilli confinement without biochemical stimulation triggers signaling remains unclear.

One potential mechanism is spatial confinement of microvilli by nanopores, which may stabilize spontaneously formed TCR clusters or, alternatively, exert lateral pressure on the T cell glycocalyx, thereby promoting the formation and stabilization of multiple close-contact patches along the microvillar shaft, lowering the threshold for initiating proximal TCR signaling.^12,41^ Furthermore, the stabilization of high membrane curvature could influence lipid and protein sorting and modulate protein conformation.^42–44^ Additionally, membrane strain could open mechanosensitive ion channels (MSC), triggering calcium influx.^45,46^ By stabilizing microvilli, nanopores could create a more controlled environment that enables signaling by giving more time for signaling events to occur, compared to the transient dynamics of unconfined microvilli.^37,38^

Close-contact zones have been described mainly as flat interfaces between opposing membranes, based largely on studies using supported lipid bilayers (SLBs) or glass substrates.^12,23^ More recent work, however, shows that these contacts are initiated at T cell microvilli tips before expanding and merging into larger planar signaling patches.^14,21,47^ Additional studies further indicate that the immunological synapse is not always a single flat structure but can also form as a multifocal synapse composed of multiple smaller close-contact zones across the T cell-APC interface.^38,48,49^ Whether such close contacts also form and persist in fully three-dimensional cell–cell interfaces–for example, along microvillar shafts rather than exclusively at their tips–remains unclear, in part due to limitations in temporal and 3D imaging resolution and the bias introduced by flat substrates in most experiments.^21,22^

Acknowledging the need for a more physiologically relevant 3D interface, we based our experimental approach on nanoporous surfaces previously shown to confine T cell microvilli.^37^ We first tested whether optimizing nanopore diameter could enhance the activation of primary human T cells and identified an optimal pore diameter that elicited robust activation without the need for TCR ligands. This mechano-stimulation, acting in a TCR complex-dependent but ligand-independent manner, triggered strong ERK phosphorylation and substantial Ca^2+^ influx, driving NFAT nuclear translocation to levels comparable to biochemical stimulation. In contrast, NFκB activation remained moderate unless CD28 costimulation was provided, indicating that nanopores do not simply mimic canonical TCR signaling and CD28 costimulation but instead preferentially amplify early TCR-linked MAPK and calcium/NFAT signaling. Perturbing membrane mechanics with GsMTx4 or methyl-β-cyclodextrin (MβCD) markedly reduced signaling, implicating membrane organization as an enabling factor. Together, these data support a model in which nanopores promote and stabilize close contacts that lower the threshold for TCR signaling.^50^ Finally, we show that this approach can activate patient-derived T cells, either alone or at levels comparable to conventional methods when combined with CD28 stimulation, underscoring potential applications in immunotherapy.

## Results

### Fine-Tuned Nanopore Diameter Enhances T Cell Activation

In a proof-of-concept, we previously discovered that T cells extend microvilli into nanopores (Figure 1a,b, and Supporting Movies 1 and 2) and show signs of activation without the need for anti-CD3 and anti-CD28 antibodies.^37^ However, the effect was modest and remained to be fully understood. To further explore this effect, we first aimed to maximize the pore-induced activation by using an optimized plasma treatment protocol for the nanoporous substrates and, moreover, by testing nanoporous anodic aluminum oxide (AAO) substrates with systematically varied pore diameters, which were analyzed using scanning electron microscopy (SEM) (Figure 1c,d, Supporting Figure 1a–c, and Table S1). After incubation of **naive primary human T cells** (CD4^+^ and CD8^+^) on these substrates for 24 h – without additional biochemical stimulation – activation was assessed by measuring CD69 with flow cytometry (Figure 1e,f). A clear dependence of T cell activation on pore diameter was observed. CD69 expression increased with pore size up to a maximum of ∼73% CD69^+^ T cells at ∼239 nm (**medium-sized** nanopores), after which activation declined with further increasing pore diameters (Supporting Figure 1d). IL-2 secretion on small (123 nm) and medium-sized nanopores additionally coated with anti-CD28 antibodies showed the same trend, with enhanced activation at medium pore diameters (Supporting Figure 1e). The activation peak at around 240 nm confirms a pore-diameter-dependent response in primary T cells, in line with Aramesh et al., who demonstrated a narrow activation window centered around ∼200 nm for Jurkat T cells, with diminished activation at both smaller and larger pore diameters. Notably, our platform achieved an approximately 6-fold higher activation rate than previously reported.^37^

**Figure 1:**
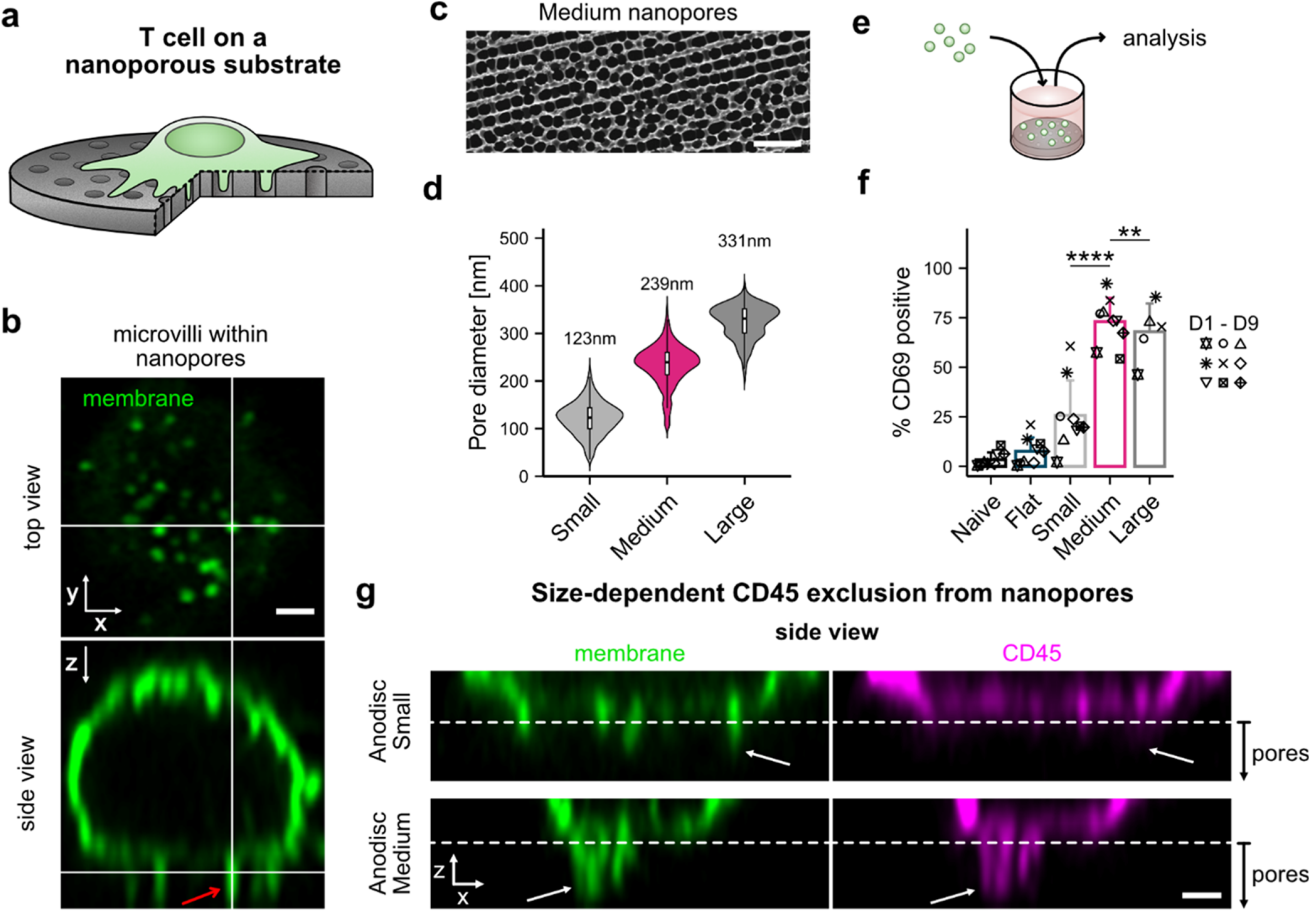
Fine-tuning T cell activation through nanopore diameter variation. (a) Schematic illustrating a T cell spreading on a nanoporous surface with microvilli extending into individual nanopores. (b) Orthogonal view of a naive primary human T cell on a nanoporous surface with the plasma membrane stained using Wheat Germ Agglutinin (WGA, green), imaged by Airyscan microscopy. The x-y view shows the first z-slice within the nanoporous surface, revealing microvilli cross sections as dot-like structures inside individual pores. The red arrow in the x-z view highlights a microvillus protruding into a nanopore. (c) SEM image of a nanoporous anodic aluminum oxide substrate with a 239 nm median pore diameter, referred to as a Medium nanoporous substrate. (d) Violin plot showing the distributions of nanopore diameters in anodic aluminum oxide substrates with varying pore sizes. The median pore diameter values are indicated above the violin plots. (e) Schematic illustrating the experimental setup for T cell activation using nanoporous substrates. Substrates are placed in a multiwell plate, and T cells are seeded on top of the substrates in T cell culture medium. (f) Activation induced in naive primary human T cells (CD8 + and CD4+), shown as the percentage of CD69 + T cells 24 h after seeding on the indicated substrates, measured by flow cytometry. Bars indicate the mean ± SD across donors (D; *n*_D_ = 4–9). Symbols represent mean values of technical replicates per donor. Statistical analysis was performed on per-donor mean values from donors present in both conditions using a paired *t* test with Benjamini–Hochberg (FDR) adjustment for multiple comparisons. Significance symbols represent adjusted *p*-values: ns: *p* ≥ 0.05, **: *p* < 0.01, and ****: *p* < 0.0001. (g) Cross section (x-z) of Airyscan microscopy images of representative T cells on Anodisc nanoporous substrates with small (107 nm) and medium-sized (229 nm) median nanopore diameters. CD45 (magenta) is visualized via antibody staining, and the membrane is stained using WGA (green). The dotted line marks the beginning of the nanopores, also marked by the arrow on the side. The white arrows indicate microvilli extending into nanopores. (b, c, g) Scale bar = 1 μm. Plots are isotropic in the x, y, and z directions.

### Pore-Size-Dependent CD45 Exclusion from Microvilli within Nanopores

In a previous study, Aramesh et al. proposed that CD45 is excluded from microvilli within nanopores due to size constraints, leading to spontaneous TCR signaling via uninhibited Lck.^37^ To re-evaluate this model with our newly defined optimal pore diameter, we examined CD45 localization on microvilli within medium-sized nanopores using structured illumination and Airyscan microscopy. (Supporting Information Figure 2a,b). Contrary to the previous study, we detected CD45 together with other key signaling molecules, including tyrosine phosphorylation (pY20), on these microvilli. Stimulated emission depletion (STED) microscopy confirmed CD45 within medium-sized nanopores but revealed a nonuniform distribution along the microvillar surface (Supporting Information Figure 2c).

The previous study, where CD45 pore-exclusion was observed,^37^ used Anodisc AAO substrates from a different supplier, stating 200 nm pores, however, consisting of distinct top- and backside morphologies. To address the discrepancy in our findings, we analyzed the pore diameter of both surfaces of these Anodisc substrates using SEM (Supporting Figure 3a,b) and examined CD45 localization using Airyscan microscopy. The backside of the Anodisc substrates featured pores comparable in size (∼229 nm) to our newly developed substrates and likewise showed CD45 within these pores (Figure 1g and Supporting Figure 3c). However, consistent with Aramesh et al., CD45 was absent from microvilli extending into smaller pores on the topside of the Anodisc substrates, measuring ∼107 nm in diameter. Activation achieved by these pore sizes was consistent with the results in Figure 1f (Supporting Figure 3d). The pore diameter-dependent exclusion of CD45 highlights how nanoscale topological confinement can determine whether proteins of a given size and structure access or are restricted from specific membrane regions. Our results suggest that exclusion of CD45 from nanopore-stabilized microvilli is not the primary driver of pore-induced activation, particularly at medium pore diameters. Instead, the presence of CD45 within nanopores may enhance signaling, consistent with studies showing that nanoscale segregation from the TCR, rather than complete spatial separation, is necessary for phosphorylation events to occur.^21,51^ Therefore, medium-sized nanopores may promote ligand-independent TCR activation by allowing CD45 entry while also permitting membrane undulations that support physiologically relevant protein sorting along the microvillar shaft.

### Nanopores Induce ERK Signaling and a Significant Calcium Influx Leading to NFAT Nuclear Translocation

To identify the still unknown initiator of nanopore-mediated T cell activation, we further examined the extent of MAPK and NFκB signaling in **naive primary human T cells**. We compared stimulation by medium-sized nanoporous substrates (hereafter termed ***Nanopores***) to biochemical activation using immobilized antibodies on flat aluminum surfaces. First, we assessed phosphorylation of ERK1/2 (pERK), a key kinase in the MAPK pathway typically activated downstream of TCR engagement. Using phospho-flow cytometry, we found that *Nanopores* induced strong ERK signaling over the course of 1 h, reaching ∼60% pERK^+^ T cells, comparable to full biochemical stimulation using both anti-CD3 and anti-CD28 antibodies (***Flat + aCD3/28***) (Figure 2a,b and Supporting Figure 4a). These results indicate that *Nanopores* robustly activate the MAPK pathway, demonstrating their capacity to initiate signaling independently of conventional ligand engagement.

**Figure 2:**
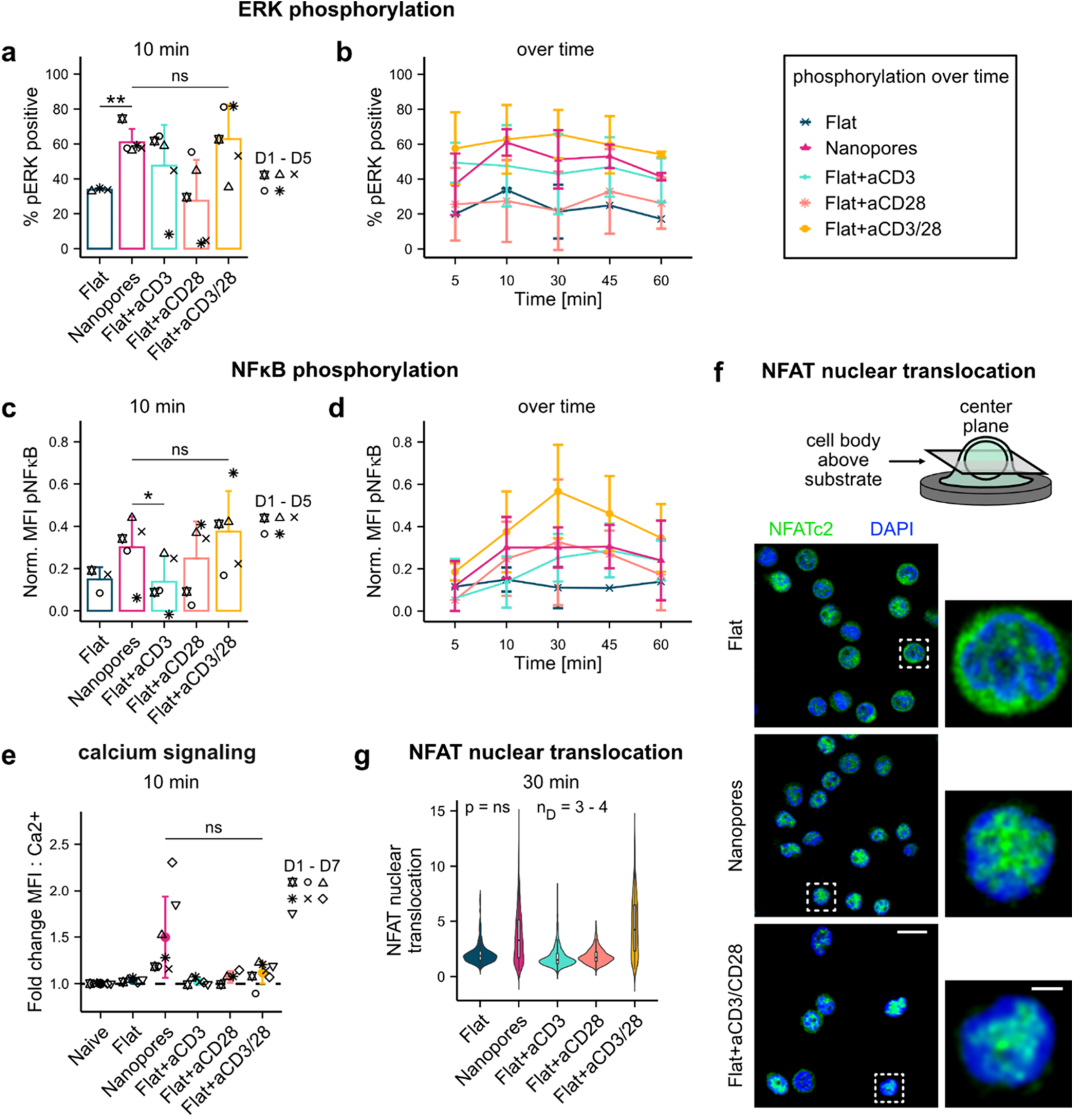
Major calcium and ERK signaling physically induced by ∼240 nm-sized nanopores. (a) Percentage of T cells containing phosphorylated ERK (pERK) after incubation on indicated substrates, measured by flow cytometry. (a) Percentage of pERK after 10 min of incubation. (b) Percentage of pERK over time. (c, d) Median fluorescence intensity (MFI) of phosphorylated NFκB (pNFκB) after incubation on the indicated substrates, measured by flow cytometry. Values are normalized per donor to the highest value over time after background subtraction (naive condition used as background). (c) pNFκB MFI after 10 min of incubation on the substrates. (d) MFI of pNFκB over time. (a, c) Bars indicate the mean ± SD across donors (D, *n*_D_ = 3–5). Symbols indicate mean values of technical replicates for each donor. (b, d) Legend is shown in the box. Points indicate the mean ± SD across donors (*n*_D_ = 3–5). (e) Fluo-8 MFI fold change relative to naive T cells as a calcium indicator after 10 min of incubation on different substrates, measured via flow cytometry. Points indicate the mean ± SD across donors (*n*_D_ = 4–7). Symbols indicate mean values of technical replicates for each donor. (f) Representative confocal microscopy composite images of primary human T cells incubated for 30 min on the respective substrates, stained for NFATc2 (green) and DAPI (blue). Scale bar = 10 μm. For each condition, a close-up view (dashed square) is shown. Scale bar = 2 μm. (g) Violin plots of the nuclear NFATc2 translocation ratio (mean nuclear intensity/mean cytoplasmic intensity), calculated from confocal microscopy images described in (f). Values of all individual T cells from all donors were pooled (*n*_D_ = 3 for Flat, Flat + aCD28, and Flat + aCD3/28; *n*_D_ = 4 for Flat + aCD3 and Nanopores). (a–g) Statistical analysis was performed on per-donor mean values from donors present in both conditions using a paired *t* test with Benjamini–Hochberg (FDR) adjustment for multiple comparisons. Significance symbols represent adjusted *p*-values: ns: *p* ≥ 0.05, *: *p* < 0.05, and **: *p* < 0.01.

Second, we assessed the phosphorylation of NFκB (pNFκB), a transcription factor typically strongly activated by CD28 costimulation, crucial for IL-2 secretion and T cell effector function.^30^ Surprisingly, *Nanopores* induced ∼2.2-fold higher pNFκB median fluorescent intensity (MFI) compared to anti-CD3-coated flat substrates (**Flat + aCD3** *)* after 10 min of activation, similar to stimulation with *Flat* + *aCD3/28* (Figure 2c). However, while signaling on *Flat* + *aCD3/28* increased further over time, pore-induced NFκB phosphorylation stayed constant (Figure 2d and Supporting Figure 4b), indicating only moderate activity of the costimulatory pathway. To address low NFκB activity, we tested *Nanopores* additionally coated with anti-CD28 antibodies (***Nanopores*** + ***aCD28***, Supporting Figure 4a–d). This resulted in a significant increase of pNFκB, even exceeding *Flat* + *CD3/28*, while ERK phosphorylation remained unaffected. Second, we assessed the phosphorylation of NFκB (pNFκB), a transcription factor typically strongly activated by CD28 costimulation, crucial for IL-2 secretion and T cell effector function.^30^ Surprisingly, *Nanopores* induced ∼2.2-fold higher pNFκB median fluorescent intensity (MFI) compared to anti-CD3-coated flat substrates (***Flat*** **+** **aCD3** *)* after 10 min of activation, similar to stimulation with *Flat* + *aCD3/28* (Figure 2c). However, while signaling on *Flat* + *aCD3/28* increased further over time, pore-induced NFκB phosphorylation stayed constant (Figure 2d and Supporting Figure 4b), indicating only moderate activity of the costimulatory pathway. To address low NFκB activity, we tested *Nanopores* additionally coated with anti-CD28 antibodies (***Nanopores + aCD28***, Supporting Figure 4a–d). This resulted in a significant increase of pNFκB, even exceeding *Flat* + *CD3/28*, while ERK phosphorylation remained unaffected.

Besides ERK and NFκB phosphorylation, calcium serves as a crucial secondary messenger in T cell activation. Calcium influx is typically triggered downstream of TCR engagement and stabilized by CD28 costimulation,^29^ leading to translocation of NFAT to the nucleus.^31^ To assess if *Nanopores* influence calcium signaling, we used Fluo-8 to measure intracellular calcium levels by flow cytometry 10 min after seeding (Figure 2e). Astonishingly, stimulation by *Nanopores* led to a 1.5-fold increase in Fluo-8 MFI compared to naive T cells, whereas full biochemical stimulation with *Flat* + *aCD3/28* only led to a 1.15-fold increase.

To validate the strong nanopore-induced Ca^2+^ response, we quantified the NFATc2 nuclear translocation by confocal fluorescence microscopy. Naive T cells were incubated for 30 min on the respective substrates and stained for NFATc2, DAPI, and F-actin, allowing sectioning into nuclear and cytosolic regions (Figure 2f), from which the nuclear/cytoplasmic ratio of the mean NFATc2 intensities was calculated (Figure 2g). On flat substrates, NFAT nuclear translocation required combined CD3 and CD28 stimulation (*flat* + *CD3/28*). Remarkably, T cells on *Nanopores* exhibited NFATc2 nuclear translocation approaching *Flat* + *CD3/28* levels, indicating robust activation of this pathway driven by strong Ca^2+^ influx. Additional coating of anti-CD28 (*Nanopores* + *aCD28*) showed only a small increase of calcium influx and no increase in NFAT nuclear translocation compared to sole stimulation by *Nanopores* (Supporting Information Figure 4e,f).

In summary, nanopore-stabilized microvilli induce strong MAPK and calcium/NFAT signaling, however, only moderate NFκB signaling, suggesting the involvement of the TCR but not the costimulatory molecule CD28 in the signal initiation.

### TCR Complex but Not CD28 Is Involved in Nanopore-Induced Activation

To investigate the broader role of the TCR complex in the nanopore-induced activation, we produced TCR knockdown (KD) T cells by electroporating preactivated **primary human T cells** with ribonucleoproteins (RNPs) consisting of Cas9 and an sgRNA targeting the T cell receptor α constant (TRAC) gene. As expected, restimulating TCR KD T cells on *Flat* + *aCD3* resulted in almost no increase of CD69 expression, comparable to resting T cells (Figure 3a). However, costimulation with CD3 and CD28 (*Flat* + *aCD3/28*) still resulted in 55% CD69^+^ T cells. Surprisingly, nanoporous stimulation of TCR KD T cells still led to ∼62% CD69^+^ T cells, only ∼20% reduction compared to TCR control T cells. However, when assessing the CD69 MFI, a clear decrease could be observed compared to the TCR control cells, similar to stimulation by *Flat* + *aCD3/28* (Figure[Fig fig3]b and Supporting Figure 5a). The decreased activation was also confirmed in Jurkat E6.1 TCR knockdown T cells (Supporting Figure 6a–c).

**Figure 3:**
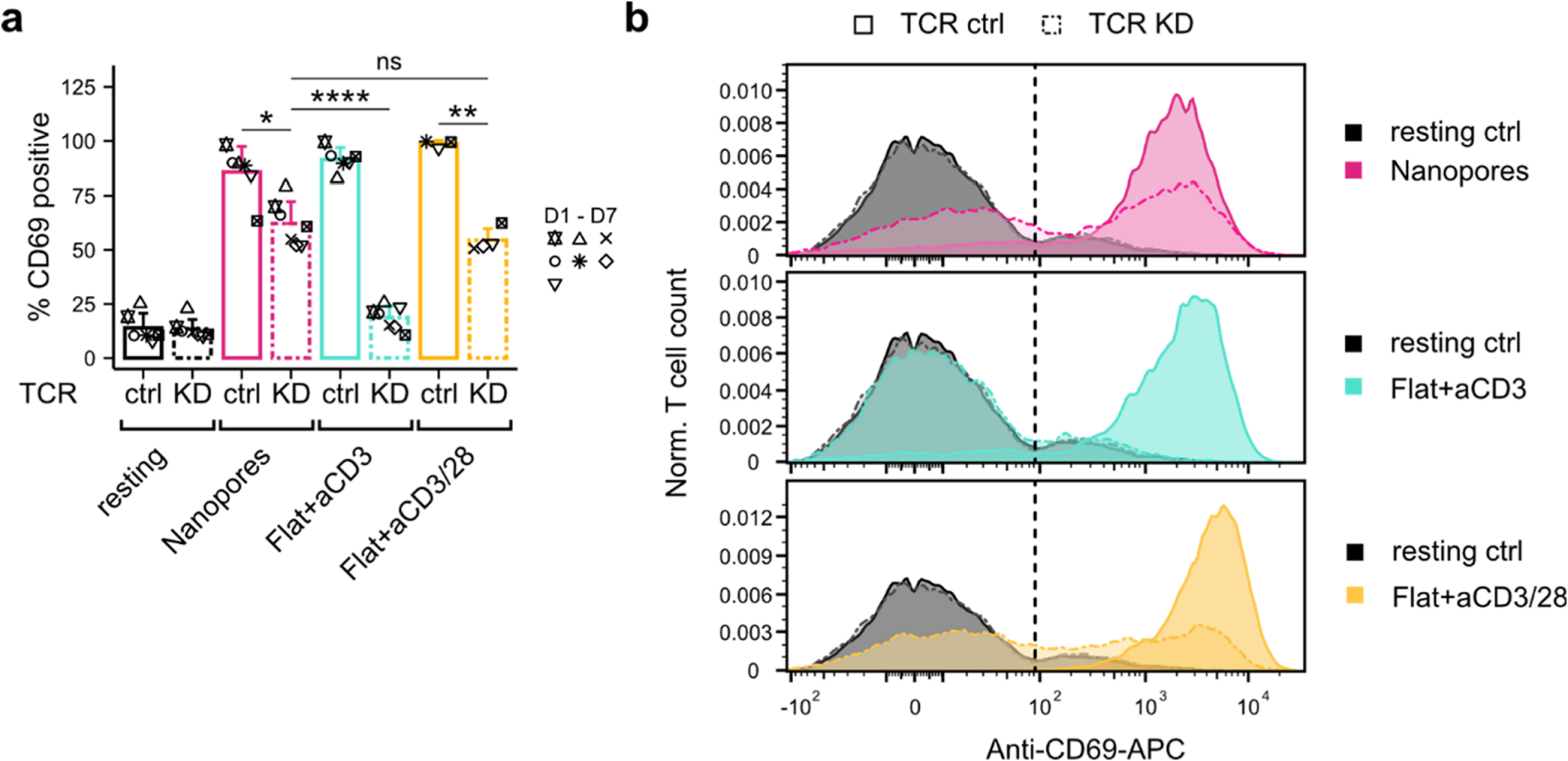
TCR knockdown T cells still express CD69 on nanoporous substrates. (a, b) CD69 expression was measured by flow cytometry in TCR knockdown (KD, CD3^–^ T cells) and untargeted TCR control (ctrl, CD3^+^ T cells) primary human T cells after 24 h of restimulation on the indicated substrates. Resting cells were not restimulated. (a) Percentage of CD69^+^ T cells. Bars indicate the mean ± SD across donors (D, *n*_D_ = 3–7). Symbols indicate mean values of technical replicates for each donor. Statistical analysis was performed on per-donor mean values from donors present in both conditions using a paired *t* test with Benjamini–Hochberg (FDR) adjustment for multiple comparisons. Significance symbols represent adjusted *p*-values: ns: *p* ≥ 0.05, *: *p* < 0.05, **: *p* <  0.01, and ****: *p* <  0.0001. (b) Exemplary histograms of CD69 expression pattern after stimulation by the indicated substrates with and without TCR expression. The dashed line indicates the gating threshold for CD69-positive T cells.

The reduced activation capacity confirms that TCR plays a central role in nanopore-induced signaling. However, the low-level activation still occurring in the absence of a fully assembled TCR complex suggests a synergistic contribution from other signaling components^52,53^ or, alternatively, that residual CD3 expression may be sufficient to initiate signaling. Furthermore, the threshold for signal induction might be generally lowered on nanopores by the stabilization of microvilli, facilitating signalosome formation.

To investigate the role of the costimulatory pathway in pore-induced activation, we knocked down CD28 in Jurkat E6.1 T cells with a second-generation lentiviral construct encoding Cas9 and a CD28-specific gRNA, followed by antibiotic selection and sorting for CD28-negative cells. Activation of CD28 KD cells on *Nanopores* or on *Flat* + *aCD3* showed no significant reduction of CD69 expression compared to the wild-type control (Supporting Information Figure 7a–c), indicating that CD28 is not involved in pore-induced activation.

Together, these results indicate that the TCR complex but not CD28 is involved in nanopore-induced signaling. They further suggest that residual CD3 expression or alternative signaling pathways are sufficient to drive a low-level CD69 expression.

### NFAT Signaling on *Nanopores* is Dependent on Store-Operated Calcium Entry

Given the markedly increased calcium influx and the downstream NFAT nuclear translocation observed on nanoporous substrates, we next sought to identify the signaling pathway responsible for this response. In conventional T cell signaling, the main calcium influx is mediated by SOCE through Stim1-Orai1 coupling after ER calcium-store depletion triggered by IP_3_ production by PLCγ1, typically induced by TCR triggering. However, mechanical stress such as membrane tension or shear forces can also cause calcium influx through MSCs, which are known to augment T cell activation.^45,46^ We therefore analyzed how inhibition of specific calcium-influx pathways affected early signaling events (Figure 4a) in naive primary human T cells stimulated for 30 min by Nanopores in comparison to *Flat* + *aCD3/28* as a positive control. pERK was measured with phospho-flow cytometry, whereas NFAT activity was measured by antibody staining of the transcription factors within isolated nuclei.

**Figure 4:**
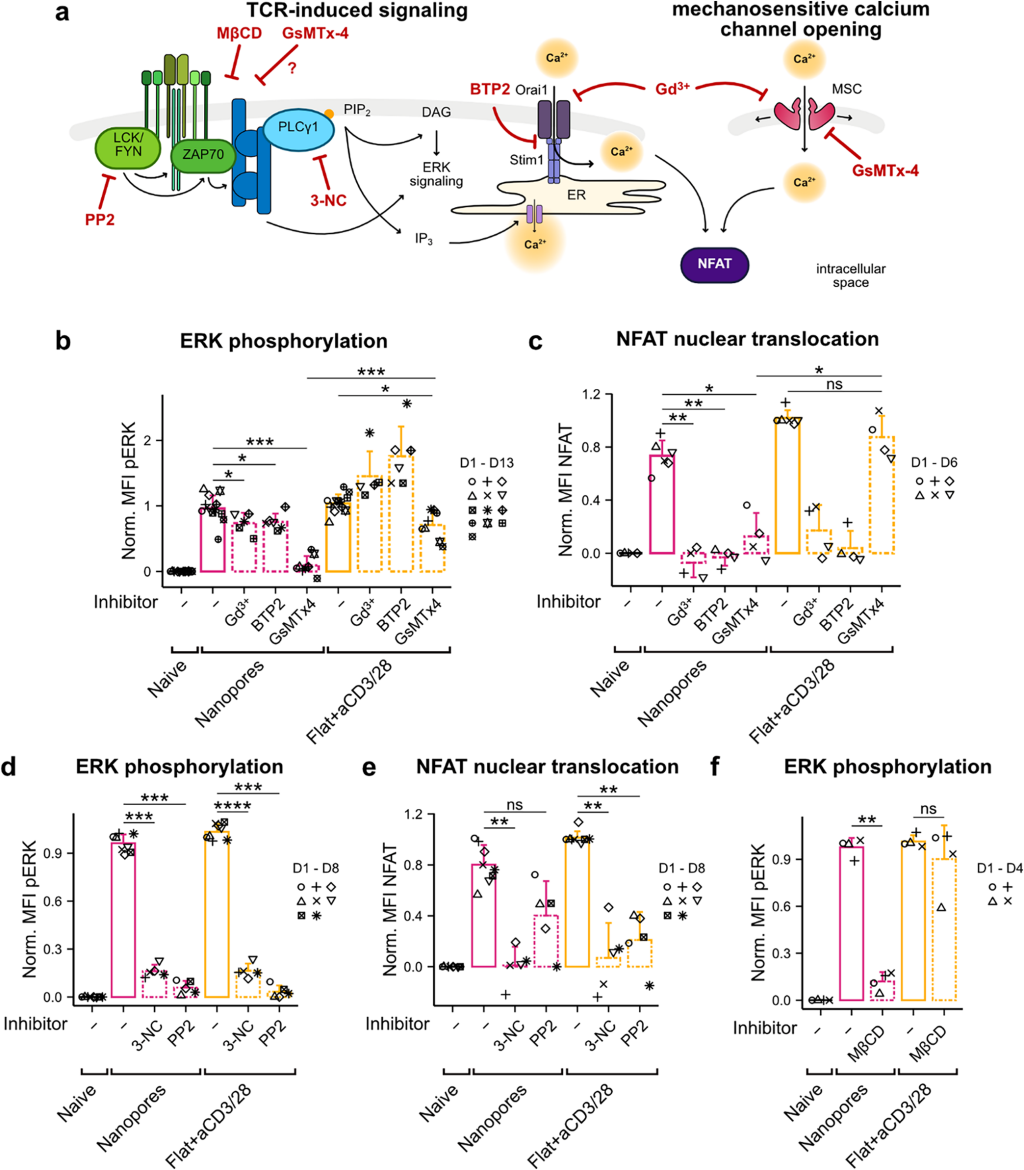
Nanopores induce ERK signaling and SOCE via the TCR pathway. (a) Schematic illustration of TCR-induced ERK and calcium signaling following ligand binding to the TCR. The diagram highlights TCR-mediated store-operated calcium entry (SOCE) in contrast to mechanosensitive calcium channels (MSC). It further highlights the sites of action of specific inhibitors. (b–f) Primary human T cells were preincubated with the indicated inhibitors for 30 min (Gd^3+^ 1 mM; BTP2 5 μM; GsMTx4 30 μM; 3-NC 10-μM; PP2 10 μM) or for 25 min (MβCD 8 mM). For T cells incubated with MβCD, the medium was replaced with fresh T cell medium prior to seeding; for all other inhibitors, the T cells were seeded in the presence of the inhibitor. The T cells were then incubated for 30 min on the indicated substrates. (b, d, f) Median fluorescent intensity (MFI) of phosphorylated ERK measured by flow cytometry, normalized per donor to all positive control samples (Flat + aCD3/28 and Nanopores) and naive as baseline. (c, e) NFAT (MFI) in isolated nuclei measured by flow cytometry, normalized per donor to the positive control sample Flat + aCD3/28 and naive as baseline. (b–f) Bars indicate the mean ± SD across donors (D, b: *n*_D_ = 5–13; c: *n*_D_ = 4–6; d: *n*_D_ = 5–8; e: *n*_D_ = 4–8; f: *n*_D_ = 4). Symbols indicate mean values of technical replicates for each donor. Statistical analysis was performed on per-donor mean values from donors present in both conditions using a paired *t* test with Benjamini–Hochberg (FDR) adjustment for multiple comparisons. Significance symbols represent adjusted *p*-values: ns: *p* ≥ 0.05, *: *p* < 0.05, **: *p* < 0.01, and ***: *p* < 0.001.

Blocking SOCE, specifically calcium influx through Stim1-Orai1 coupling, with BTP2 or Syntha-66, had, as expected, only mild inhibiting effects on ERK phosphorylation (Figure 4b and Supporting Figure 8a), while it drastically reduced nanopore-induced NFAT nuclear translocation (Figure 4c and Supporting Figure 8b). On *Flat* + *aCD3/28*, SOCE inhibition unexpectedly increased pERK levels despite the expected loss of NFAT signaling. The same results as SOCE blocking were achieved by treatment of the T cells with gadolinium (Gd^3+^), a general calcium channel blocker that inhibits both Orai1 and MSC.^54,55^ On *Nanopores*, the elevated calcium influx appears to enhance ERK signaling, likely through a positive feedback mechanism, whereas on *Flat* + *aCD3/28*, SOCE-mediated calcium entry may exert a negative regulatory effect on ERK phosphorylation,^56^ pointing to context-dependent feedback regulation of MAPK signaling.

Contrary to the results obtained with Gd^3+^, inhibition with the nonspecific MSC inhibitor GsMTx4 (30 μM) not only decreased NFAT nuclear translocation on *Nanopores* but also caused almost complete reduction of ERK phosphorylation–a pathway that is typically not induced by calcium entry. Signaling on *Flat* + *aCD3/28*, however, was mostly unaffected by GsMTx4.

While GsMTx4 is typically reported to have effects at concentrations ≤10 μM,^46^ strong inhibitory effects on nanopore-induced signaling only occurred at higher concentrations (Supporting Figure 9a,b). The concentration-dependent effect was also evident when assessing CD69 expression after 24 h of stimulation by *Nanopores*, showing complete suppression at 40 μM (Supporting Figure 9c–e), whereas 10 μM resulted only in ∼28% reduction of CD69^+^ T cells (Supporting Figure 9e,f). In contrast, antibody-induced CD69 expression was unaffected by GsMTx4. Live single-cell calcium imaging via Fluo-4 further revealed temporal differences of calcium influx on *Nanopores* in the presence of GsMTx4 (10 μM) (Supporting Figure 10a); however, the calcium peak did not fully return to baseline levels in the presence of the inhibitor (Supporting Figure 10a–c). This indicates that other pathways such as SOCE are contributing to the calcium influx. To further probe MSC involvement, we assessed calcium influx with Fluo-8 by flow cytometry in the presence of the Piezo1 activator, Yoda1. Yoda1 increased calcium influx on *Nanopores* to a degree comparable to *Flat* + *aCD3/28* (Supporting Figure 10d). This indicates that baseline Piezo1 activity is comparable between the two conditions and is unlikely to be fully engaged by *Nanopores*.

Even though the small peptide GsMTx4 inhibits cation-permeable MSC, its mechanism is bilayer-mediated rather than involving direct channel binding.^57^ At rest, the peptide associates superficially with the outer leaflet of the membrane, but under mechanical tension, it inserts more deeply into the bilayer,^58,59^ acting as a local tension buffer. At higher concentrations, GsMTx4 could crowd the outer leaflet and cause local thinning and altered lipid packing,^58,59^ potentially affecting the protein organization.

Due to the differences of GsMTx4 inhibition to the inhibition by Gd^3+^, the relatively high GsMTx4 concentrations required to suppress signaling, and the fact that GsMTx4 affects pathways that are not canonically downstream of MSCs, we suspect that the diminished signaling – especially ERK phosphorylation – arises from off-target effects related to bilayer perturbation, rather than from specific inhibition of MSC. To ensure that the reduced activation was not due to drug interference with microvilli formation, we confirmed microvilli presence within nanopores using Airyscan microscopy (Supporting Figure 11a–c).

Together, the data indicate that MSCs are not the primary source of calcium influx in nanopore-induced T cell activation. Instead, calcium signaling and NFAT nuclear translocation on nanopores are predominantly driven by the SOCE.

### NFAT and ERK Signaling Occur in a TCR-Dependent Mechanism

Given that GsMTx4 likely perturbs the lipid bilayer and could interfere with membrane-associated processes such as TCR or signalosome clustering, we next examined whether nanopore-induced calcium and MAPK signaling indeed follow the canonical TCR-coupled pathway. Therefore, we inhibited phospholipases C, including PLCγ1, using 3-nitrocoumarin (3-NC), and Src-family kinases, including Lck and Fyn, using PP2 (Figure 4a). As expected, PLC inhibition strongly reduced ERK signaling (Figure 4d), likely due to diminished DAG generation^51^ and abolished NFAT signaling on both substrates (Figure 4e), consistent with the loss of IP_3_ production and impaired SOCE. On both substrates, ERK phosphorylation was strongly dependent on Src-family kinases. In contrast, nuclear NFAT was only reduced by ∼ 52% on *Nanopores* upon PP2 treatment, compared to 79% reduction observed on *Flat+aCD3/28* (Figure 4e and Supporting Figure 12a,b), indicating a lower dependency of nanopore-induced NFAT signaling on Src-family kinase activity compared to biochemical signaling.

To further assess NFAT signaling dependency on the TCR complex, we analyzed early signaling events in TCR knockdown T cells generated as described above. These data confirmed a ∼90% reduction of pERK MFI values on both substrates upon TCR knockdown (Supporting Figure 12c), consistent with PP2 treatment. NFAT signaling was also markedly reduced on both substrates upon knockdown (∼83% reduction on *Nanopores*, Supporting Figure 12d); however, consistent with the data above, nanopore-induced NFAT nuclear translocation seemed to be less dependent on Src-family kinase activity than biochemically stimulated cells. The residual NFAT and ERK signaling may account for the ability of these cells to still express CD69, although at significantly reduced levels (Figure 3a,b).

In summary, these findings show that the TCR complex is crucial for nanopore-induced signaling, even in the absence of TCR ligands, leading to MAPK signaling and SOCE-driven NFAT nuclear translocation.

### Clustering of Signaling Proteins is Essential for Nanopore-Induced Signaling

As we observed, signaling on *Nanopores* remained strongly dependent on the TCR complex, although only residual Src-family kinase activity appears to be sufficient to induce NFAT nuclear translocation, suggesting a major structural role of the TCR in initiating early signaling events on nanoporous substrates. Together with the observation that GsMTx4 suppresses MAPK signaling through perturbation of membrane properties rather than MSC inhibition, these findings point toward membrane organization as a key regulator of the TCR-dependent signaling cascades on *Nanopores*. We therefore assessed the effect of disrupting cholesterol-dependent membrane architecture with MβCD, which extracts cholesterol from the plasma membrane and reduces the ability to form signaling clusters.^60^ Indeed, cholesterol extraction significantly reduced ERK phosphorylation on *Nanopores*, while antibody-induced signaling was not affected (Figure 4f).

### Close Contacts in Medium-Sized Nanopores as a Mechanistic Proposition of Ligand-Free T Cell Activation

Overall, the data point toward a mechanism in which nanopores stabilize membrane regions that function as close-contact signaling patches. The close-contact model–originally proposed theoretically^10,17,18^ and later confirmed at cell–cell interfaces^15,16,19^ – has so far been studied mostly with T cells in contact with flat substrates.^12,14,21,22,24,47^ By pressing against an opposing surface, T cells can overcome the protective barrier formed by the glycocalyx, which includes CD45, and establish close contacts.^9,11,14^ The resulting membrane undulations create alternating regions of close contact, where CD45 is locally excluded, and adjacent CD45-rich areas.^12,21,23^ Within these close-contact patches, TCR diffusion is reduced, sufficient to trigger signaling even in the absence of ligands.^23^ A related observation has been made at microvillar tips, where CD45 exclusion occurred even without contact to another surface;^61,62^ however, these exclusion zones are rather small (50–150 nm)^21,61,62^ compared to the larger close-contact patches generated at cell–cell or cell–surface interfaces.^12,14,21,47^

Nanoporous confinement of microvilli substantially increases the available membrane area along the shaft and base of the microvilli (Figure 5a), increasing potential close-contact sites in a pore-size-dependent manner. Small pores allow microvilli penetration but exclude CD45, limiting signal induction.^21^ Medium-sized nanopores allow CD45 access while still stabilizing or compressing the microvilli and their glycocalyx, thereby maintaining CD45-depleted patches, where close-contact signaling can occur. Large pores, in contrast, may fail to provide sufficient stabilization or compression to displace the CD45.

**Figure 5:**
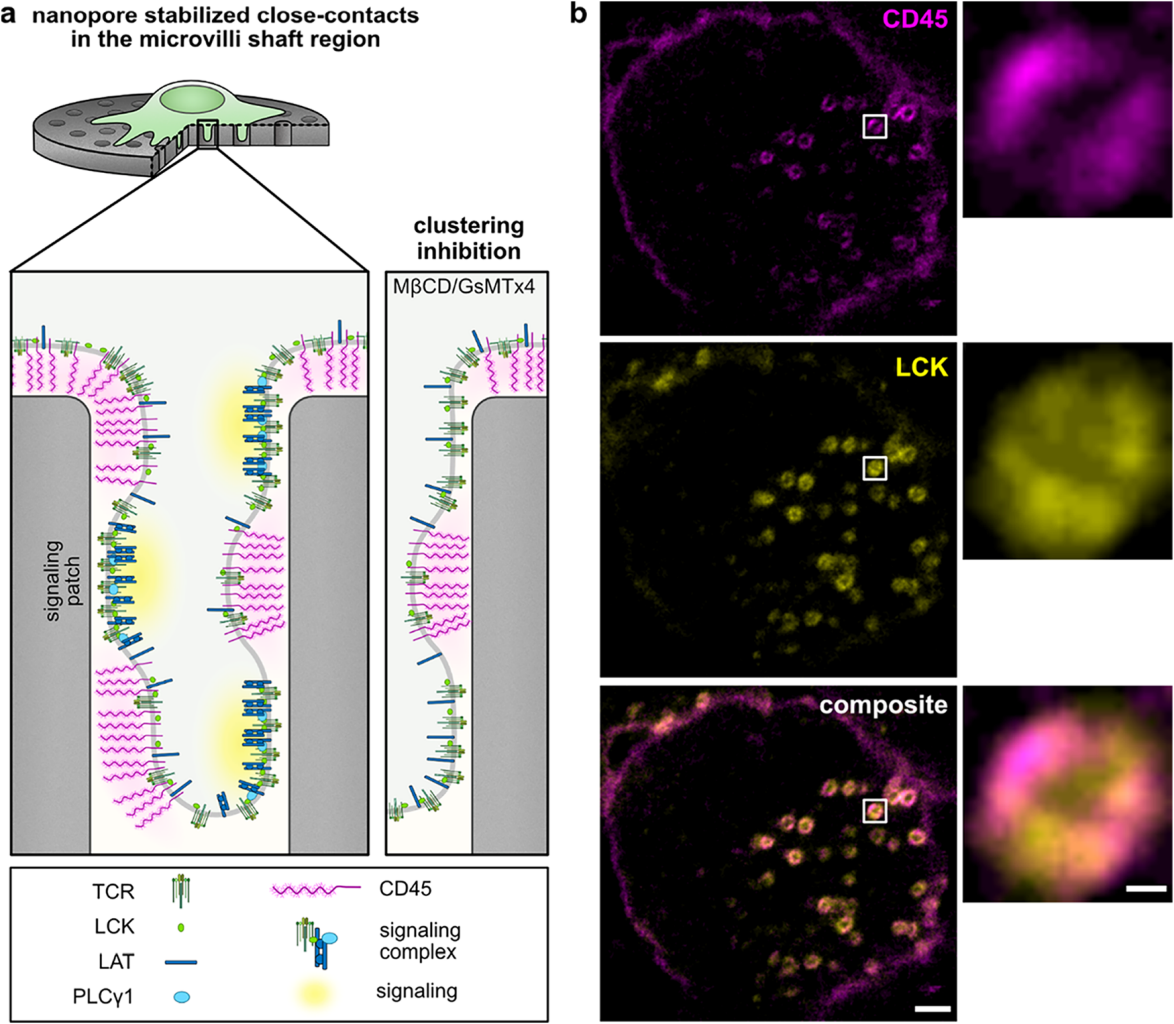
Close-contact patches as an activation hypothesis in nanopores. (a) Sketch illustrating the hypothesis where microvilli are stabilized within nanopores, stabilizing “close contact” patches where TCR signaling proteins cluster, while the bulky CD45 phosphatases are excluded. Influence of MβCD and GsMTx4 on clustering within close contact sites (right). (b) STED image of a T cell stained for CD45 (magenta) and LCK (yellow), acquired within nanopores, showing cross sections of microvilli, which appear as ring-like structures. Scale bar: 1 μm. A close-up view of a single microvillus (white square) is also shown on the side. Scale bar: 0.1 μm.

To assess this hypothesis, we imaged CD45 and Lck on microvilli extending into medium-sized nanopores using STED microscopy to visualize potential close-contact patches. The cross sections of the microvilli within nanopores appeared as ring-like structures (Figure 5b and Supporting Figure 13). CD45 displayed a patchy distribution with regions of markedly reduced signal, whereas Lck was more evenly, although not completely uniformly, distributed. As a result, some membrane regions contained both CD45 and Lck, while others were enriched for Lck but showed low CD45 intensity. These CD45-depleted, Lck-positive regions are consistent with the formation of close-contact patches that could support ligand-independent TCR triggering and signalosome assembly.

### Extracellular Calcium is Essential for Pore-Induced Signaling

Even though the signaling pattern induced by *Nanopores* can largely be explained by the formation of close contact patches, the unusually strong calcium influx remains striking. Moreover, MAPK signaling on *Nanopores* appears to be supported by calcium entry, whereas during antibody-induced activation, ERK phosphorylation is typically higher when the calcium influx is blocked. This raised the question of whether calcium plays a more central role in nanopore-induced signaling than in classical TCR stimulation.

Interestingly, when we chelated extracellular divalent cations using EDTA – or EGTA with added Mg^2+^ to isolate the specific contribution of Ca^2+^ – nanopore-induced ERK signaling was drastically reduced (Figure 6a), whereas on *Flat+aCD3/28*, only a minor decrease in pERK MFI was observed. While a reduction in nuclear NFAT is expected upon calcium chelation (Figure 6b), such a strong effect on ERK signaling is unusual. Remarkably, a stepwise increase in extracellular Ca^2+^ or Mg^2+^ produced a dose-dependent rise in pERK on *Nanopores*, with Ca^2+^ being more potent than Mg^2+^ (Figure 6c). In contrast, antibody-induced ERK signaling remained robust even in the absence of extracellular Ca^2+^ or Mg^2+^ (Supporting Figure 14a). This pronounced calcium dependency stands in contrast to the relatively stable ERK phosphorylation observed upon Gd^3+^ treatment (Figure 4b). Furthermore, intracellular calcium chelation with BAPTA-AM reduced ERK signaling and NFAT nuclear translocation on *Nanopores* in a manner comparable to antibody-induced activation (Supporting Figure 14b,c). The discrepancy between the strong reduction of ERK signaling upon extracellular calcium chelation and the minimal effect of Gd^3+^ indicates that calcium exerts a regulatory role from the extracellular side during nanopore-induced activation.

**Figure 6:**
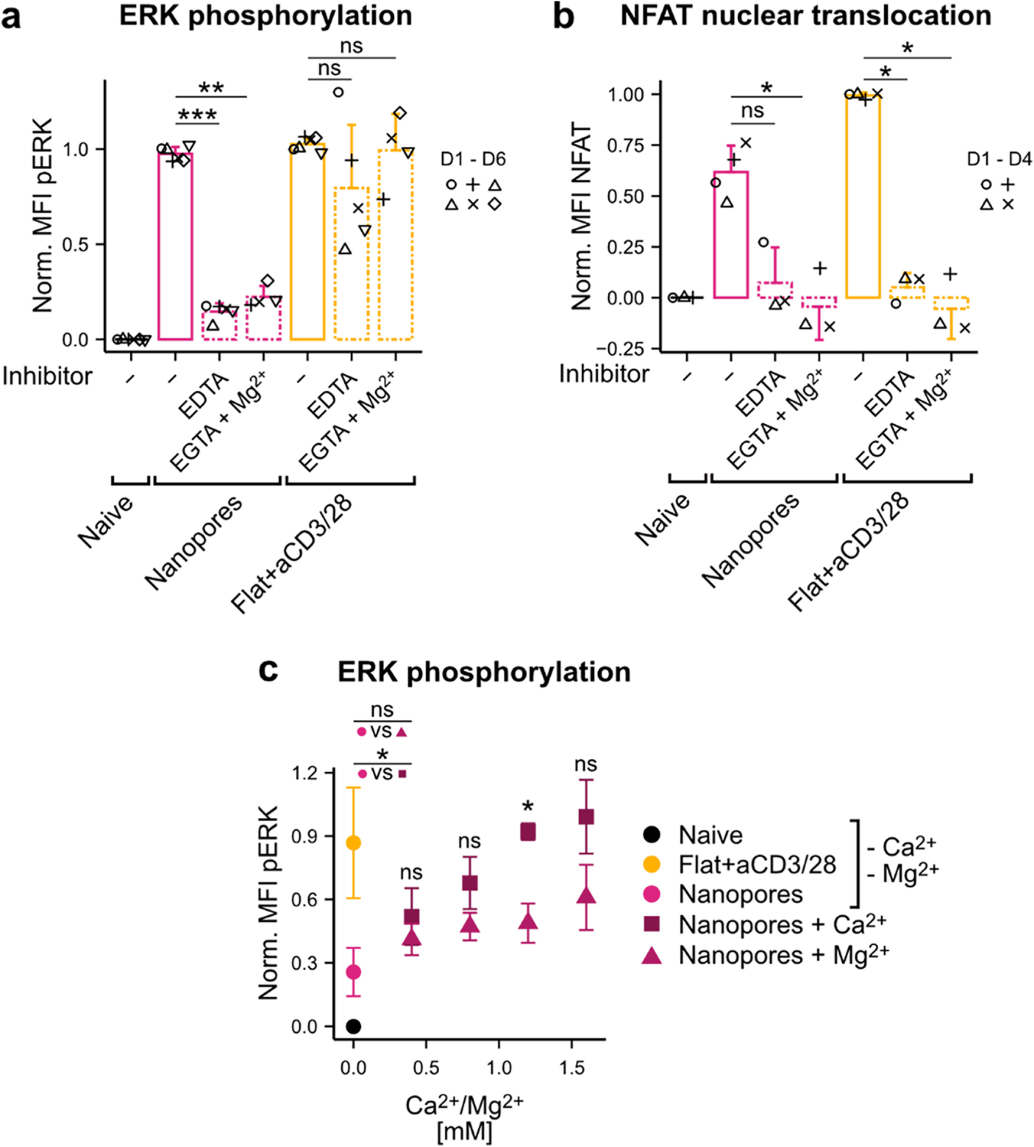
Extracellular calcium is essential for nanopore-induced signaling. (a–c) Primary human T cells were seeded and incubated for 30 min on the indicated substrates in (a, b) T cell medium containing EDTA (2 mM) or EGTA (1 mM) with additional magnesium (Mg2+, 0.6 mM) or in (c) HBSS medium containing the indicated concentration of calcium (Ca2+) or Mg2+. (a, c) Median fluorescent intensity (MFI) of phosphorylated ERK measured by flow cytometry, normalized per donor to all positive control samples. (a): Flat + aCD3/28 and nanopores; (c): flat+aCD3/28 ± 1.6 mM Ca2+ and nanopores (+1.6 mM Ca2+) and naive as baseline. (b) NFAT MFI in isolated nuclei measured by flow cytometry, normalized per donor to the positive control sample Flat+aCD3/28 and naive as baseline. (a, b) Bars indicate the mean ± SD across donors (D, (a): *n*_D_ = 3–4; (b): *n*_D_ = 4–6). Symbols indicate mean values of technical replicates for each donor. (c) Points indicate the mean ± SD across donors (nD = 3). (a–c) Statistical analysis was performed on per-donor mean values from donors present in both conditions using a paired *t* test with Benjamini–Hochberg (FDR) adjustment for multiple comparisons. Significance symbols represent adjusted *p*-values: ns: *p* ≥ 0.05, *: *p* < 0.05, **: *p* < 0.01, and ***: *p* < 0.001.

To exclude the possibility that calcium depletion impaired microvillus formation itself, we imaged cells under EDTA treatment using Airyscan and STED microscopy. EDTA did not suppress microvilli formation into nanopores (Supporting Information Figure 15a). Moreover, CD45 remained distributed in patches along the ring-like cross sections (Supporting Information Figure 15b), confirming that the structural basis for close-contact formation was preserved.

These findings indicate that calcium contributes an additional regulatory element to nanopore-induced signaling beyond its canonical role in NFAT activation, although the precise mechanism remains to be determined.

### Relevance for Clinical Application

To uncover potential applications of this activation method, we explored the extent of nanopore-induced activation. Remarkably, *Nanopores* induced CD69 expression in ∼73% of naive CD4^+^ and CD8^+^ T cells, comparable to *Flat* + *aCD3* (Supporting Figure 16a–c). CD28 coating on *Nanopores* further increased the CD69 MFI of CD69^+^ T cells. IL-2 secretion, which is typically only secreted upon TCR stimulation together with CD28, was roughly 10-fold higher on *Nanopores* than on *Flat* + *aCD3*, reaching ∼2.4 ng/mL (Supporting Figure 16d). Furthermore, nanoporous stimulation led to a strong but transient proliferative boost (Supporting Figure 16e), whereas stimulation with *Flat+aCD3* led to anergic T cells that declined after initial activation.

We furthermore tested *Nanopores* with **Pan T cells** from healthy donors and compared the activation to Pan T cells derived from diffuse large B-cell lymphoma (DLBCL) patients selected for CAR T cell therapy. The percentage of CD69^+^ cells was comparable between healthy and patient-derived T cells across all substrate conditions, including nanoporous substrates (Supporting Figure 16f). However, the CD69 MFI of CD69^+^ T cells was significantly reduced for patient-derived T cells, especially on *Nanopores+aCD28*, *Flat + aCD3/28*, and the clinical standard, *Dynabeads* (Figure 7a). When assessing IL-2 secretion, we observed an ∼11-fold increase of IL-2 secretion of healthy donor T cells when CD28 was coated on *Nanopores* compared to uncoated nanoporous substrates (Figure 7b). No significant differences were seen among *Nanopores+aCD28*, *Flat+aCD3/28*, and *Dynabeads*, nor between healthy and patient-derived cells.

**Figure 7:**
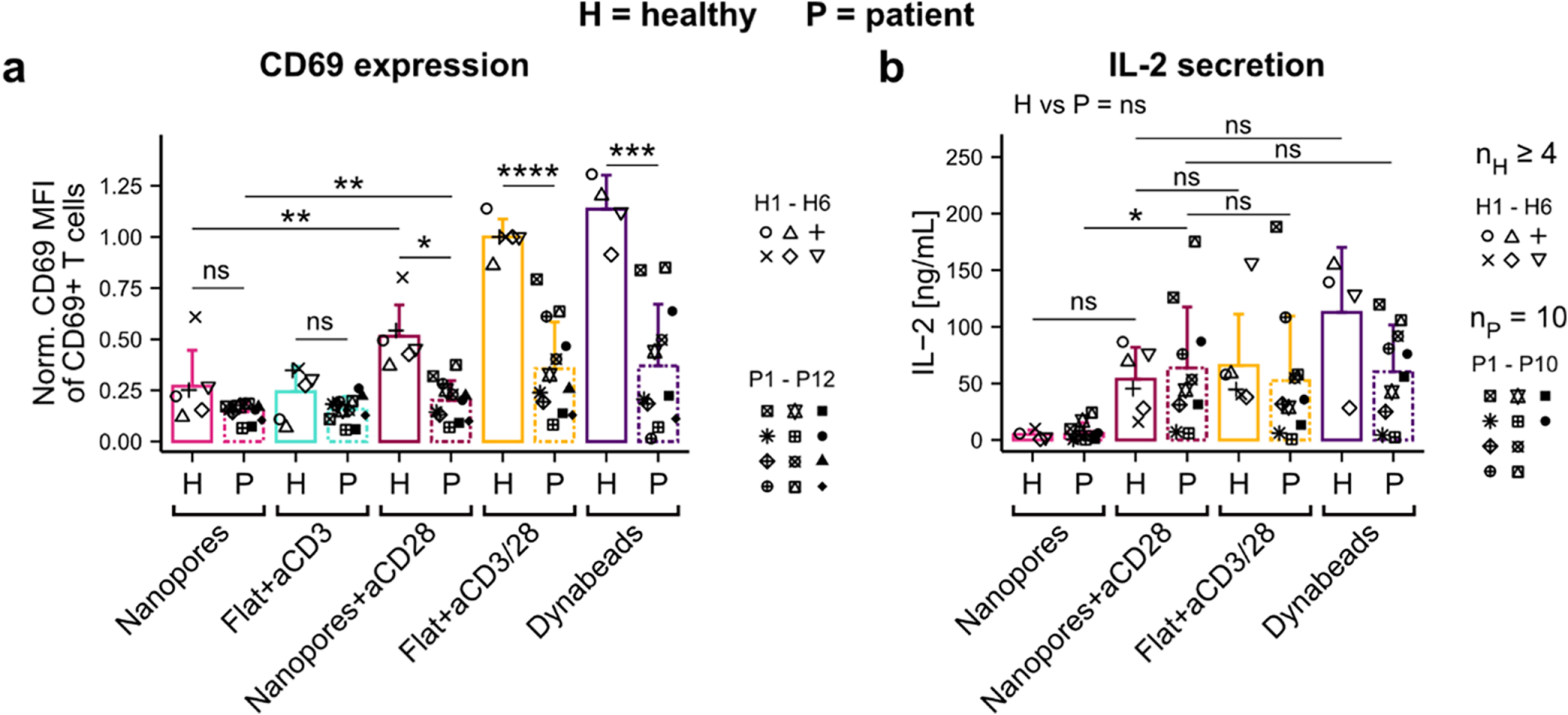
DLBCL Patient T cell activation by Nanopores. (a, b) Pan T cells from healthy and DLBCL patient donors were incubated on the indicated substrates for 24 h. (a) CD69 MFI of CD69^+^ T cells, normalized per experiment to the positive control samples (healthy Flat + aCD3/28) after baseline subtraction (healthy naive T cells used as baseline). (b) Absolute IL-2 secretion measured using a cytokine secretion assay. (a, b) Bars indicate the mean ± SD across healthy donors (D) or patients (P; a: *n*_D_ = 4–6, *n*_P_ = 11–12; b: *n*_D_ = 4–6, *n*_P_ = 10). Symbols indicate mean values of technical replicates for each healthy donor or patient. Statistical analyses within the patient and healthy donor groups were performed using paired *t* tests on per-donor mean values from donors present in both conditions. Comparisons between patient and healthy donor groups were performed using unpaired *t* tests. Multiple comparisons were corrected using the Benjamini–Hochberg false discovery rate (FDR) method. Significance symbols represent adjusted *p*-values: ns: *p* ≥ 0.05, *: *p* < 0.05, **: *p* < 0.01, ***: *p* < 0.001, and ****: *p* < 0.0001.

In summary, *Nanopores* alone elicit robust CD69 expression, and adding CD28 significantly boosts activation to levels comparable to those of the clinical standard *Dynabeads*.

## Discussion

Our findings revealed that microvilli formation into *Nanopores* activated naive primary human T cells without TCR ligands. *Nanopores* with ∼240 nm pore diameter elicited robust ERK phosphorylation and a significant calcium influx, driving NFAT nuclear translocation, leading to robust CD69 expression. However, costimulation using CD28 ligands was necessary to drive robust NFκB signaling and IL-2 secretion. Nanopore-induced early signaling was strongly dependent on the TCR complex, whereas activation was unaffected by CD28 knockdown. Thus, nanopores appear to provide a potent TCR-proximal biophysical stimulus that lowers the activation threshold. Furthermore, NFAT nuclear translocation showed strong dependency on SOCE, as shown by pharmacological inhibition of PLC and Stim1-Orai1. Further pharmacologic and ionic manipulations indicate that extracellular Ca^2+^, intact membrane mechanics, and cholesterol-dependent membrane and protein organization are prerequisites for nanopore-induced signaling. Together, these pore diameter-dependent findings support an activation mechanism involving close contacts along microvilli, stabilized by nanoporous confinement. Furthermore, activation by nanopores suggests a potentially more complex, 3D architecture of close-contact zones at cell–cell interfaces than what can be captured using planar substrates.^12,14,21–23,56^

To prevent spontaneous TCR activation, T cells employ tightly regulated mechanisms. The glycocalyx – a dense, glycoprotein-rich surface layer – must be displaced to allow membrane and, furthermore, receptor–ligand proximity.^9,10,47^ Additionally, the ITAM-containing cytoplasmic domains of CD3 subunits are sequestered within the membrane and require conformational changes to become accessible for phosphorylation.^63,64^ Furthermore, by dephosphorylating both inhibitory and activating phosphorylation sites on key residues of Lck, CD45 maintains Lck in a “primed” state, thereby preventing premature TCR signaling.^43,51^

Through cytoskeletal dynamics, T cells can apply mechanical forces to displace the glycocalyx, including CD45, and form close-contact patches,^9^ enabling TCR engagement with peptide-MHC complexes. When microvilli interact with nanoporous surfaces of optimal pore size, compression or shear forces could locally displace or condense the glycocalyx,^65^ causing localized membrane undulations and close contacts along the microvilli shaft that press against the nanopore walls. Nanopore geometry – specifically, the pore diameter – plays a critical role. Small pores exclude key proteins, such as CD45 due to its bulky extracellular domain,^37^ limiting signal induction,^21^ while large pores lack the compression needed to stabilize or even create close contacts, similar to unrestricted microvilli.^38^ Optimally sized nanopores, however, could stabilize numerous contact zones and extended microvilli dwell time within the nanopores, allowing sustained signaling. Within these close-contact zones, the local kinase-phosphatase balance is shifted by reduced lateral TCR diffusion^23^ and local CD45 segregation,^12,20–22^ which likely corresponds to the CD45-low regions observed in the microvillar cross sections in our STED images. Stabilization of these close-contact patches on microvilli could therefore sustain the kinetic segregation, enough to induce sustained ITAM phosphorylation even without extracellular ligand engagement,^23^ leading to downstream signaling.

While on *Nanopores*, NFAT nuclear translocation is TCR-dependent, it showed reduced sensitivity to Lck inhibition. The scaffold provided by the TCR for signalosome assembly together with the nanopore-stabilized kinetic segregation of TCR and CD45 in close contacts may lower the activation threshold, allowing residual Src-family kinase activity – even under inhibitory conditions – to phosphorylate ITAMs and initiate signalosome assembly,^64^ leading to NFAT nuclear translocation.^66^ In contrast, ERK activation requires sustained ZAP-70-mediated phosphorylation to overcome the digital Ras–ERK activation threshold,^67^ making it more Lck-dependent. On antibody-coated flat substrates, however, the thick neutravidin–biotin-antibody layer increases the membrane-surface gap and limits CD45 segregation.^68^ This raises the activation threshold, making it more dependent on a strong CD3-ligand engagement^24^ and full Src-family kinases activity. Furthermore, the curved membrane geometry of microvilli may facilitate lipid and protein organization, which is lacking on flat substrates.^42–44^

Nanopore confinement may also intersect with emerging mechanical models of TCR activation. Recent work suggests that TCR rigidity and mechanical compliance can modulate signaling thresholds by influencing conformational dynamics of the receptor and force transmission. In this context, nanopore-stabilized microvilli may impose lateral confinement, curvature-dependent membrane stress, and restricted spatial degrees of freedom on the TCR-CD3 complex.^50,69^ Such constraints could alter torsional strain, receptor mobility, or the mechanical coupling between extracellular and cytoplasmic domains. Membrane curvature and lipid organization within nanopores may directly enhance ITAM accessibility. The basic-rich sequences of CD3ε and ζ chains interact with anionic lipids like PIP_2_, anchoring them to the inner membrane leaflet.^63^ Nanopore-induced curvature could reduce these interactions by altering lipid packing and applying steric pressure, facilitating ITAM release into the cytosol.^43,64^ Moreover, increased calcium influx on *Nanopores* may enhance ITAM accessibility by neutralizing membrane charges and activating calcium-dependent signaling pathways such as the calmodulin-calcineurin axis.^70,71^ While this interpretation remains speculative, it places nanopore-mediated activation within a broader framework in which TCR signaling is regulated not only by ligand binding and kinetic segregation but also by receptor mechanics and membrane geometry.

Furthermore, membrane properties critically shape TCR signaling within confined contacts.^43,64^ We found that GsMTx4, an MSC inhibitor, reduced nanopore-induced ERK signaling at high concentrations. However, generally blocking calcium channels–including MSCs and SOCE channels–with gadolinium^54,55^ did not replicate this effect, suggesting that GsMTx4 acts through its membrane-active properties rather than channel inhibition. By inserting into the outer membrane leaflet, GsMTx4 alters bilayer tension and curvature,^58,59^ potentially disrupting protein clustering essential for the scaffolding functions of the TCR. Supporting this, MβCD, which extracts cholesterol and thereby destabilizes TCR signaling clusters,^60^ similarly reduced ERK activation. These findings highlight the importance of membrane properties and lipid organization in sustaining TCR signaling within nanopore-stabilized contacts.

Interestingly, chelation of extracellular Ca^2+^ with EDTA strongly reduced the level of nanopore-induced ERK phosphorylation and NFAT nuclear translocation. Ca^2+^ can directly interact with negatively charged glycocalyx components such as hyaluronic acid and other carboxylate-containing glycans, thereby altering polymer conformation, chain rigidity, and interfacial organization.^72,73^ In line with polyelectrolyte brush models, multivalent ions can induce structural reorganization and lateral inhomogeneities in charged surface-tethered polymers, affecting steric and electrostatic interactions at interfaces.^74^ However, our STED data showing patchy CD45 distribution even under EDTA treatment argue against a simple model in which Ca^2+^-dependent glycocalyx compression alone governs close contact formation. Instead, extracellular Ca^2+^ could further modulate membrane and protein mechanics, thereby interfering with protein clustering and conformational changes required for efficient TCR signal induction. However, the precise influence of extracellular calcium on nanopore-induced signal induction remains to be explored.

To explore the broader applicability of nanopore-induced activation, we showed that both human naive CD4^+^ and CD8^+^ T cells responded robustly to nanoporous stimulation, reaching activation levels comparable to conventional biochemical methods. This effect extended to pan T cell populations, including primary cells from DLBCL patients. Although nanopores generate a strong early activation phenotype, the qualitative differences in signaling compared to full biochemical stimulation are important for translation. Nanopores initiate TCR-downstream signaling independently of CD28 and therefore preferentially engage ERK and calcium-NFAT pathways, while NFκB activity remains limited. This pattern explains why nanopores robustly induce CD69 expression and transient proliferation but only modest IL-2 secretion in the absence of costimulation. CD28 normally amplifies TCR signaling, including strong NFκB activation, robust IL-2 production, and is required to drive the full functional program. Therefore, CD28 costimulation remains essential to drive the full functional program, including sustained effector differentiation, metabolic fitness, and long-term survival. When combining nanoporous stimulation with CD28 costimulation, activation approached levels seen with Dynabeads, underscoring the translational potential of nanopore-based stimulation. Thus, nanopores are best viewed not as a replacement for biochemical stimulation, but as a biophysical amplification platform that can be combined with defined costimulatory signals to tune T cell activation.

Despite the strong alignment between our data and the proposed close contact model, several limitations remain. Most notably, definitive mechanistic and structural evidence has still not been obtained. Direct imaging of molecular organization within nanopores remains technically challenging due to resolution constraints, especially along the *z*-axis,^75^ making it difficult to visualize protein segregation and membrane architecture along microvilli. The mechanistic and temporal details of protein exclusion, clustering, and lipid organization within pores of varying diameters require further investigation. Although we demonstrate robust activation across naive CD4^+^, CD8^+^, pan T cells, and patient-derived samples, fine-tuning costimulation through antibody coating to guide differentiation for immunotherapies will require further optimization, which lies beyond the scope of this study.

## Conclusion

This work demonstrates that nanoporous substrates can drive ligand-independent T cell activation by promoting stable close-contact formation through microvilli confinement, protein clustering, and membrane curvature–highlighting how extracellular topography shapes cellular responses. Furthermore, it introduces the concept of close-contact formation in a 3D environment, contrasting with prior models restricted to flat surfaces or microvilli tips. More broadly, these findings support a shift from a purely biochemical ligand–receptor paradigm to a biophysical model of T cell activation governed by cellular architecture and mechanical cues. Effective across both healthy and patient-derived T cells, this mechanism underscores the therapeutic potential of nanopore-based activation and broadens the design space for next-generation immunotherapeutic platforms.

## Experimental Section

### Substrate Optimization

Anodic aluminum oxide (AAO) substrates were sourced from Smartmembranes (Halle, Germany). The dimensions, pore size, and pore depth (5–10 μM in depth) were customized through close collaboration with the manufacturer to meet specific experimental requirements. Furthermore, Anodisc 0.2 μm, 13 mm (Whatman) substrates were used. To obtain pore diameter distribution for each substrate, the substrates were coated with 5 nm Pd/Pt with the Safematic CCU-010 Metal Sputter Coater. Scanning electron microscopy images were then taken with the Zeiss Merlin at the ScopeM facility of ETH and Hereon Teltow. Pore size diameters were analyzed using Fiji software (Supporting Figure 1a, details in Supporting Information). If not otherwise stated, custom-made substrates from Smartmembranes with medium mode pore diameter (∼244 nm) were used. As a flat control, untreated flat aluminum substrates from Smartmembranes were used (Supporting Figure 1b).

### Substrate Preparation

Substrates from Smartmembranes (10–11 mm diameter) were added to a 48-multiwell plate (mwp). 13 mm Anodisc substrates were used in 24 mwp. The substrates were cleaned sequentially for 15 min on a shaker using deionized H_2_O containing 0.1% Tween20, then 70% EtOH, and finally 100% Isopropanol, followed by overnight drying. Plasma cleaning was then conducted for 3 min using Henniker Plasma cleaner (HPT-100) or Zepto plasma cleaner (Diener Electronic). For the noncoated samples, PBS was directly added after plasma treatment. For antibody coating, 10 μg/mL Neutravidin (#31000, Thermo Scientific) or streptavidin (#434301, Thermo Scientific) in MES buffer (25 mM MES, 0.05% Tween20, pH 5) was added instead of PBS (more details in the Supporting Information). After 30 min of incubation of neutravidin/streptavidin on a shaker, the samples were washed twice with PBS before antibody solution, 5 μg/mL biotinylated anti-CD3 (clone OKT3, BioLegend, #317320) and/or biotinylated anti-CD28 (Clone CD28.2, Biolegend, #302904) in PBS, was added. The samples were incubated again for 30 min and washed 2× with PBS, followed by UV sterilization for 20 min for long-term experiments (24 h or more).

### T Cell Isolation

Anonymized buffy coat samples from healthy donors were obtained from Blutspende Zürich. PBMCs were then isolated using SepMate-50 (IVD) tubes (Stemcell) containing 15 mL of Density gradient medium (Lymphoprep, Stemcell) (details in the Supporting Information). PBMCs were immediately frozen after isolation (−80 °C) and stored long-term in a liquid/vapor phase nitrogen tank. PBMCs of diffuse large B-cell lymphoma patient samples were obtained from Inselspital Bern (Approved by Kantonale Ethikkommision Zürich, PB_2016–01011).

Prior to starting the experiment, PBMCs were thawed in a 37 °C water bath and then added to 0.25 mL PBS containing DNase (1 mg/mL, Merck, #DN25). Prewarmed T cell culture medium (RPMI 1640 (Gibco) supplemented with 10% FBS (Biowest, VWR, #S181H-500), 20 mM HEPES, 1× Glutamax, 1× Nonessential amino acids, 1× Sodium Pyruvate, 1× Penicillin–Streptomycin, and 1× β-mercaptoethanol (all obtained from Gibco)) was gently added to the cells dropwise to minimize osmotic stress. After centrifugation at 500*g* for 5 min, the supernatant was carefully removed. Cells were gently resuspended in the T cell culture medium with 0.1 mg/mL DNase. For T cell isolation, the Naive Pan T cell Isolation kit (Miltenyi Biotec, #130–097–095, only α/β T cells were isolated) was utilized, following the manufacturer’s protocol. For TCR knockdown experiments or patient versus healthy pan T cell experiments, the Pan T cell Isolation kit (Miltenyi Biotec, #130–096–535) was used instead. T cells were then resuspended in T cell culturing medium at a concentration of 1 × 10^6^ cells/mL, unless otherwise specified, and used within 1 day. In each experiment, donor specifications were newly assigned, with donors labeled as D1, D2, D3, and so on.

### CD69 Measurements and Antibody Staining for Flow Cytometry

For CD69 measurements, primary or Jurkat T cells were seeded at a concentration of 1 × 10^6^ cells/mL on specified substrates and incubated for 24 h at 37 °C and 5%CO_2_. To assess the influence of GsMTx4, naive pan T cells were preincubated with GsMTx4 (10 μM if not specified otherwise) for 30 min at 37 °C before seeding onto the substrates. Naive and resting cells were seeded into an empty well or directly used from the culture flask.

After 24 h incubation, T cells were transferred to 96-well V-bottom plates, washed, and stained at 4 °C with fluorochrome-conjugated antibodies against CD3, CD4, CD8, CD28, and CD69 in FACS buffer, followed by live/dead staining. The cells were either fixed with 4% PFA or directly analyzed on a Cytek Aurora or BD FACSymphony A1 flow cytometer. More details are given in the Supporting Information.

For analysis, FlowJo was used, with gating, as shown in Supporting Information Figure 17a. Per donor (or individual experiment for Jurkat T cells), the mean values of technical replicates were used for further analysis.

### Cytokine Release

Interleukin secretion was measured after 24 h of incubation using Lumit IL-2 (Human) Immunoassay according to the manufacturing protocol. Per donor, the mean values of technical replicates were used for further analysis.

### Phospho-Flow Cytometry

Naive PAN T cells (4 × 10^6^ cells/mL in 0.15 mL) were spun down onto substrates (1 min, 50*g*, slow acceleration and break) and incubated for specified durations at 37 °C. The cells were then washed off the substrates and fixed in 2% PFA for 10–15 min at 37 °C, before permeabilization on ice for 30 min using ice-cold methanol. Antibody staining for phosphorylated ERK or NFκB was then conducted in FACS buffer for 30 min at 4 °C. More details are given in the Supporting Information.

For early signaling measurements shown in Figure 2, measurements were done with a Cytek Aurora. The data were analyzed using FlowJo with gatings, as shown in Supporting Information Figure 18a. For the data shown in Figures 2 and Supporting Information Figure 4, the median fluorescent intensity values were normalized to the highest obtained value for each donor. Per donor, the mean values of the technical replicates were used for further analysis. Early signaling measurements using different inhibitors, as described in Inhibitor Assay: Nuclei Isolation Assay (NFAT) and ERK Phospho-Flow Assay section, were measured using FACSymphony A1, and the normalized median fluorescent intensity values were calculated as described in the section Inhibitor Assay: Nuclei Isolation Assay (NFAT) and ERK Phospho-Flow Assay.

### Inhibitor Assays: Nuclei Isolation Assay (NFAT) and ERK Phospho-Flow Assay

Primary human T cells (untreated or treated with inhibitors, as described in the Supporting Information) were seeded onto substrates (∼1Mio T cells/mL in 0.4 mL medium) by spinning 1 min 50*g* (slow acceleration and break) and incubated for 30 min at 37 °C, 5% CO2. 0.25 mL of T cells was washed off and added into a 96 V-bottom plate for nuclei isolation and 0.15 mL was used for phospho-flow cytometry, as described above. Nuclei were then isolated (more details are given in the Supporting Information) using the nuclei isolation buffers (NIB) A and B, before fixation using 4%PFA. After permeabilization, the nuclei were blocked with BSA and stained for NFATc2 using a fluorochrome-conjugated antibody. The nuclei were further stained with DAPI for clearer nuclei identification.

Using FlowJo, the flow nuclei events were gated using forward and side scatter, then for single cells and for nuclei using DAPI as an indicator. For phospho-flow cytometry, the events were gated with forward and side scatter and then for single cells. The median fluorescence values per sample were then further used. For normalization per experiment, a regression over the time point of recording was done for the naive samples as baseline and for the positive samples (only Flat+aCD3/28 or Nanopores together with Flat+aCD3/28 (as written in the figure captions)). After baseline regression subtraction, the samples were normalized to the positive regression curve (Supporting Information Figure 19a).

### T cell Expansion Assay

Primary human naive Pan T cells were labeled with CellTrace Violet (Thermo Fisher Scientific, #C34557) at a concentration of 5 μM in PBS. The cells were incubated with the staining solution for 20 min in a 37 °C water bath. After staining, the cells were washed twice with T cell medium. The stained cells were then seeded onto the indicated substrates at a concentration of 1 × 10^6^ cells/mL and incubated for 72 h at 37 °C in 5% CO_2_. The cells were washed off the substrates, transferred into fresh cell culture plates, and diluted with T cell medium as needed. Measurement of CellTrace Violet intensities was performed on day 7 after seeding using the FACSymphony A1 Analyzer.

The cell division cycles were then assessed using FlowJo. Manual gating was applied to separate the individual populations to obtain percentages of T cells per division cycle based on CellTrace Violet intensity. For each donor, the mean percentage per population of technical triplicates was used to calculate an overall mean and standard deviation. Additionally, the weighted-average division cycle was calculated from the percentages per division cycle.

### Microvilli Microscopy

For fluorescence microscopy of microvilli, naive T cells were spun down onto the substrates and incubated for 10 to 60 min, as indicated. The cells were then fixed on the substrates with 4% PFA. The membrane was then stained with CF488A wheat germ agglutinin (biotum) before permeabilization with Triton X-100 (0.1% in PBS with 1%BSA). After blocking, CD45, CD3, CD28, Lck, and phosphotyrosine were stained using primary and secondary antibodies. F-actin was stained by using phalloidin. After staining, the substrates were mounted with ProLong Glass Antifade Mountant with NucBlue Stain (Invitrogen, no. P36981). More details are given in the Supporting Information.

Imaging was performed using an Airyscan microscope (Zeiss LSM 880) with a 63*x*/1.40 Oil DIC M27 Plan-Apochromat objective, with 40 nm x-y pixel resolution at 184 nm z interval with the Airyscan detector. After Airyscan Processing with the ZEN black software Fiji was used to create maximum ad sum projections and ortho view images.

For structure illumination microscopy (SIM), the substrates were mounted with SlowFade Gold Antifade Mountant (Thermo Fisher Scientific, #S36936). Imaging was performed using a ZEISS ELYRA 7 system controlled by ZEN Black SR 3.0 software. More details are given in the Supporting Information. Imaging was supported by the Center for Microscopy and Image Analysis, University of Zurich. Details on STED microscopy are given in Supporting Information.

### NFAT Staining

Primary human naive Pan T cells were seeded on the indicated substrates in minimal amount of T cell medium, to allow fast settling onto the surfaces. The cells were incubated for 30 min at 37 °C and 5% CO_2_ before fixation with 4% PFA in cytoskeleton buffer (CSK: 10 mM MES, 150 mM NaCl, 5 mM glucose, 5 mM EGTA, 5 mM MgCl, pH 6.1) at 37 °C for 10 min. The PFA was then quenched with 50 mM ammonium chloride (NH_4_Cl) for 10 min at room temperature. After quenching, the cells were washed with PBS and permeabilized with 0.1% Triton X-100 in PBS for 10 min at room temperature. After washing with PBS, the samples were blocked (1% BSA in PBS) overnight or with 3% BSA for 1 h. The cells were then incubated with a primary anti-NFATC2 antibody (clone JA11–08, Thermo Scientific MA5–32661) in PBS with 1% BSA overnight at 4 °C. After washing, the samples were then incubated with a secondary antibody (antirabbit Alexa Fluor 568, Thermo Fisher Scientific #A-11036), along with DAPI (Serva, 18860.01) and Phalloidin conjugated to Alexa Fluor 647 (Thermo Fisher Scientific, #A22287) in PBS with 1% BSA. After washing, the samples were mounted and imaged using two different setups. First, the samples were mounted using VECTASHIELD Antifade Mounting Medium (vector, # H-1000–10) and imaged using the Leica SP8 confocal microscope with a HC PL APO 63*x*/1,40 OIL CS2 objective at 90 nm/pixel resolution. Second, the samples were mounted with Prolong Gold Antifade mounting medium and imaged using a spinning disc microscope built by Visitron using an Olympus FV1000 body with a Uplan FL 60x water immersion objective with a pixel size of 206 nm.

The acquired images were then analyzed by using Fiji. Single cells were selected using the actin channel in a semiautomated process through a custom Python script, employing automated thresholding with the “Li” algorithm, along with the particle detector tool, to segment the cells into individual regions of interest (ROIs). After the initial segmentation, the ROIs were manually reviewed, and adjustments were made, as needed, to ensure accurate single-cell selection. Background correction was then applied to each image by using the rolling ball algorithm with a ball radius of 50 pixels. Within each ROI, the nucleus was detected based on DAPI fluorescence intensity using the “Li” automated threshold and particle detector. Subsequently, the mean NFAT signal intensity inside and outside of the nucleus was measured for each cell, and the nuclear NFAT translocation was calculated by mean_nucleus_/mean_cytoplasma_.

### Intracellular Calcium Assessment with Flow Cytometry

Primary human naive Pan T cells were used to assess calcium influx. The cells were resuspended in Hank’s Balanced Salt Solution (HBSS) at a concentration of 2 × 10^6^ cells/mL and loaded with 4 μM Fluo-8 AM (Focus Biomolecules, #10–1322). The cells were incubated at 37 °C for 60 min. After incubation, the cells were washed and resuspended in HBSS supplemented with calcium and magnesium (Gibco, #14025050), followed by a resting period of 30 min at 37 °C. Yoda1 was added directly before seeding (final 20 μM, MedChem Express, #HY-18723). After resting, the cells were seeded onto substrates by centrifugation at 50*g* for 1 min and incubated at 37 °C for 10 min. The cells were then washed off the substrates and stained with 3 μM DAPI (Sigma-Aldrich, #D9564) in FACS buffer and subsequently measured with FACSymphony A1 Analyzer. For analysis, the Fluo-8 MFI values were normalized to the naive background control per measurement per replicate run.

### Live Calcium Imaging

Primary human naive Pan T cells were resuspended in HBSS (with calcium and magnesium) and stained with 4.5 μM Fluo-4 AM (Thermo Fisher, # F14201) at 1 × 10^6^ cells/mL for 1 h at 37 °C. Subsequently, they were washed one time and resuspended in T cell medium (no phenol red) and incubated for 30 min at 37 °C. If indicated, 10 μM GsMTx4 was added during this incubation step.

The substrates were preincubated with T cell medium for 30 min at 37 °C in custom imaging chambers. Just before the measurement, the medium was removed, and the cell suspension was added on top of the substrate and covered with a round coverslip. Imaging was performed at 37 °C on an upright Leitz Laborlux S microscope equipped with an HBO, an Andor Clara CCD camera, and an ibidi heating system. Images were taken for 30 min with 1 fps and 2 × 2 binning using a 10× objective. All of the steps above were performed without CO_2_ control.

Single-cell trajectories were obtained using Trackmate in Fiji. The calcium kinetics were further analyzed using custom-written Python-based software. Cells were classified as Ca^2+^ positive if the Fluo-4 signal was higher than a fold change of 2 and if the signal peak had sufficient steepness (fold change 0.015/s).

### Generation of Primary Human and Jurkat TCR Knockdown T Cells

Primary human Pan T cells were cultured for 48 h on porous substrates coated with anti-CD28 activating antibodies (clone CD28.2). Jurkat T cells, in contrast, were electroporated without prior activation.

For the TRAC knockdown, ribonucleoprotein (RNP) complexes were assembled using Alt-R S.p. Cas9 Nuclease V3 (IDT, #1081059) and a single-guide RNA (sgRNA) targeting the TRAC locus (5′-GGGAATCAAAATCGGTGAAT-3′). As a control reaction mix, no sgRNA was added to the nuclease. The RNP complexes were electroporated into the cells using the Invitrogen Neon Transfection System 10 μL Kit. Electroporation settings were optimized for each cell type: primary T cells were electroporated at 2100 V, 20 ms, 1 pulse, while Jurkat T cells were electroporated at 1325 V, 10 ms, 3 pulses. After electroporation, the cells were rescued in culture medium and incubated for a minimum of 3 days. TCR knockdown was then assessed by measuring CD3ε expression by flow cytometry using FACSymphony A1 Cell Analyzer (Supporting Figure 20a,b).

### Sorting and Validation of TRAC Knockdown

Jurkat T cells were sorted for CD3ε (SK7)-negative cells (∼0.5 × 10^6^ cells) using a BD FACSAriaIII cell sorter, available at the flow cytometry core facilities of ETH (Supporting Figure 21a). The loss of TCR expression (CD3-) in the sorted Jurkat T cells was confirmed by flow cytometry over multiple passages (>4) to ensure knockdown stability (Supporting Figure 21b,c)

### Lentivirus Production and Transduction

To produce lentivirus, targeting sequences against CD28 (5′-TTGTCGTACGCTACAAGCAT-3′) and nontargeting sequence (5′GGCCTGCCCTAAACCCCGGA-′3) for Cas9 gRNAs were cloned into the lentiCRISPR v2-Blast plasmid, as described by other studies.^76,77^ lentiCRISPR v2-Blast was a gift from Mohan Babu (Addgene plasmid # 83480; http://n2t.net/addgene:83480; RRID:Addgene_83480). HEK293T cells were cultured in DMEM medium (supplemented with 10%FBS 1× glutamine, and 1× penicillin/streptomycin) to achieve 70% confluence on the day of transfection. For transfection, 0.1 μg of pMD2.G plasmid, 0.9 μg of psPAX2 packaging plasmid, 1 μg of cloned lentiCRISPR v2-Blast plasmid, and 6 μL of X-tremeGENE DNA transfection reagent (Roche/Sigma-Aldrich, #6366244001) were added to 200 μL of DMEM (serum-free) and incubated for 20 min at RT, before being added dropwise to the cultured HEK293T cells. pMD2.G was a gift from Didier Trono (Addgene plasmid #12259; http://n2t.net/addgene:12259; RRID:Addgene_12259). psPAX2 was a gift from Didier Trono (Addgene plasmid #12260; http://n2t.net/addgene:12260; RRID:Addgene_12260).

After 48 h, the viral supernatant was collected, filtered through a 0.45 μm filter, and used to transduce 1 million Jurkat T cells. The cells were transduced at virus-to-cell volume ratios of 1:1 and 1:3 in the presence of polybrene (10 μg/mL) and subjected to spinfection (centrifugation at 1200*g*, 37 °C, for 1 h). After spinfection, the cells were incubated for 72 h before the medium was replaced with fresh T cell medium containing 5 μg/mL blasticidin for selection. The cells were selected with blasticidin for 2 weeks before further experiments.

### CD28 Knockdown Validation and Sorting

CD28-negative T cells were sorted by staining with a primary CD28.2 antibody, followed by detection with an Alexa Fluor 488-conjugated antimouse secondary antibody. Sorting was performed using a BD FACSAriaIII cell sorter at the flow cytometry core facilities of ETH (Supporting Figure 22a). The loss of CD28 expression was confirmed by flow cytometry over multiple passages (more than 4) to ensure stability of the knockdown (Supporting Information Figure 22b).

### Statistical Testing

If the same donors were present under two conditions, a paired *t* test was performed using the mean values of technical replicates per donor. If different donors were used for statistical testing, then an unpaired *t* test was performed on the mean values of technical replicates. Multiple comparisons were conducted using Benjamini–Hochberg (false discovery rate) adjustment. The number of donors (*n*_D_) used for testing can be taken out of the specific plot or can be taken from the Supporting Information Excel sheet, which also shows the exact *p*-values of each test.

## Supplementary Material









## Data Availability

The data that support the findings of this study are available from the corresponding author upon request.

## Notes

T cells extend microvilli into nanopores (Movie S1) (AVI)
Extension of microvilli into nanopores, signs of activation without the need for anti-CD3 and anti-CD28 antibodies (Movie S2) (AVI)
Statistical testing and exact *p*-values (ZIP)
Pore sizes of the different substrates: (1) pore diameter determination; (2) CD45 localization within medium-sized nanopores; (3) pore-size-dependent CD45 exclusion from nanopores; (4) NFκB signaling on nanopores is enhanced by CD28 stimulation; (5) activation of TCR knockdown T cells; (6) TCR knockdown inhibits pore-induced activation of Jurkat T cells; (7) CD28 is not involved in pore-induced activation; (8) nanopore induce store-operated calcium entry; (9) high concentration of GsMTx4 reduces nanopore-induced activation; (10) GsMTx4 reduces nanopore-induced calcium signaling; (11) microvilli formation into nanopores under influence of GsMTx4; (12) influence of pharmacological inhibition and TCR knockdown on early signaling; (13) CD45-low patches on microvilli imaged with STED; (14) extracellular calcium supports ERK phosphorylation on nanopores; (15) microvilli formation into nanopores under the influence of EDTA; (16) extent of activation of naive and Pan T cells; (17) exemplary gating strategy for CD69 measurements; (18) ERK and pNFκB phospho-flow gating; (19) pERK and NFAT signaling normalization and Flat controls; (20) TCR expression in primary human T cells after knockdown; (21) TCR KD Jurkat T cell generation; and (22) CD28 KD Jurkat T cells generation (PDF)
