# Supplementary material for: Close Contacts Unlocked: Nanopore-Stabilized Microvilli Bypass T Cell Receptor–Ligand-Dependent T Cell Activation
Source: ACS Nano. 2026 Jul 22;20(30):20961–78. doi: 10.1021/acsnano.5c16841 (PMC13450421; doi:10.1021/acsnano.5c16841)
Supplement: Supplementary file 1 [file nn5c16841_si_004.pdf]

Supplementary Information for:

Close-Contacts Unlocked: Nanopore-Stabilized Microvilli Bypass T Cell Receptor-Ligand Dependent T Cell Activation

Tamara Zünd[1], Sebastian Lickert[1], Willi Weber[2], Rafael Saxer[1], Marius Wenk[1], Janina Zünd[1], Tatiana Kovalchuk[1], Lucrezia Baldi[1], Dominic Aghaizu[2], Michael Daskalakis[3], Viola Vogel[1], Enrico Klotzsch[2,4, 5,*]

1 Laboratory of Applied Mechanobiology, Department of Health Sciences and Technology, ETH Zürich, Gloriastrasse 37-39, 8092 Zurich, Switzerland.

2 Institute for Biology, Humboldt Universität zu Berlin, Berlin, Germany.

3 Department of Hematology and Central Hematological Laboratory, Inselspital, Bern University Hospital, University of Bern, Bern, Switzerland

4 Berlin Institute of Health (BIH) at Charité – Universitätsmedizin Berlin, BIH Center for Regenerative Therapies (BCRT), Augustenburger Platz 1, 13353 Berlin, Germany.

5 Institute of Active Polymers, Helmholtz-Zentrum Hereon, 14513 Teltow, Germany

*Corresponding author: enrico.klotzsch@charite.de

Table of Contents

Supplementary Methods 1
Pore size determination using Fiji 1
Substrate coating: Neutravidin vs Streptavidin 1
PBMC isolation 1
Staining for flow cytometry 2
Early signaling assay: 2
Microvilli imaging 3
Supplementary Table 4
Supplementary Figures 5

Supplementary Methods

Pore size determination using Fiji

The SEM image of the nanopores was first converted to a binary format by applying a threshold distinguishing the pores from the background. To ensure accurate separation of adjacent pores, the watershed algorithm was applied. Subsequently, the "Analyze Particles" function (circularity 0.7-1) was used to identify and measure the area of each region of interest (ROI), corresponding to individual pores. The pore diameter was then calculated based on the area measurements, assuming a circular geometry for the pores.

Substrate coating: Neutravidin vs Streptavidin

Streptavidin was used for the following experiments: CD69 expression of naive primary T cells of donors 1 to 5 shown in Supplementary Fig.16a-c and further for donors 1 to 3 in early signaling experiments shown in Fig. 2a-d and Supplementary Fig. 4a-d. The performance of cells with Neutravidin coating was compared to that with Streptavidin coating, and both were found to exhibit equivalent performance. Therefore, Neutravidin was used in all other experiments. Furthermore, Neutravidin coating on flat aluminium substrates was shown to have no effect on early signaling compared to uncoated flat substrates (Supplementary Fig. 19b&c).

PBMC isolation

50ml of Buffy coat was diluted in PBS at a ratio of 1:4 and added to SepMate-50 (IVD) tubes (Stemcell) containing 15ml of Density gradient medium (Lymphoprep, Stemcell). The mixture was centrifuged at 1200rcf for 10min. The supernatant, containing the PBMCs, was carefully poured off, and the PBMC pellet was collected and washed with PBS by centrifugation at 120rcf for 10min and 300rcf for 8min. PBMCs were then resuspended in FBS at a concentration of 200 million cells/ml and aliquoted into cryo-vials ($100x10^6$ cells/vial). 0.5ml of 2X Freezing medium (20% DMSO/80% FBS) was added and the vials were immediately put to -80°C in a freezing container (Mr. Frosty) and transferred to liquid nitrogen the following day.

Staining for flow cytometry

After incubation on the substrate for 24h, T cells were transferred to 96 V-bottom plates, washed with FACS buffer (PBS with 0.5% BSA and 2mM EDTA) and stained for 30min at 4°C with the required antibody staining solution in FACS buffer (anti-CD69 (clone FN50-APC or -PerCP-Cy5.5, BioLegend), anti-CD3 (clone SK7-PE or -BV785, BioLegend or clone SK7-BUV395, BD), anti-CD4 (clone A161A1-FITC, BioLegend), anti-CD8 (clone SK1-Pacific Blue or -PE/Cy7, BioLegend or clone HIT8a-BUV805, BD), anti-CD28 (CD28.2 unconjugated, BioLegend)). For CD28 staining, primary staining was followed by secondary antibody staining (goat-anti mouse IgG, Alexa Fluor 488, Thermo Scientific, #A-11001) in FACS buffer. The cells were then washed with PBS and stained with live/dead stain in PBS (Live dead NIR, Thermo Scientific, Zombie NIR, BioLegend or Zombie Aqua BioLegend). Following staining, cells were washed with PBS containing BSA and subsequently either fixed with 4% PFA for 10min or directly analyzed using the flow cytometers Cytek Aurora (at FCCF) or BD FACSymphony A1 Cell Analyzer.

Early signaling assays:

Treatment with inhibitors. Naive primary human T cells were treated with inhibitors in T cell medium (GsMTx4 at 10 or 30 µM (Hölzel, #A16103-5), BTP2 at 5 µM (YM-58483, MedCehm Express, #HY-100831), PP2 10 µM (MedCehm Express, #HY-13805), Syntha-66 2 µM (MedCehm Express, #HY-111325), Gadolinium(III) chloride hexahydrate (Gd^{3+}) 1 mM (high concentrations due to precipitation in T cell medium, Thermo Scientific Chemicals, #011286.06), 3-Nitrocoumarin at 10 µM (MedCehm Express, #HY-111919)). The cells were then directly used for the assay after 30 min of incubation with inhibitor without medium exchange. T cells were incubated for 25 min with BAPTA-AM at indicated concentrations (Invitrogen, #B1205) or methyl-β-cyclodextrin at 8 mM (Merck, #332615-5G) in T cell medium before the medium was replaced with inhibitor-free T cell medium just before seeding. T cell medium with 2 mM EDTA (Thermo Fisher Scientific, #AM9260G) or EGTA 1 mM with additional 0.6 mM Magnesiumsulfate anhydrous in T cell medium or HBSS (no calcium, no magnesium) with additional magnesium-sulfate or calcium nitrate tetrahydrate at indicated concentrations was added to the T cells just before seeding.

Phospho-flow protocol. Naive PAN T cells were incubated on the substrates at $4x10^6$ cells/ml in 0.15ml cell culture medium for specified duration at 37°C. The cells were then washed off the substrates and added directly to 4% PFA in PBS in a 1:1 ratio (final 2% PFA) in a 96 deep-well plate. The cells were then fixed for 10 – 15min at 37°C. After washing with PBS, the cells were permeabilized by adding 1ml of ice-cold methanol stepwise to the sample while vortexing. After incubation for 30min on ice, the cells were washed thoroughly with PBS and transferred to a precoated V-bottom plate (BSA or FBS coating to avoid sticking of the cells to the plate). Antibody staining was then conducted in a 96 V bottom plate in FACS buffer for 30min at 4°C (NF-κB p65 (pS529) Clone K10-895.12.50, BV421, BD and anti-ERK1/2 (pT202/pY204), Clone 20A, Alexa Fluor 488, BD). The cells were then washed again using MACS, before cytometric measurement. A flat substrate control was tested as an aluminum control (Supplementary Fig. 19b).

Nuclei isolation Buffer (NIBA) was prepared by adding HEPES (10mM), KCl (10mM), MgCl2 hexahydrat (3mM) and DTT (0.1mM) to H2O (stored at 4°C). A 2X NIBB was prepared by adding 0.3% NP40 to NIBA buffer and pre-cooled until use.

Nucleus isolation protocol. Primary human T cells (untreated or treated as described in supplementary Information) were seeded onto substrates (~1Mio T cells/ml in 0.4ml medium) by spinning 1min 50xg (slow acceleration and break) and incubated for 30 min at 37°C, 5%CO2. 0.25ml T cells were washed off for nuclei isolation and added into a 96 V bottom plate pre-coated with 5% FBS in PBS (FBA buffer). The plate was spun at 600xg for 3 min and the supernatant was blotted. The pellet was resuspended in 100µl NIBA. 100µl NIBB was added

quickly and sealed with adhesive plate seal foils before vortexing with 15s. After 2.5min on ice the plate was vortexed again 15s and spun down at 800xg for 3 min (further used). After blotting the pellet was resuspended in pre-cooled 4% PFA and incubated for ~12min at 4°C. 100µl FBS buffer was added before spinning. 150µL supernatant was aspirated, leaving 50µL inside the wells to leave the pellet undisturbed (not blotting at this point, as the pellet is unstable). The pellet was washed twice in the FBS buffer. The pellet (with 50µl left over supernatant) was resuspended with 50µL 2X Permeabilization buffer (0.2% Triton-X-100, 2% BSA in PBS) and incubated for 30 min. 100µL FBS buffer was added, before spinning, and blotting. The cells were blocked for 30 min at RT by resuspending the pellet first in 100uL 3% BSA in PBS. 100µl FBS buffer was added and after spinning, 170µl supernatant was aspirated leaving 30µl supernatant. The pellet was the resuspended with 30µl 2X staining buffer (1 to 250 NFATC2 Polyclonal Antibody, CoraLite® Plus 488 (Proteintech, #CL488-22023), in 1%BSA in PBS) and incubated overnight. The next day the Cells were washed 3 times, always leaving the pellet undisturbed by leaving 150ul during aspiration. Before measurement with FACSymphony A1, DAPI was added (final ~2ug/ml). A flat substrate control was tested as an aluminum control (Supplementary Fig. 19c).

Microvilli imaging

After incubation on the substrates, the supernatant was carefully removed and the cells were fixed on the substrates using prewarmed 4% PFA for 10min at 37°C. After washing twice with PBS, PFA was quenched using 50mM NH4Cl. To stain the membrane, CF488A Wheat Germ Agglutinin (biotum) was added after the cells were fixed. For intracellular staining, the cells were permeabilized using 0.1% Triton-X-100 with 1% BSA in PBS before blocking. Blocking with 3%BSA in PBS was performed for at least 30min before antibody staining solution in PBS with 1% BSA was added (Anti-CD45 (clone F10-89-4, abcam, ab30470), Anti-CD3 (clone CD3-12, abcam, ab11089), Anti-CD28 (clone EPR22076, abcam, ab243228), Anti-Lck (clone Y123, abcam, ab32149) or Anti-Phosphotyrosine (clone PY20, BioLegend, #309302)). After washing with PBS, secondary antibody staining solution was added in PBS with 1% BSA for 30min (DyLight 649 AffiniPure Donkey Anti-Rat IgG H&L (Jackson ImmunoResearch), Alexa Fluor 647 Donkey Anti-Rabbit IgG H&L (abcam, ab150063) or Alexa Fluor 647 Donkey Anti-Mouse IgG H&L (abcam, ab150107)). For actin staining, permeabilized cells were incubated with phalloidin conjugated to Alexa Fluor 488. After washing, the substrates were mounted with ProLong Glass Antifade Mountant with NucBlue Stain (Invitrogen, #P36981).

SIM Imaging. Imaging was performed using a ZEISS ELYRA 7 system controlled by ZEN Black SR 3.0 software. Data of Alexa Fluor 488 and Alexa Fluor 647 was acquired simultaneously on a ZEISS Elyra 7 system with Duolink (two 4.2 CL HS pco.edge sCMOS cameras) using the 488 nm and 642 nm laser lines, ZEISS Plan-Apochromat 63x/1.4 Oil DIC M27 objective, 13 phases, 80 ms exposure time and a z-stack of 93 slices with a voxel size of $0.06 \times 0.06 \times 0.13 \mu m^3$. Beam splitting was achieved using a main beam splitter (LBF 405/488/561/642) and a secondary beam splitter (SBS BP 490-560 + LP640). SIM image reconstruction was performed in ZEN Black SR 3.0. Chromatic aberration calibration was conducted using a z-stack of 200nm TetraSpeck microspheres. Affine corrections were applied using the ‘Channel Alignment’ tool in ZEN Black SR 3.0, achieving precise alignment between 488nm and 642nm channels. Calibration templates specific to the objective and filter configurations were stored for subsequent use in experimental corrections.

STED immunocytochemistry Fixed T cells were incubated in blocking buffer (1x PBS supplemented with 5 % BSA (Sigma)) for 1 hr at RT. Blocking buffer was then replaced with primary antibodies diluted in blocking solution and samples were incubated at 4°C overnight. Primary antibodies used in this study were: LCK (1:200,clone Y123, abcam, ab32149), CD3 (1:100, clone CD3-12, abcam, ab11089) and CD45 (Sigma, YAML501.4, RRID:AB_2539077, 1:100). T cells were then washed 3 x for 5 min each with PBS and subsequently incubated with secondary antibodies diluted in blocking solution for 2 hrs at RT in the dark. Secondary antibodies used were: goat anti rabbit Star Red (Abberrior, STRED-1002-500UG, RRID:AB_ 2833015, 1:500) and goat anti rat Star Orange (Abberrior, STORANGE-1007-500UG, RRID:

AB_2833017, 1:500). Samples were then washed 3 x for 5 min each with PBS at RT in the dark and mounted using Prolong gold antifade reagent (Molecular Probes) or SlowFade Glass Soft-Set Antifade Mountant.

STED microscopy. Samples were imaged on three different microscopes. For Supplementary Fig. 2c and 15b the Abberior STED Facility Line STED microscope fitted with an UPLSAPO 100x oil immersion objective, NA 1.4 (Olympus) was used. Excitation was provided by pulsed diode laser PDL-T 561 for imaging Star Orange dye and pulsed diode laser PDL-T 640 for imaging Star Red dye. Depletion for both the red (Star Orange) and far-red channels (Star Red) was accomplished using a pulsed STED laser-40-3000-775-B1R 775 nm laser. Excitation and depletion lasers were set individually depending on the signal intensities. Images were acquired with the pinhole size set to 0.8–1.0 airy units (AU), the pixel dwell time to 5 µs, a pixel size of 35 × 35 × 80 nm (*xyz*), and a z-stack slice interval of 80 nm. For Fig. 5b and Supplementary Fig. 13 the Leica SP8 inverse STED microscope was used (Leica DMI6000B, Model SP8) with a 93x, NA 1.3, Glycerol objectiv (HC PL APO STED WHITE - motCORR). The pinhole size was set to 1.0 airy units (AU). For excitation a white light laser (WLL) was used with 580 nm and 633 nm excitation wavelengths. For depletion a 775 nm pulsed STED laser was used. Pixel size was set to 20 nm.

Supplementary Table

Substrate	Median	Mode	Mean
Small	123	139	122
Medium	239	244	234
Large	331	353	324
Extra large	335	348	327
Anodisc topside	107	111	108
Anodisc backside	229	226	224

Supplementary Table 1: Median, mode and mean pore diameter [nm], calculated from pore areas measured in Fiji from SEM images.

Supplementary Figures

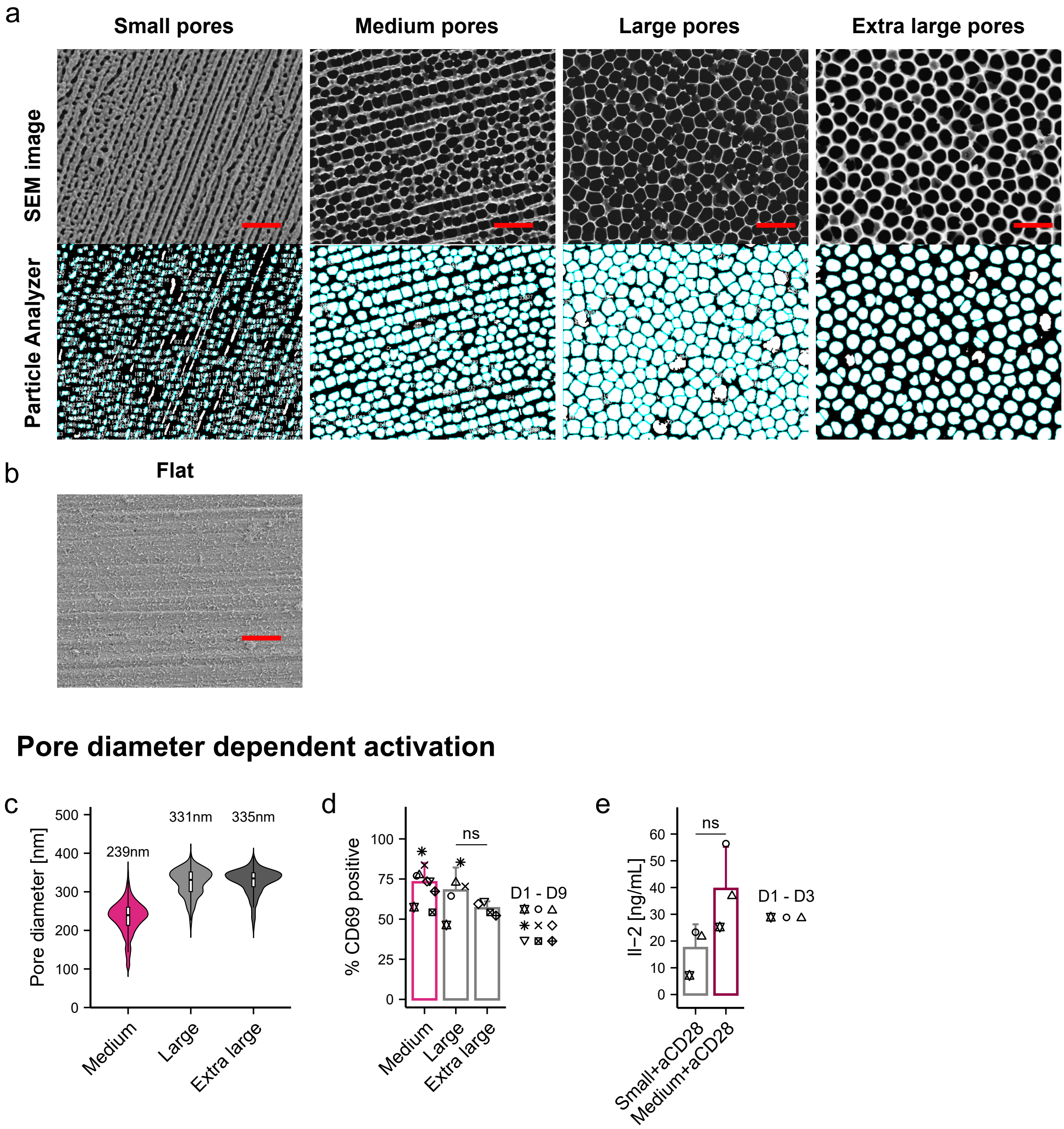

Supplementary Figure 1: Pore diameter determination: a) Nanopore diameter of aluminum oxide substrates were analyzed using SEM images of the respective substrates. The diameter was determined using Fiji by first using a threshold to determine the pores, then a watershed algorithm was applied to separate connecting pores. The area of each pore was determined using the particle analyzer (circularity 0.7-1). The diameter was calculated from the area assuming circularity. Scale bar in red = 1 µm. b) Flat aluminum substrate used as control to the nanopores. Scale bar in red = 1 µm. c) Violin plot showing the distributions of nanopore diameters in anodic aluminum oxide substrates with varying pore sizes. The median pore diameter values are indicated above the violin plots. d) Activation induced in naive primary human T cells ($CD8^+$ and $CD4^+$), shown as the percentage of $CD69^+$ T cells, 24 h after seeding on the indicated substrates, measured by flow cytometry. e) Naive primary human T cells were seeded onto indicated substrates and incubated for 24h. Absolute IL-2 secretion was then measured using the Lumit Promega assay. d&e) Bars indicate the mean ± SD across donors (D; d: n_D = 4-9, e: n_D = 3). Symbols represent mean values of technical replicates per donor. Statistical analysis was performed using an unpaired t-test on per-donor mean values (d) or a paired t-test on per-donor mean values from donors present in both conditions (e). Multiple comparisons were corrected using the Benjamini-Hochberg false discovery rate (FDR) method.Significance is indicated as follows: ns, $p \geq 0.05$.

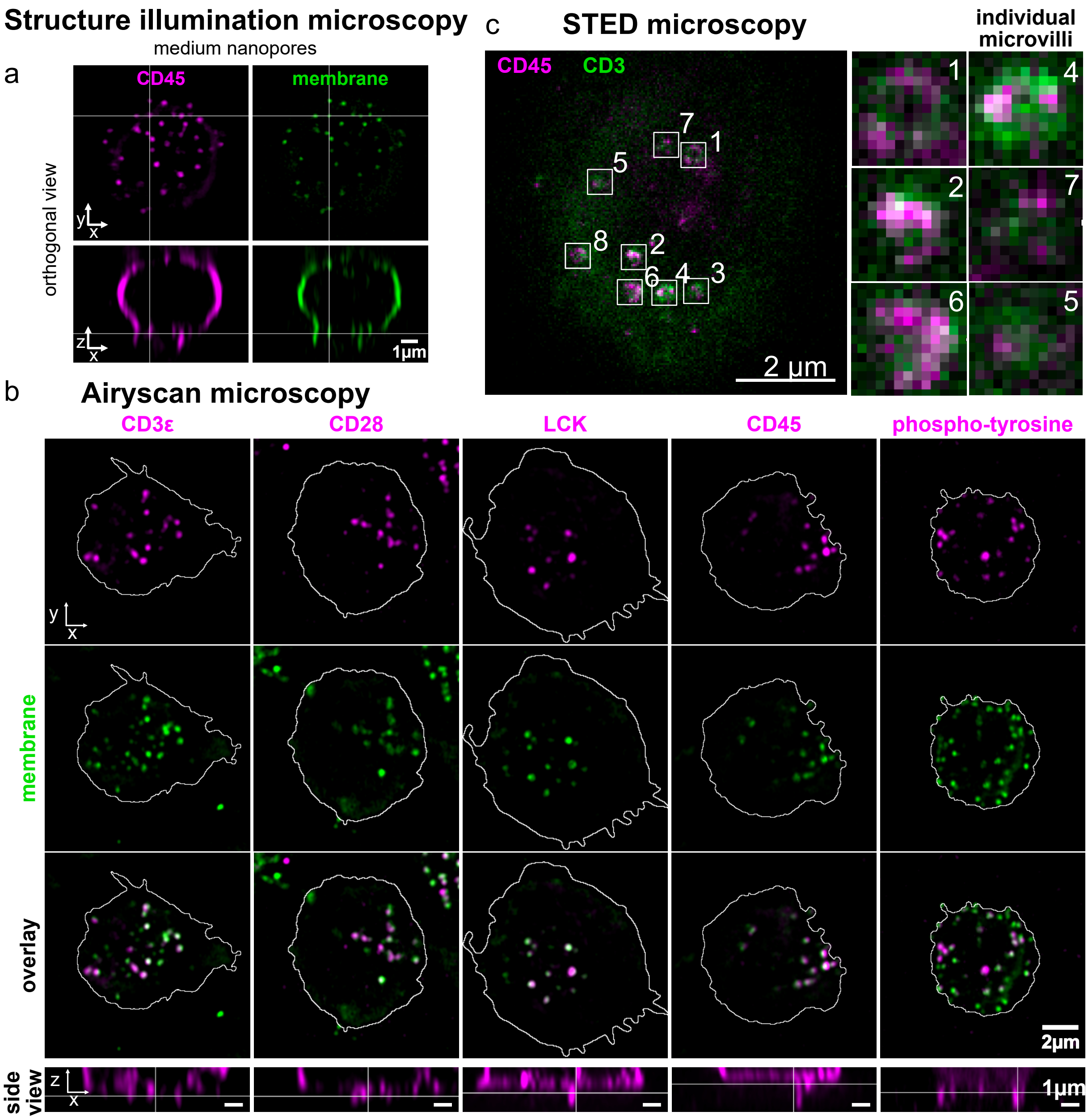

Supplementary Figure 2: CD45 localization within medium-sized nanopores: a) Orthogonal view of a primary human T cell on medium-sized nanopores stained for CD45 (magenta) and plasma membrane (WGA, green), imaged using structured illumination microscopy (SIM). b) Airy scan microscopy images of representative T cells on medium-pore-sized substrates stained for the indicated proteins (CD3, CD28, LCK, CD45, phospho-Tyrosine - all in magenta) and WGA as membrane stain. Shown are maximum projections of the stains across z-slices within the nanopores. The white line outlines the shape of the T cell above the nanoporous substrate. A separate x-z cross-section below the projection shows protein localization within the vertical axis of the nanopores. c) z-slice of microvilli from a single cell within medium-sized nanopores imaged using STED microscopy, showing CD45 (magenta) and CD3 (green). Boxes highlight individual microvilli, with selected ones shown in close-up views.

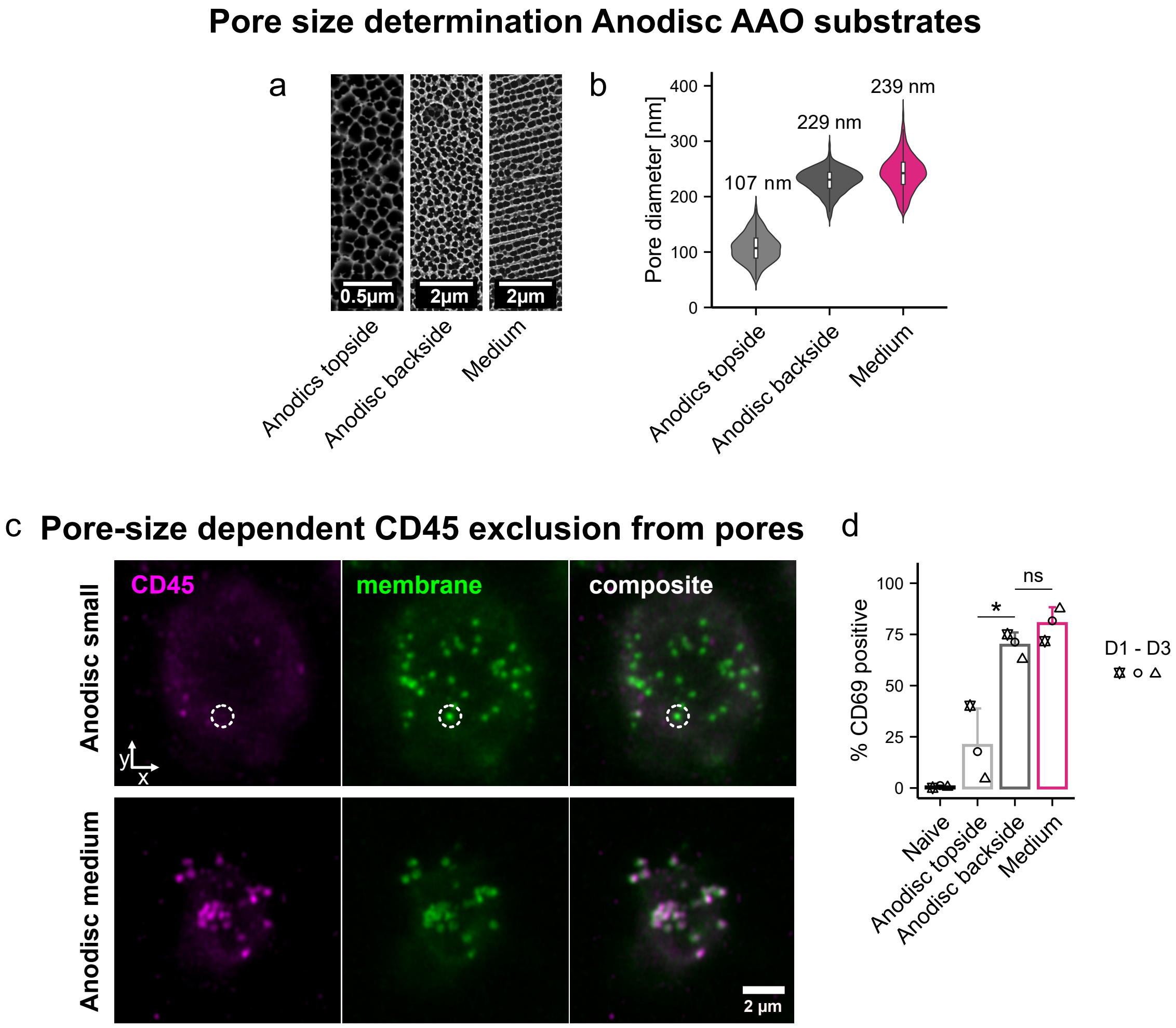

Supplementary Figure 3: Pore-size dependent CD45 exclusion from nanopores. a) SEM images of small- (107 nm) and medium-sized (229 nm) median nanopore diameters of anodisc AAO substrates (top- and backside) and medium-sized custom AAO substrates. b) Violin plot of pore diameters of substrates shown in a. The mode pore diameter is shown above the violin plot. Cross-section (x-y) of Airyscan microscopy images of representative T cells on Anodisc nanoporous substrates with small- and medium-sized median nanopore diameters. CD45 (magenta) is visualized via antibody staining and the membrane is stained using WGA (green). The dotted circle marks a microvilli with CD45 excluded fron the pore. Scale bar = 2µm. Plots are isotropic in x, y and z direction. d) Percentage of CD69 positive T cells 24h after activation on small- and medium sized anodisc substrates and medium-sized custom substrates. Bars indicate the mean ± SD across donors (D; $n_D = 3$). Symbols represent the mean value of technical triplicate for each donor. Statistical analysis was performed on per-donor mean values using a paired t-test with Benjamini-Hochberg (FDR) adjustment for multiple comparisons. Significance symbols represent adjusted p-values: ns : $p \geq 0.05$, * : $p < 0.05$.

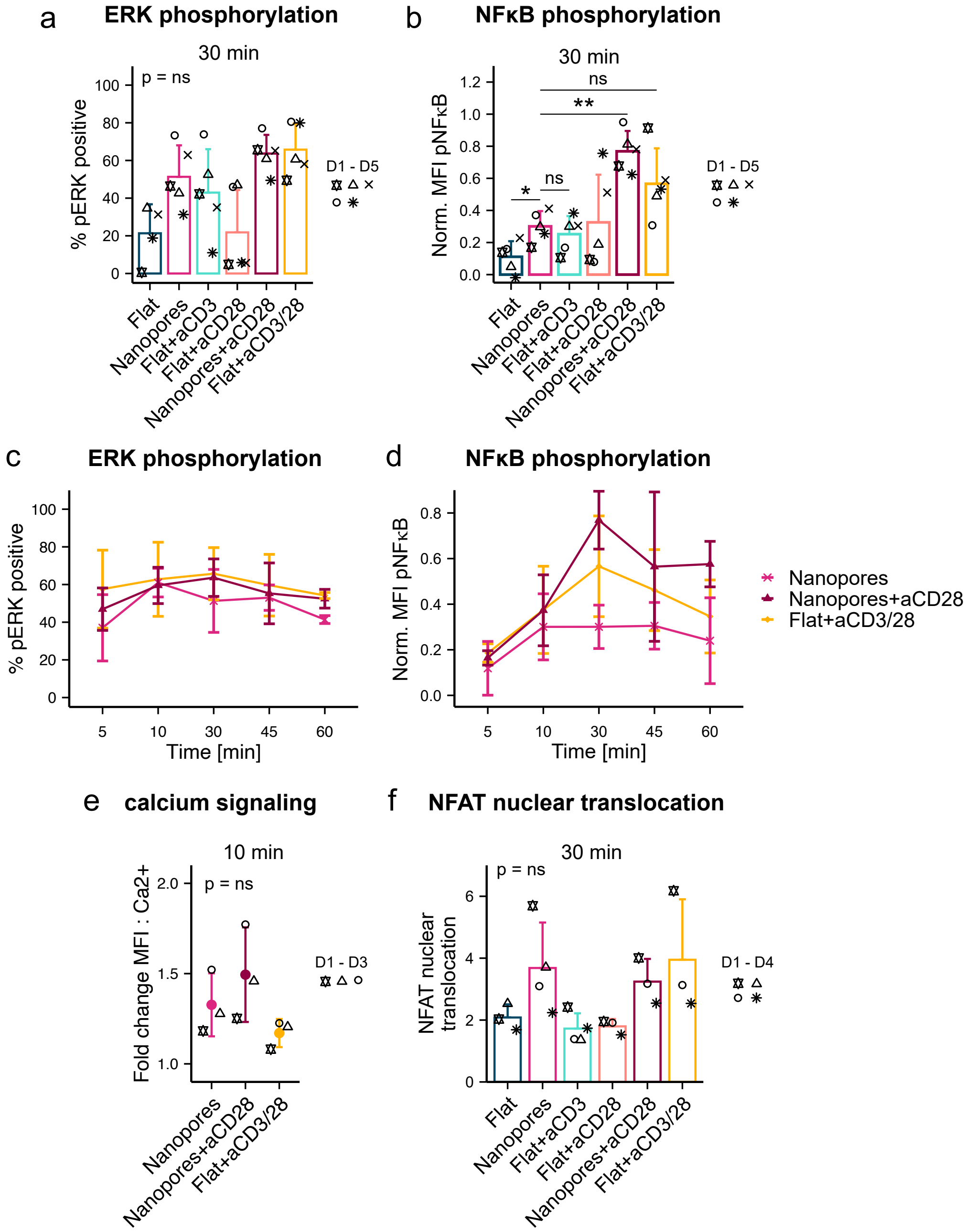

Supplementary Figure 4: NFκB signaling on nanopores is enhanced by CD28 stimulation. a) Percentage of T cells expressing phosphorylated ERK (pERK) after 30 min incubation on the indicated substrates, measured by flow cytometry. b) Median fluorescence intensity (MFI) of phosphorylated NFκB (pNFκB) after 30 min incubation on the indicated substrates, measured by flow cytometry. Values are normalized per donor to the highest value over time after background subtraction (naive condition used as background). c) Percentage of T cells expressing pERK over time on indicated substrates, measured by flow cytometry. d) MFI of pNFκB over time on the indicated substrates, measured by flow cytometry. Normalized as described in b. e) Fluo-8 MFI fold change relative to naive, as a calcium indicator after 10 min of incubation on different substrates, measured via flow cytometry. f) Nuclear NFATc2 translocation ratio (mean nuclear intensity / mean cytoplasmic intensity), calculated from confocal microscopy images. a&b&f) Bars indicate the mean ± SD across donors (D; a&b: n_D = 3-5; e: n_D = 3; f: n_D = 3-4). c-e) Points indicate the mean ± SD across donors (n_D = 3-5). a&b&e&f) Symbols indicate mean values of technical replicates for each donor. a-f) Statistical analysis was performed on per-donor mean values from donors present in both conditions using a paired t-test with Benjamini-Hochberg (FDR) adjustment for multiple comparisons. Significance symbols represent adjusted p-values: ns : $p \geq 0.05$, * : $p < 0.05$; ** : $p < 0.01$.

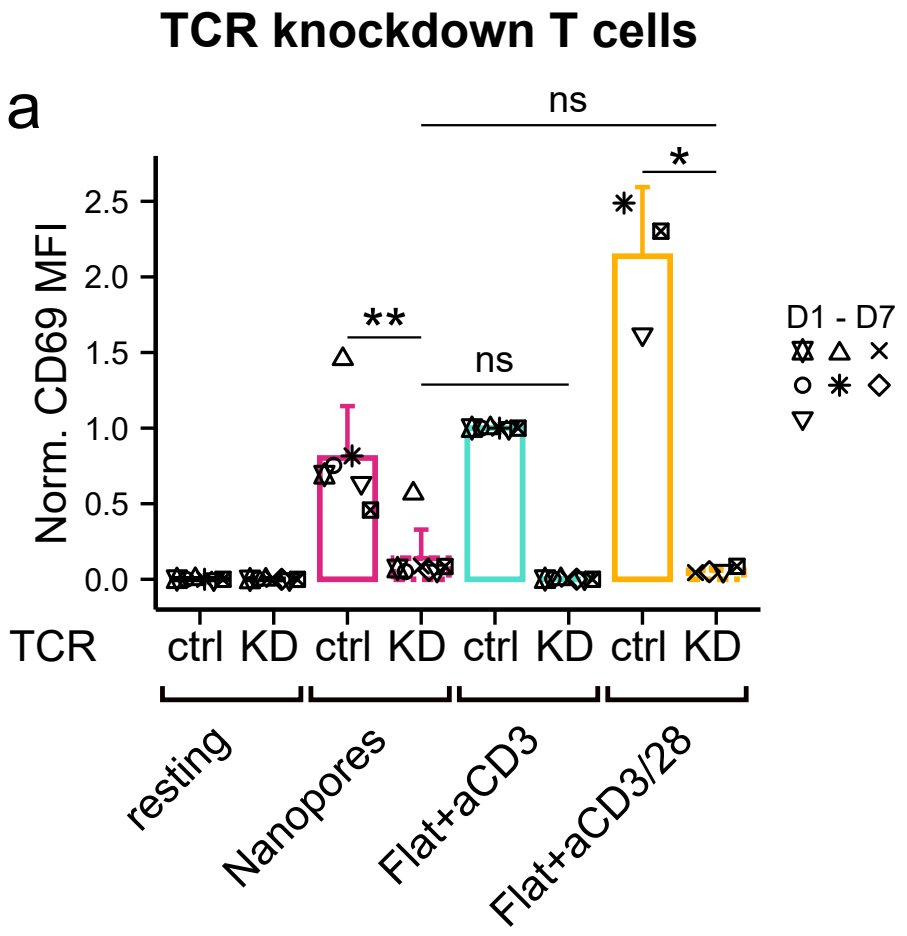

Supplementary Figure 5: Activation of TCR knockdown T cells. a) CD69 expression was measured by flow cytometry in TCR knockdown (KD, $CD3^-$ T cells) and untargeted TCR control (ctrl, $CD3^+$ T cells) primary human T cells after 24 hours of re-stimulation on the indicated substrates. CD69 MFI normalized to Flat+aCD3 TCR control T cells after baseline subtraction (resting T cells). Bars indicate the mean ± SD across donors (D, n_D = 3-7). Symbols indicate mean values of technical replicates for each donor. Statistical analysis was performed on per-donor mean values from donors present in both conditions using a paired t-test with Benjamini-Hochberg (FDR) adjustment for multiple comparisons. Significance symbols represent adjusted p-values: ns : $p \geq 0.05$, * : $p < 0.05$; ** : $p < 0.01$.

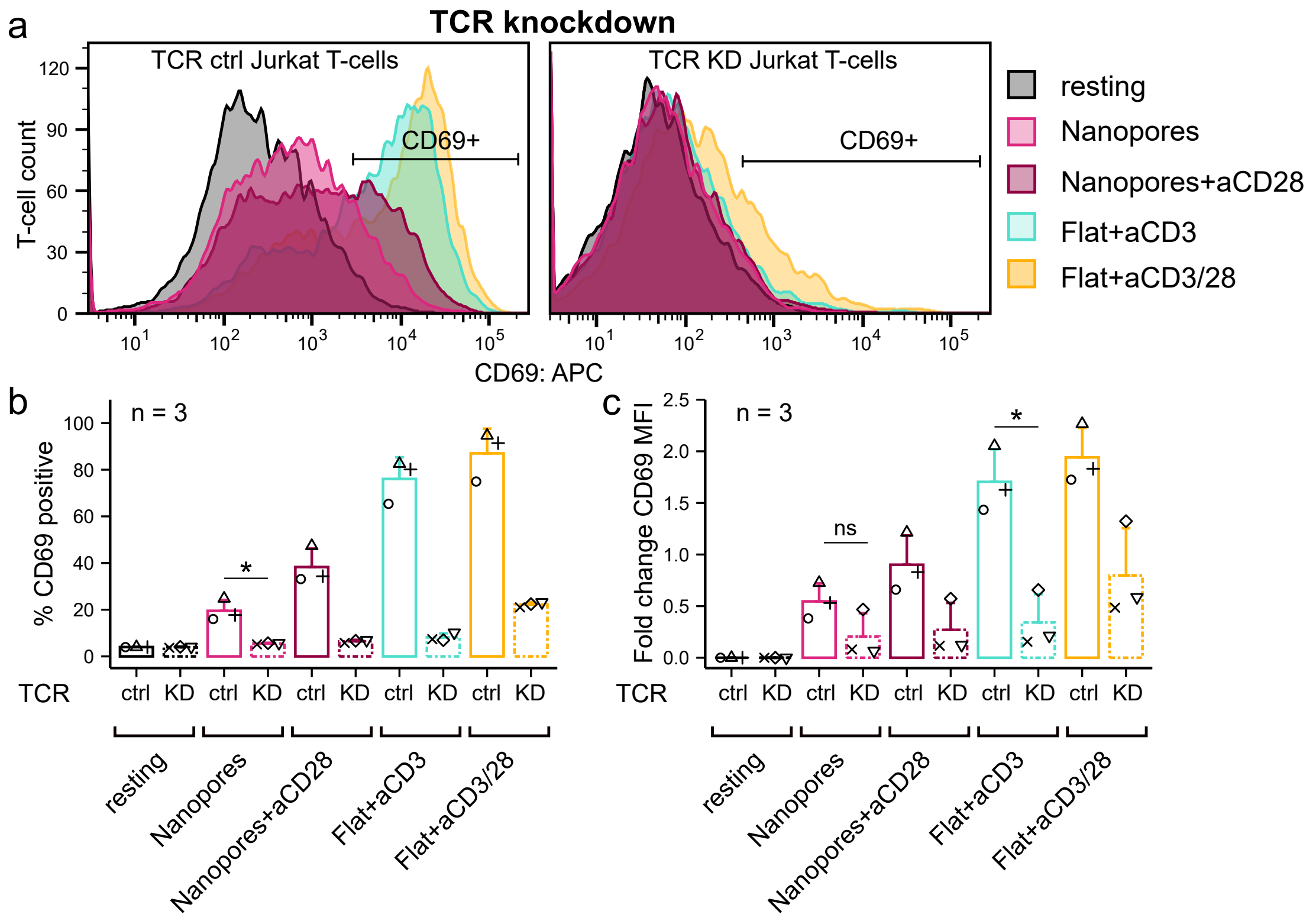

Supplementary Figure 6: TCR knockdown inhibits pore-induced activation of Jurkat T cells. a-c) CD69 expression in TCR control (ctrl, $CD3^+$) and sorted TCR-knockdown (KD, $CD3^-$) Jurkat T cells 24h after incubation on indicated substrates. a) Exemplary flow cytometry histograms. b) Percentage of $CD69^+$ cells. c) CD69 MFI fold change compared to resting Jurkat T cells of the respective group. b&ca-f) Bars indicate the mean ± SD across experiments (n = 3). Symbols indicate mean values of technical replicates for each experiment. Statistical analysis was performed on per-donor mean values using an unpaired t-test with Benjamini-Hochberg (FDR) adjustment for multiple comparisons. Significance symbols represent adjusted p-values: ns : $p \geq 0.05$, * : $p < 0.05$.

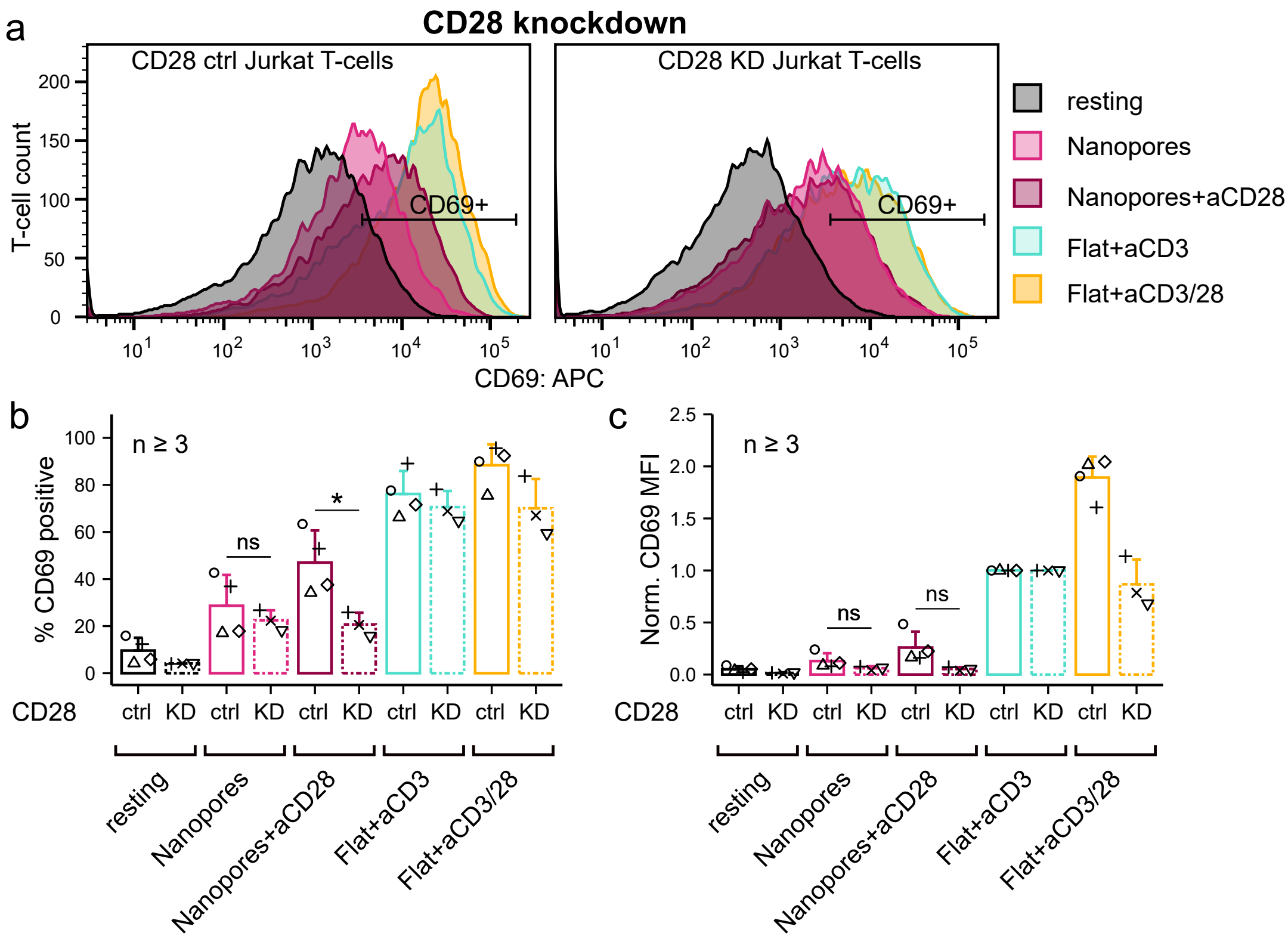

Supplementary Figure 7: CD28 is not involved in pore-induced activation. a-c) CD69 expression in CD28 control (ctrl, $CD28^+$) and sorted CD28-knockdown (KD, $CD28^-$) Jurkat T cells 24h after incubation on the indicated substrates. Symbols indicate mean values of technical triplicates from individual experiments. a) Exemplary flow cytometry histograms. b) Percentage of $CD69^+$ cells. c) CD69 MFI normalized to Flat+aCD3 of the respective group. b&c) Bars indicate the mean ± SD across experiments (n= 3-4). Symbols indicate mean values of technical replicates for each experiment. Statistical analysis was performed on per-donor mean values using an unpaired t-test with Benjamini-Hochberg (FDR) adjustment for multiple comparisons. Significance symbols represent adjusted p-values: ns : $p \geq 0.05$, * : $p < 0.05$.

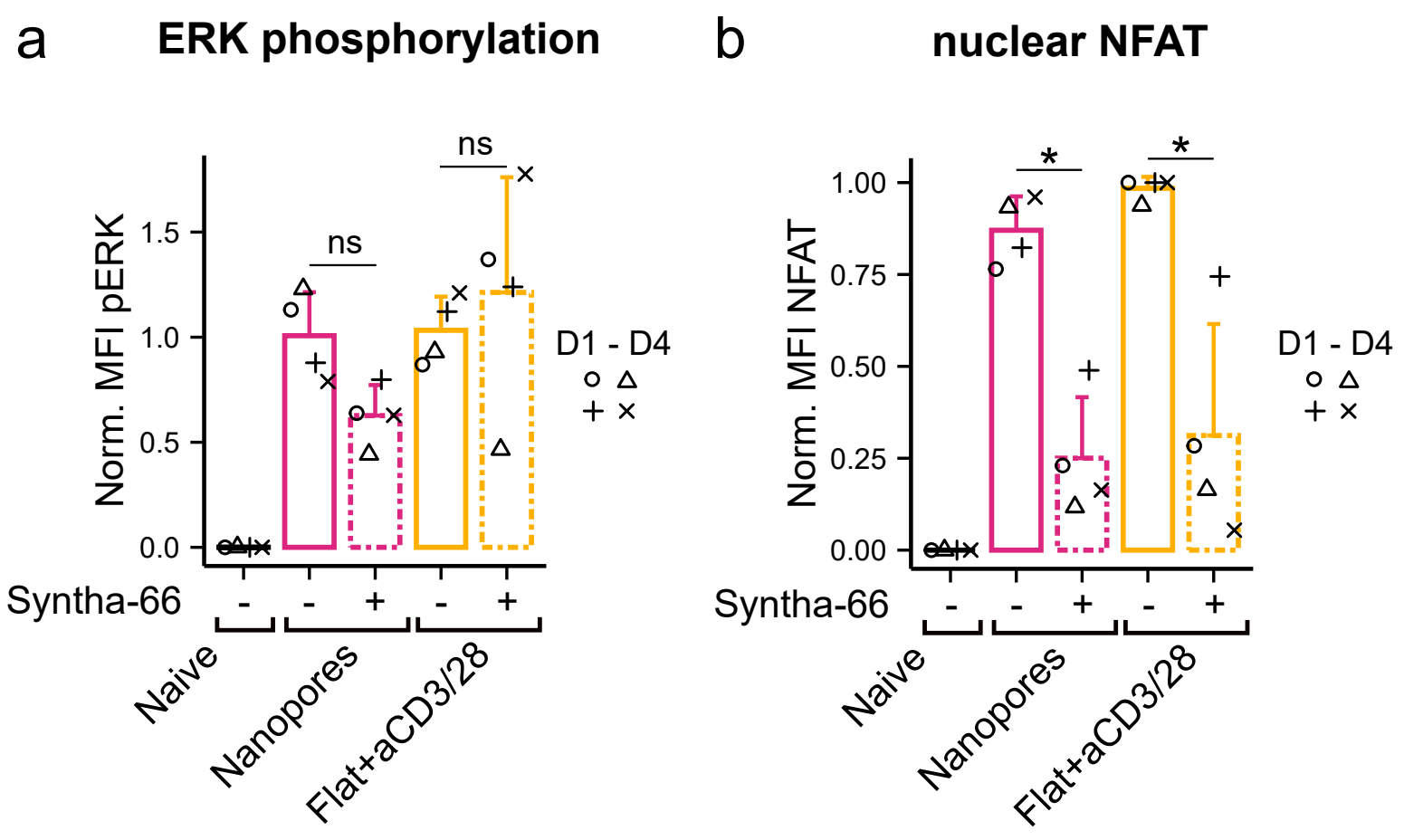

Supplementary Figure 8: Nanopore induce store-operated calcium entry. a&b) Naive primary human T cells were pre-incubated for 30 min with Syntha-66 (2 µM) prior to incubation for 30 min on the indicated substrates. a) MFI of phosphorylated ERK (pERK) measured by flow cytometry, normalized per donor to the positive control sample (Flat + aCD3/28 without inhibitors) and naive as baseline. b) NFAT MFI in isolated nuclei measured by flow cytometry, normalized per donor to both positive control samples (Flat + aCD3/28 and Nanopores) and naive as baseline. a&b) Bars indicate the mean ± SD across donors (D; $n_D = 4$). Symbols indicate mean values of technical replicates for each donor. Statistical analysis was performed on per-donor mean values from donors present in both conditions using a paired t-test with Benjamini-Hochberg (FDR) adjustment for multiple comparisons. Significance symbols represent adjusted p-values: ns : $p \geq 0.05$, * : $p < 0.05$.

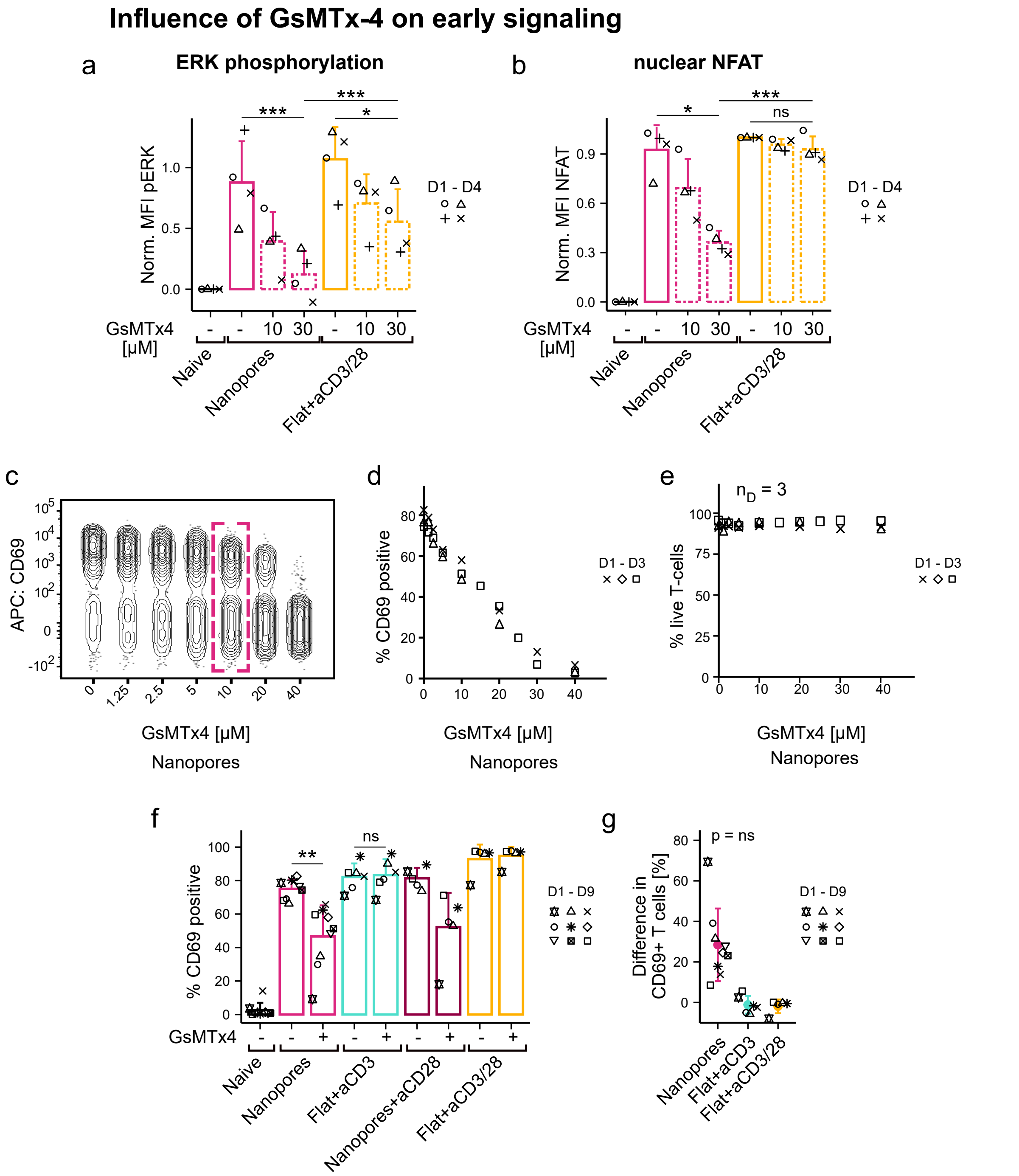

Supplementary Figure 9: High concentration of GsMTx4 reduces nanopore-induced activation. a&b) Naive primary human T cells were pre-incubated for 30 min with the indicated concentration of GsMTx4 prior to incubation for 30 min on the indicated substrates. a) MFI of phosphorylated ERK (pERK) measured by flow cytometry, normalized per donor to the positive control sample (Flat + aCD3/28 without inhibitors) and naive as baseline. b) NFAT MFI in isolated nuclei measured by flow cytometry, normalized per donor to both positive control samples (Flat + aCD3/28 and Nanopores) and naive as baseline. a&b) Bars indicate the mean ± SD across donors (D; $n_D = 4$). c-e) CD69 expression in primary human naive T cells and percentage of live T cells incubated on nanopores for 24 h under the influence of different GsMTx4 concentrations measured via flow cytometry. $n_D = 3$. c) Exemplary contour plot of CD69 expression. Pink box indicates the concentration used in f&g. d) Percentage of CD69⁺ T cells. e) Percentage of live T cells. f) Percentages of CD69⁺ primary human T cells stimulated for 24 h on the indicated substrates with and without 10 µM GsMTx4 measured via flow cytometry. Bars indicate the mean ± SD across donors ($n_D = 5$-9). g) Inhibitory effect of GsMTx4 on CD69 expression as shown in f displayed as percentage difference of CD69 expression. Points indicate the mean ± SD across donors ($n_D = 5$-9). a-g) Symbols indicate mean values of technical replicates for each donor. Statistical analysis was performed on per-donor mean values from donors present in both conditions using a paired t-test with Benjamini-Hochberg (FDR) adjustment for multiple comparisons. Significance symbols represent adjusted p-values: ns : $p \geq 0.05$, * : $p < 0.05$, ** : $p < 0.01$, *** : $p < 0.001$.

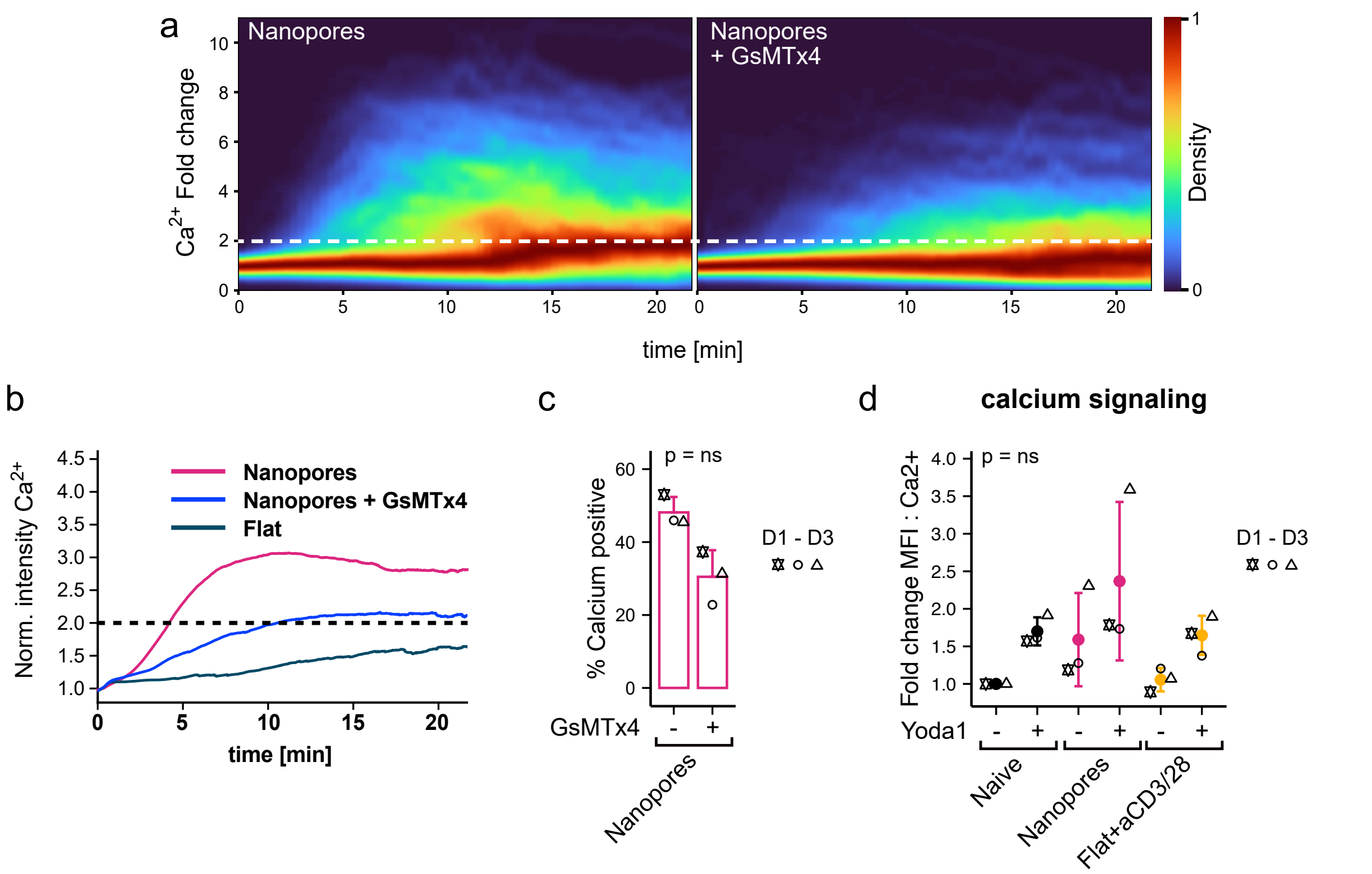

Supplementary Figure 10: GsMTx4 reduces nanopore-induced calcium signaling. a) Fluo4 intensity fold change of individual primary human T cells on nanopores with and without 10 µM GsMTx4 shown as a density plot from individual T cells (no GsMTx4 n = 1090, + GsMTx4 n = 739). b) Mean Fluo-4 intensity calculated from individual T cells from all donors over time showcasing the effect of GsMTx4 (10 µM) on calcium influx (n_D = 2 for Flat, n_D = 3 for Nanopores with and without GsMTx4). c) Percentage of T cells shown in a. obtaining a Fluo4 fold change of > 2 with and without GsMTx4. Bars indicate the mean ± SD across donors (n_D = 3). d) Fluo-8 median fluorescent intensity fold change relative to naive, as a calcium indicator after 10 min of incubation on different substrates with or without the addition of Yoda1 (20 µM), measured via flow cytometry. Points indicate the mean ± SD across donors (n_D = 3). c&d) Symbols indicate mean values of technical replicates for each donor. Statistical analysis was performed on per-donor mean values from donors present in both conditions using a paired t-test with Benjamini-Hochberg (FDR) adjustment for multiple comparisons. Significance symbols represent adjusted p-values: ns : p ≥ 0.05.

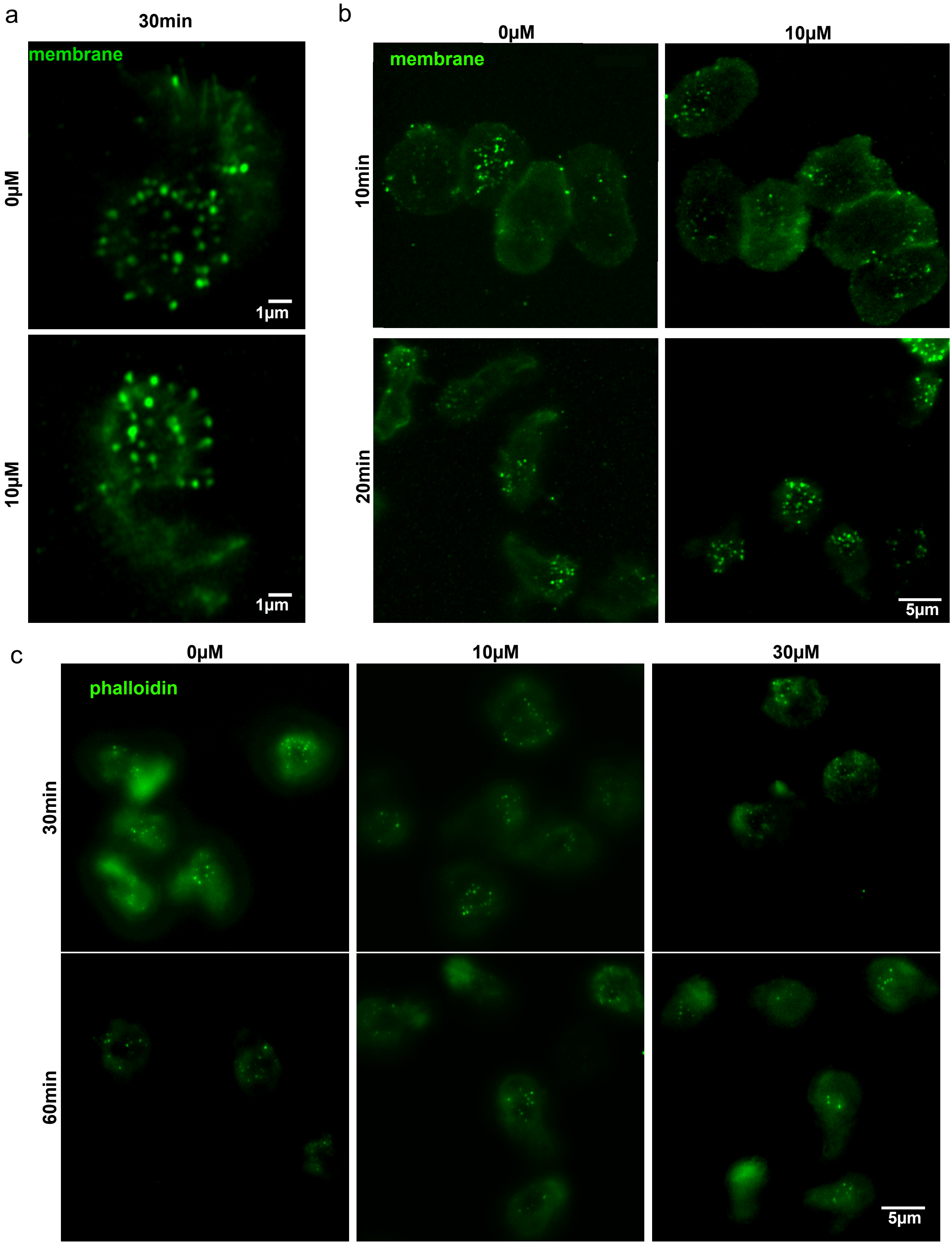

Supplementary Figure 11: Microvilli formation into nanopores under influence of GsMTx4. a-c) Airy scan images processed using Fiji. Z projection using "sum slices" of the membrane stain across the z slices within the nanopores. T cells were incubated for 10-60 min on the substrates with 0, 10 and 30µM GsMTx4 (pre-incubation with GsMTx4 for 30 min), respectively. a&b) The membrane was stained with WGA. c) Phalloidin was used to stain F-actin.

Biochemical induced signaling is more sensitive to PP2 than nanopore-induced signaling

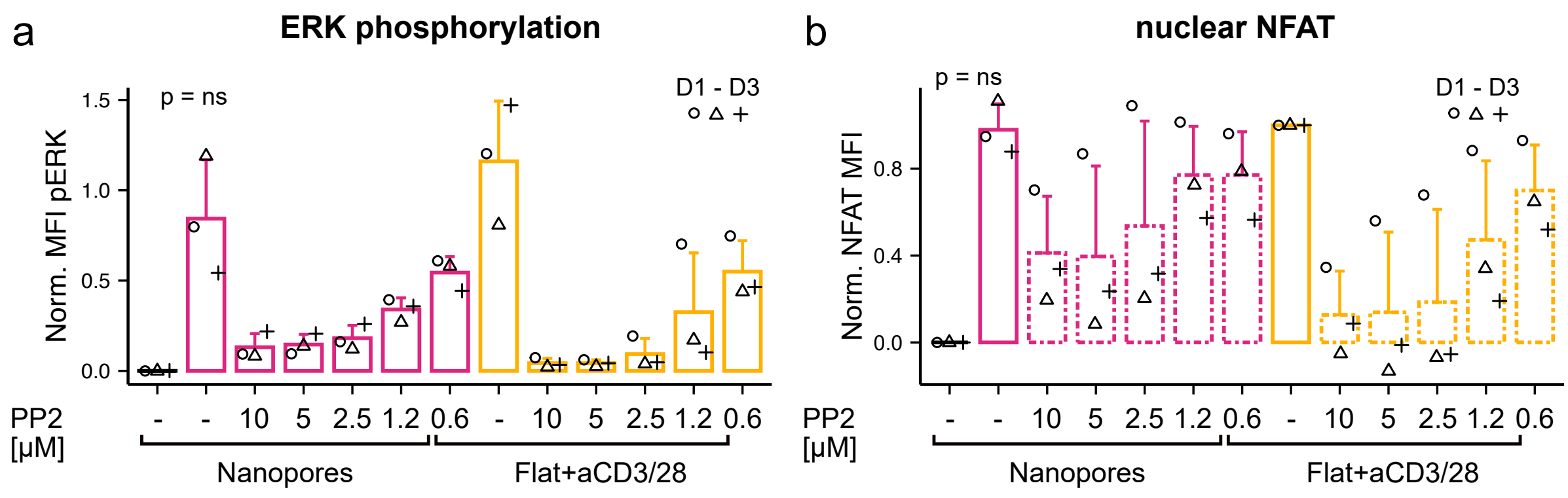

TCR knock down reduces early signaling

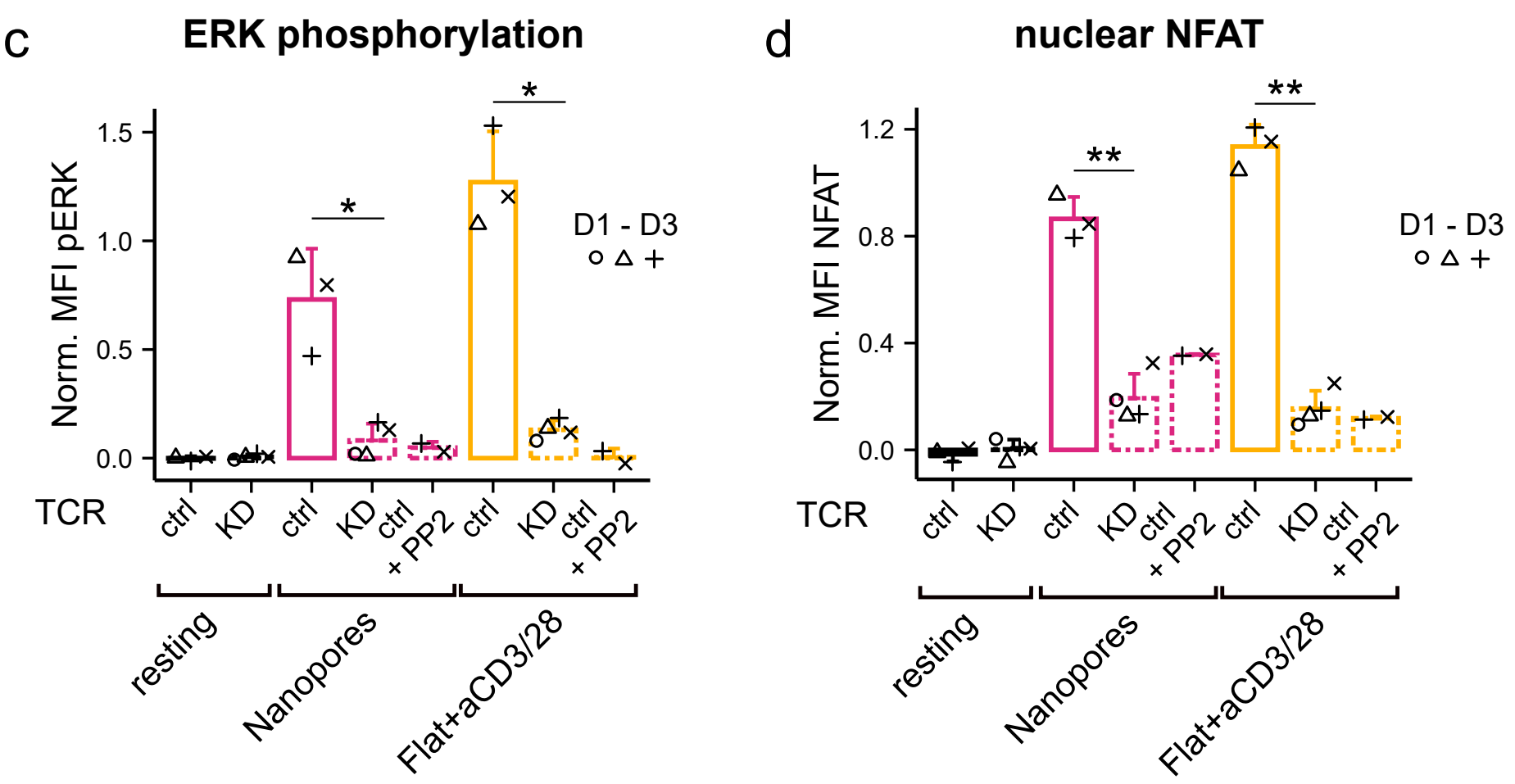

Supplementary Figure 12: Influence of pharmacological inhibition and TCR knockdown on early signaling. a&b) Naive primary human T cells were pre-incubated for 30 min with the indicated concentration of PP2 prior to incubation on the indicated substrates for 30 min. a) Median fluorescent intensity (MFI) of phosphorylated ERK (pERK) measured by flow cytometry, normalized per donor to the positive control samples (Flat+aCD3/28 and Nanopores without inhibitors) and naive as baseline. b) NFAT MFI in isolated nuclei measured by flow cytometry, normalized per donor to both positive control samples (Flat+aCD3/28) and naive as baseline. c&d) TCR knockdown T cells were incubated for 30 min on indicated substrates. c) MFI of pERK measured by flow cytometry, normalized per donor to the positive control samples (Flat + aCD3/28 and Nanopores) and naive as baseline. d) NFAT MFI in isolated nuclei measured by flow cytometry, normalized per donor to both positive control samples (Flat + aCD3/28) and naive as baseline. a-d) Bars indicate the mean ± SD across donors (D; a&b: n_D = 3; c&d: n_D = 2-3). Symbols indicate mean values of technical replicates for each donor. Statistical analysis was performed on per-donor mean values from donors present in both conditions using a paired t-test with Benjamini-Hochberg (FDR) adjustment for multiple comparisons. Significance symbols represent adjusted p-values: ns : $p \geq 0.05$, * : $p < 0.05$, ** : $p < 0.01$.

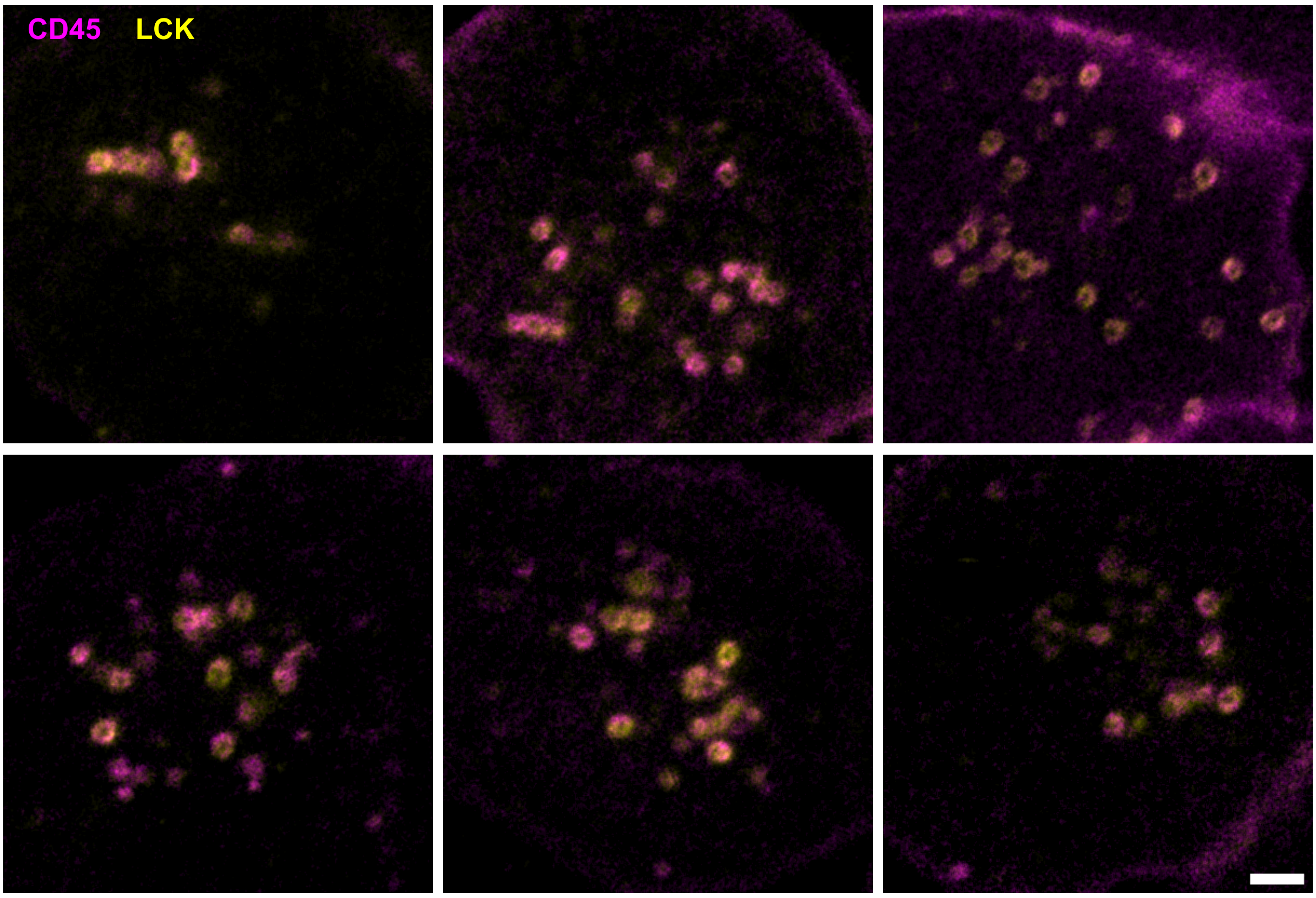

Supplementary Figure 13: CD45-low patches on microvilli imaged with STED. Composit STED images of individual T cells stained for CD45 (magenta) and LCK (yellow), acquired within nanopores, showing cross-sections of microvilli which appear as ring-like structures. Scale bar: 1 μm.

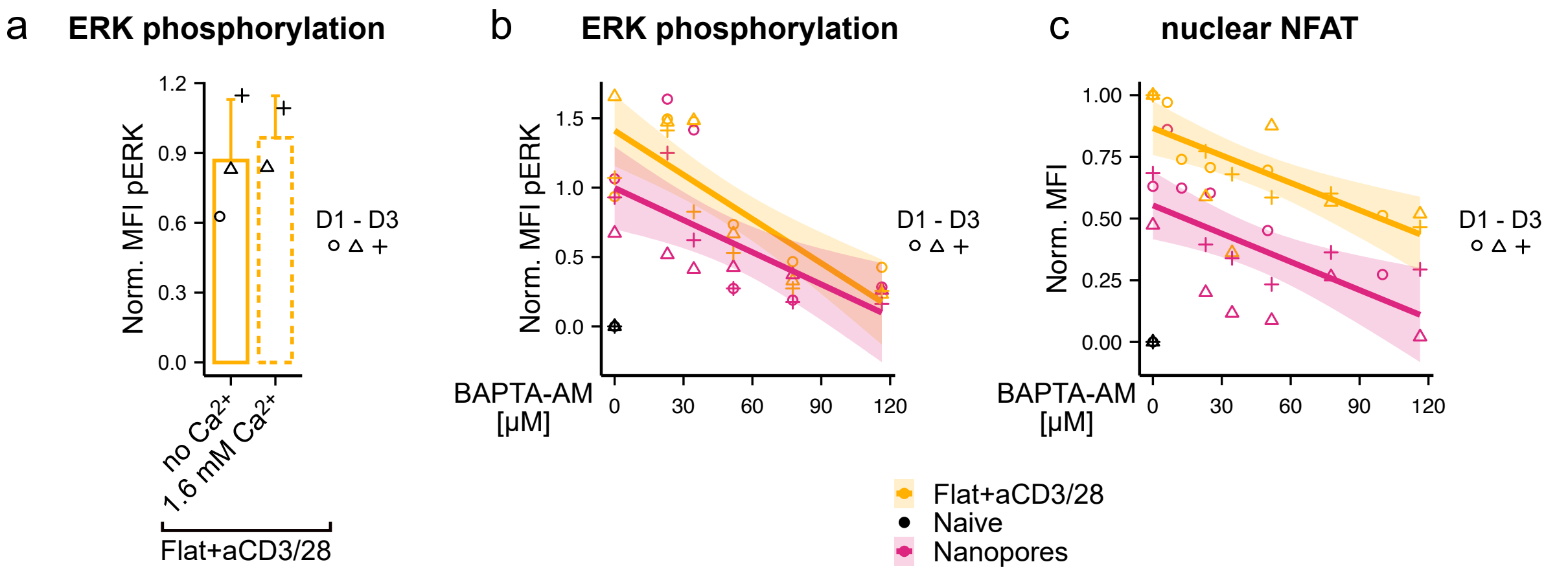

Supplementary Figure 14: Extracellular calcium supports ERK phosphorylation on nanopores. a)Primary human T cells were seeded and incubated for 30 min on Flat+aCD3/28 in HBSS medium containing the indicated concentration of calcium (Ca^{2+}). Median fluorescent intensity (MFI) of phosphorylated ERK measured by flow cytometry, normalized per donor to all positive control samples (Flat+aCD3/28 ± 1.6 mM Ca^{2+} and Nanopores + 1.6 mM Ca^{2+}) and naive as baseline. Bars indicate the mean ± SD across donors (D; n_D = 2-3). b&c) Naive primary human T cells were incubated with indicated concentration of BAPTA for 25 min. After medium replacement the cells were incubated for 30 min on incubated substrates. b) MFI of phosphorylated ERK measured by flow cytometry, normalized to Flat+aCD3/28 and naive as baseline. c) NFAT MFI in isolated nuclei measured by flow cytometry, normalized to Flat+aCD3/28 and naive as baseline. b&c) Linear regression (least squares) was performed for each sample. Shaded regions show the 95% confidence interval of the fit. a-c) Symbols indicate mean values of technical replicates for each donor.

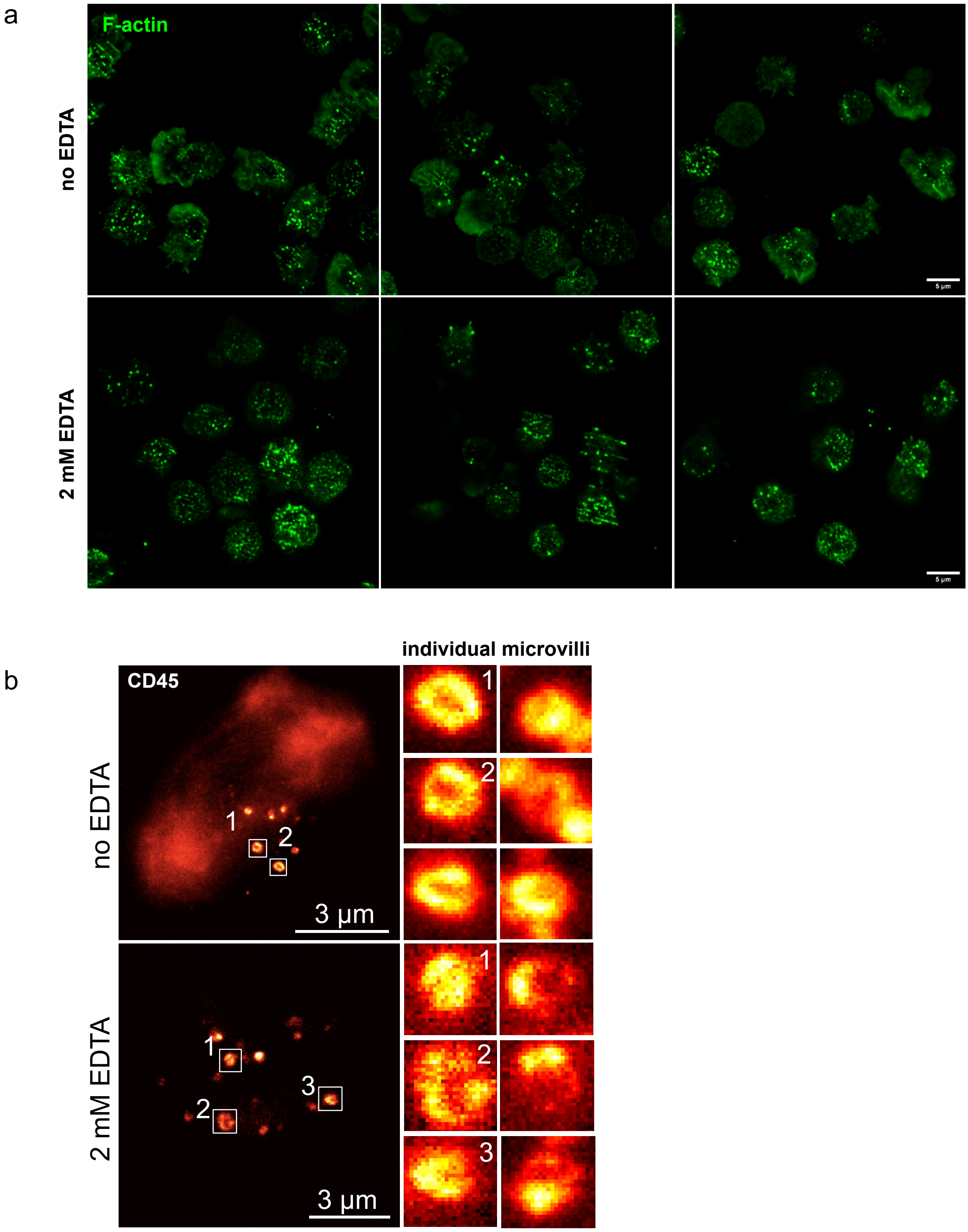

Supplementary Figure 15: Microvilli formation into nanopores under the influence of EDTA. a&b) T cells were incubated for 20 min with 0 or 2 mM EDTA on nanoporous substrates, respectively. a) Airy scan images of mutiple T cells on nanoporous substrates. Images were processed using Fiji. Z projection using "sum slices" of the membrane stain across the z slices within the nanopores are shown. Labelled phalloidin was used to stain F-actin. b) STED images of individual T cells stained for CD45, acquired within nanopores, showing cross-sections of microvilli which appear as ring-like structures (left). Individual microvilli cross-sections from different T cells (right). Scale image boxes = 500 nm.

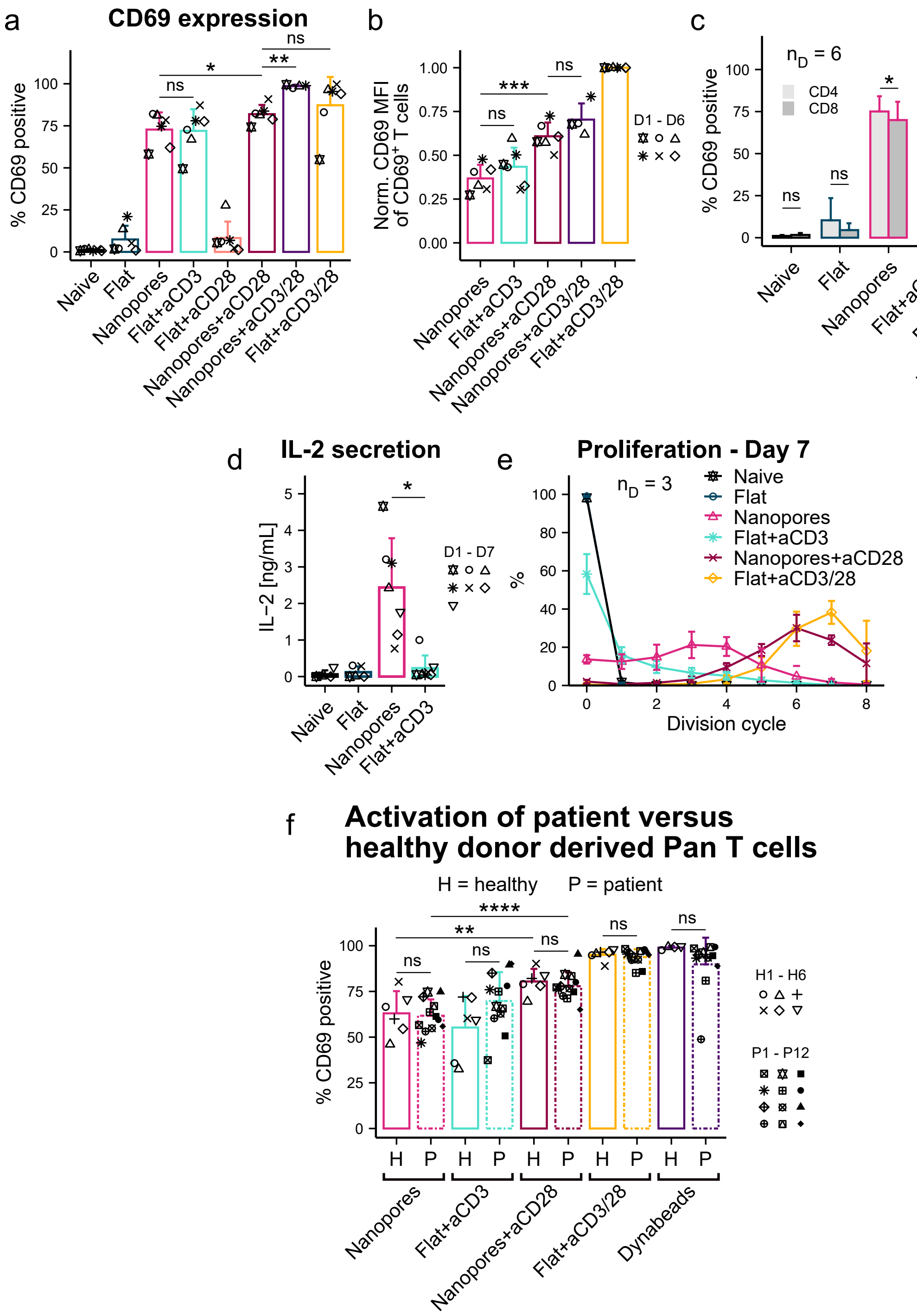

Supplementary Figure 16: Extent of activation of naive and Pan T cells. a-d) Naive primary human T cells were incubated 24 h on indicated substrates. a) Percentage of CD69+ T cells assessed by flow cytometry. b) CD69 MFI od CD69+ T cells normalized to Flat+aCD3/28 after background subtraction (Naive). c) CD69 positive T cells of CD4+ vs CD8+ T cells. d) IL-2 secretion assessed with a cytokine secretion assay. e) Naive primary human T cells loaded with Cell Trace Violet were stimulated for 72 hon indicated substrates and cell division cycle was assessed 7 days after seeding with flow cytometry and shown as mean percentage per division cycle for the different substrates. f) Pan T cells from healthy and DLBCL patient donors were incubated on the indicated substrates for 24 hours, shown as percentage of CD69+ T cells. a-d&f) Bars indicate the mean ± SD across healthy donors (D) or patients (P; a-c: n_D = 6; d:n_D = 5-7; f: n_D = 4-6, n_P = 11-12). Symbols indicate mean values of technical replicates for each healthy donor or patient. Statistical analyses within the patient and healthy donor groups were performed using paired t-tests on per-donor mean values from donors present in both conditions. Comparisons between patient and healthy donor groups were performed using unpaired t-tests. Multiple comparisons were corrected using the Benjamini–Hochberg false discovery rate (FDR) method. Significance symbols represent adjusted p-values: ns : $p \geq 0.05$, * : $p < 0.05$; ** : $p < 0.01$; *** : $p < 0.001$; **** : $p < 0.0001$.

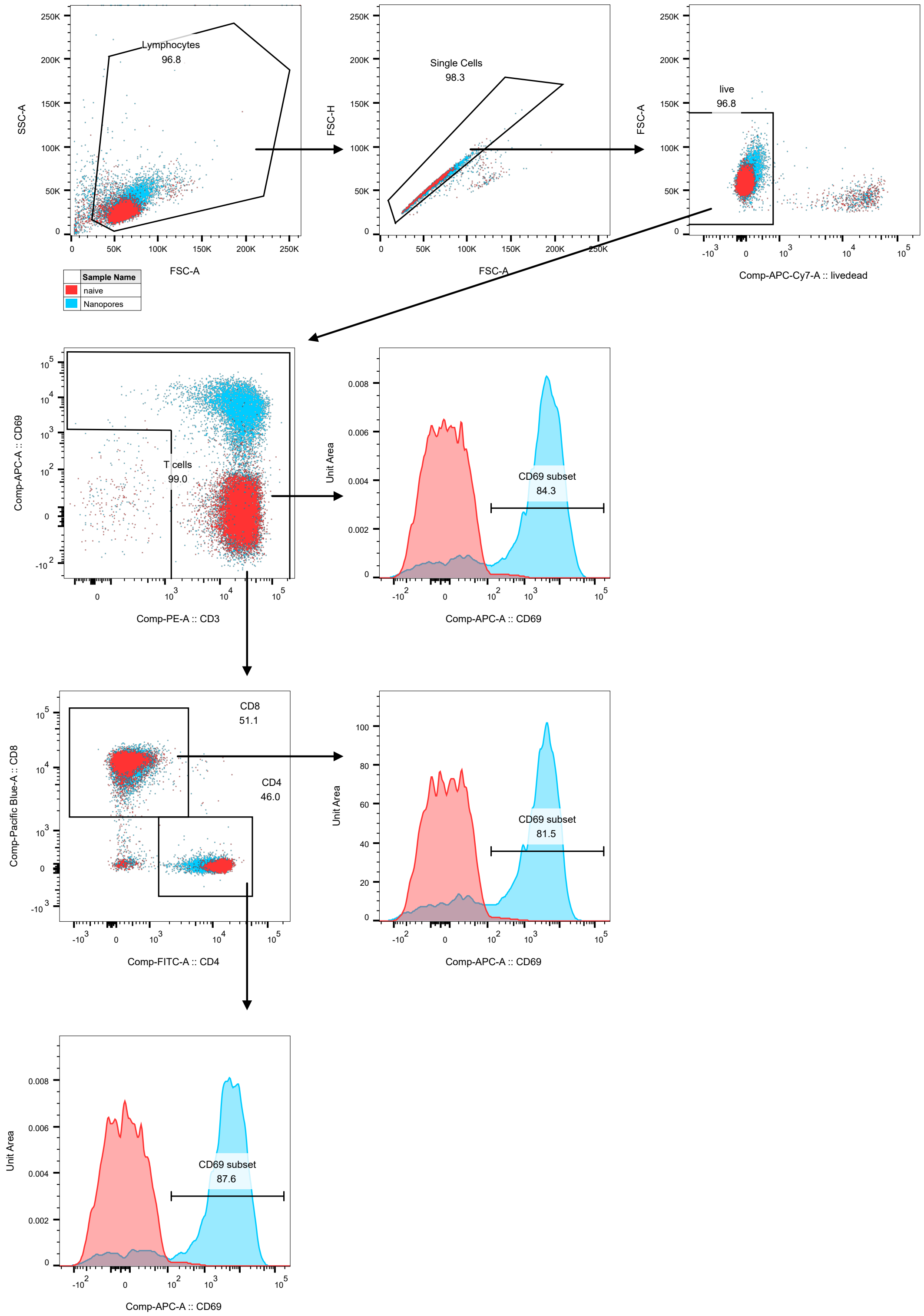

Supplementary Figure 17: Exemplary gating strategy for CD69 measurements.

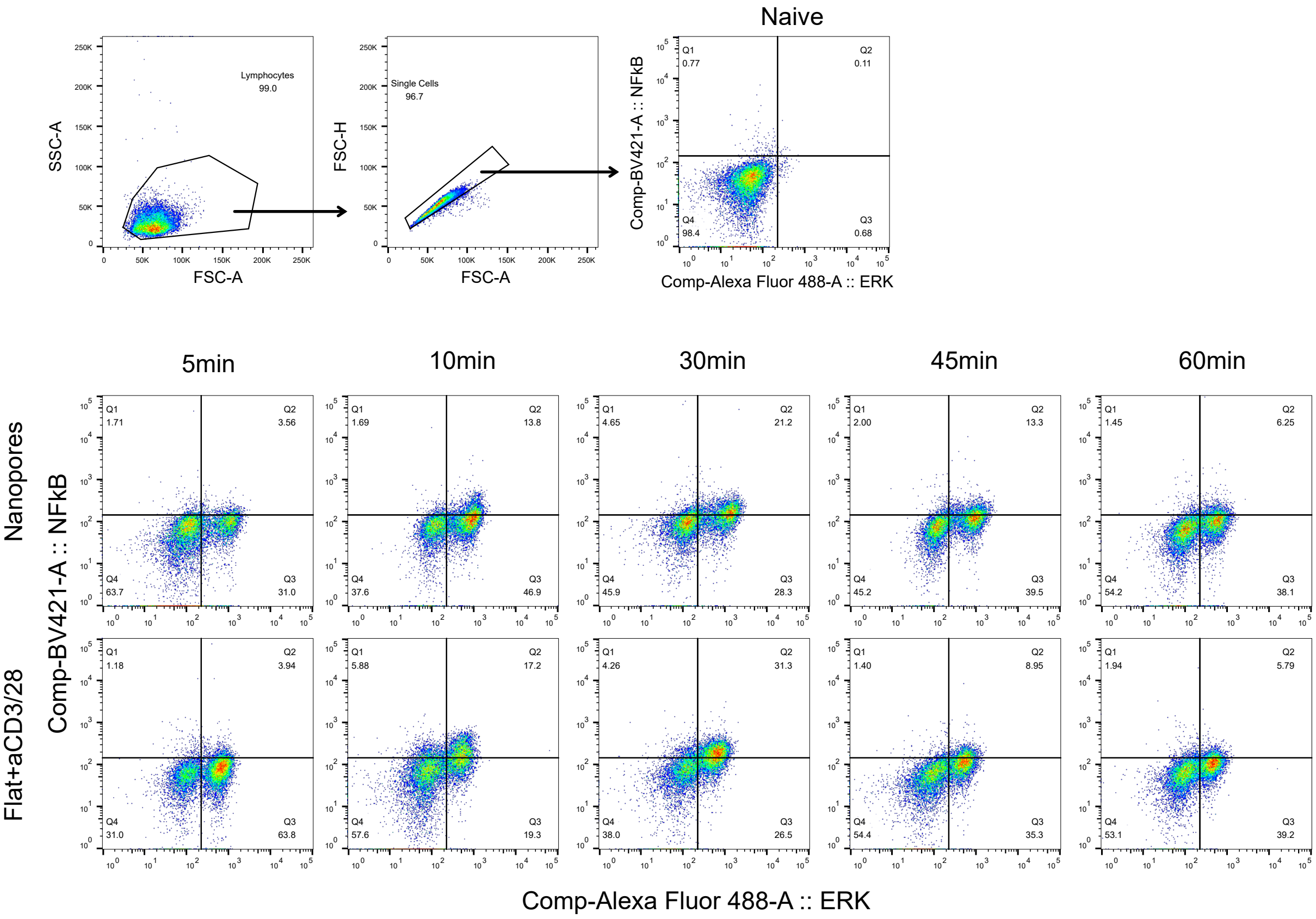

Supplementary Figure 18: ERK and pNFκB phospho-flow gating. Phosphoflow gating for pERK positive cells and MFI of pNFκB determination shown in Fig. 2 and Supplementary Fig. 4.

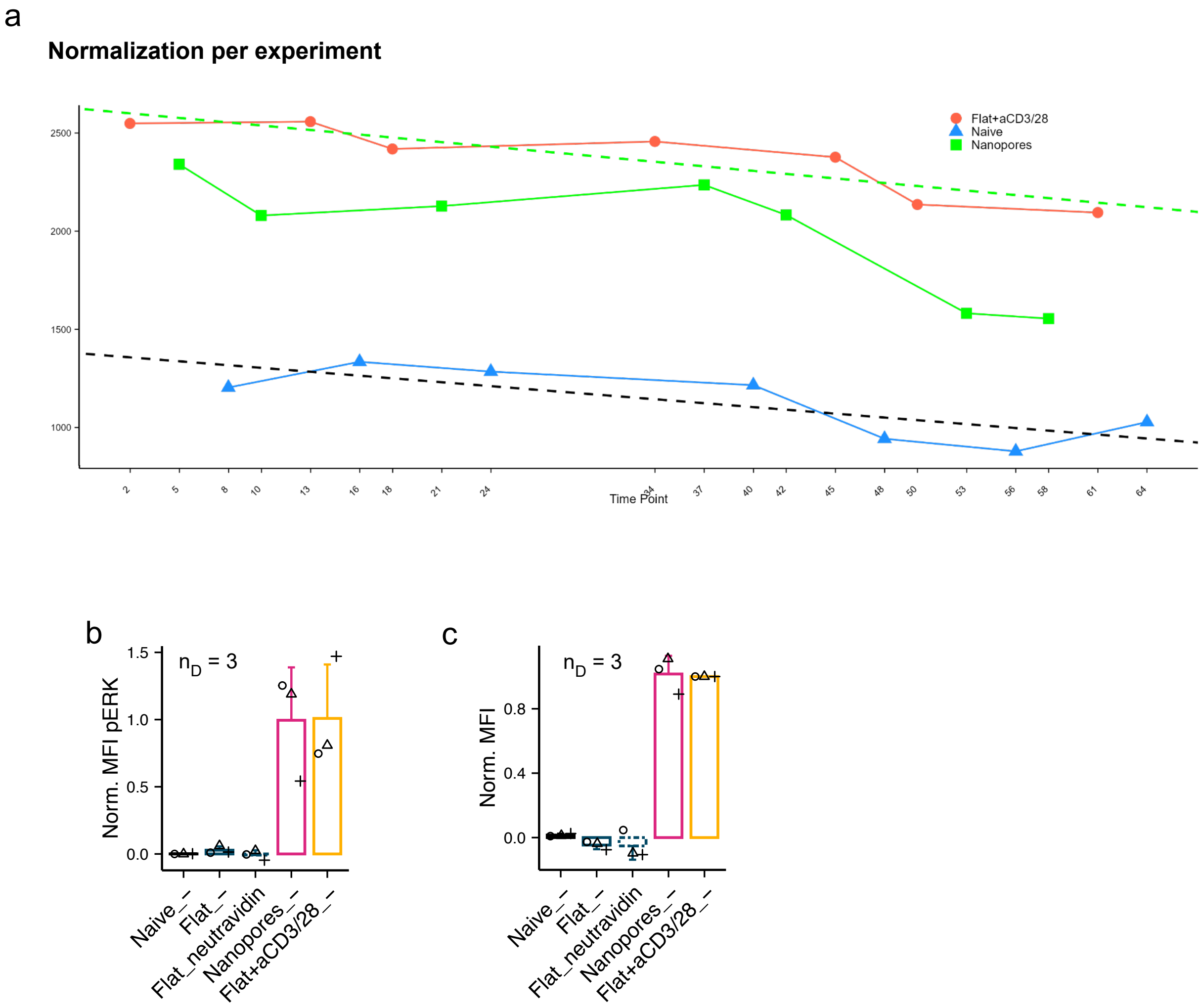

Supplementary Figure 19: pERK and NFAT signaling normalization and Flat controls. a) Per experiment, a regression for the baseline: naive and the positive samples was calculated. After baseline subtraction the values were normalized to the positive control samples (pERK: Flat+aCD3/28 and Nanopores; nuclear NFAT: Flat+aCD3/28). b&c) Flat controls: Norm. pERK MFI (b) and nuclear NFAT MFI (c) after 30 min of incubation on indicated substrates.

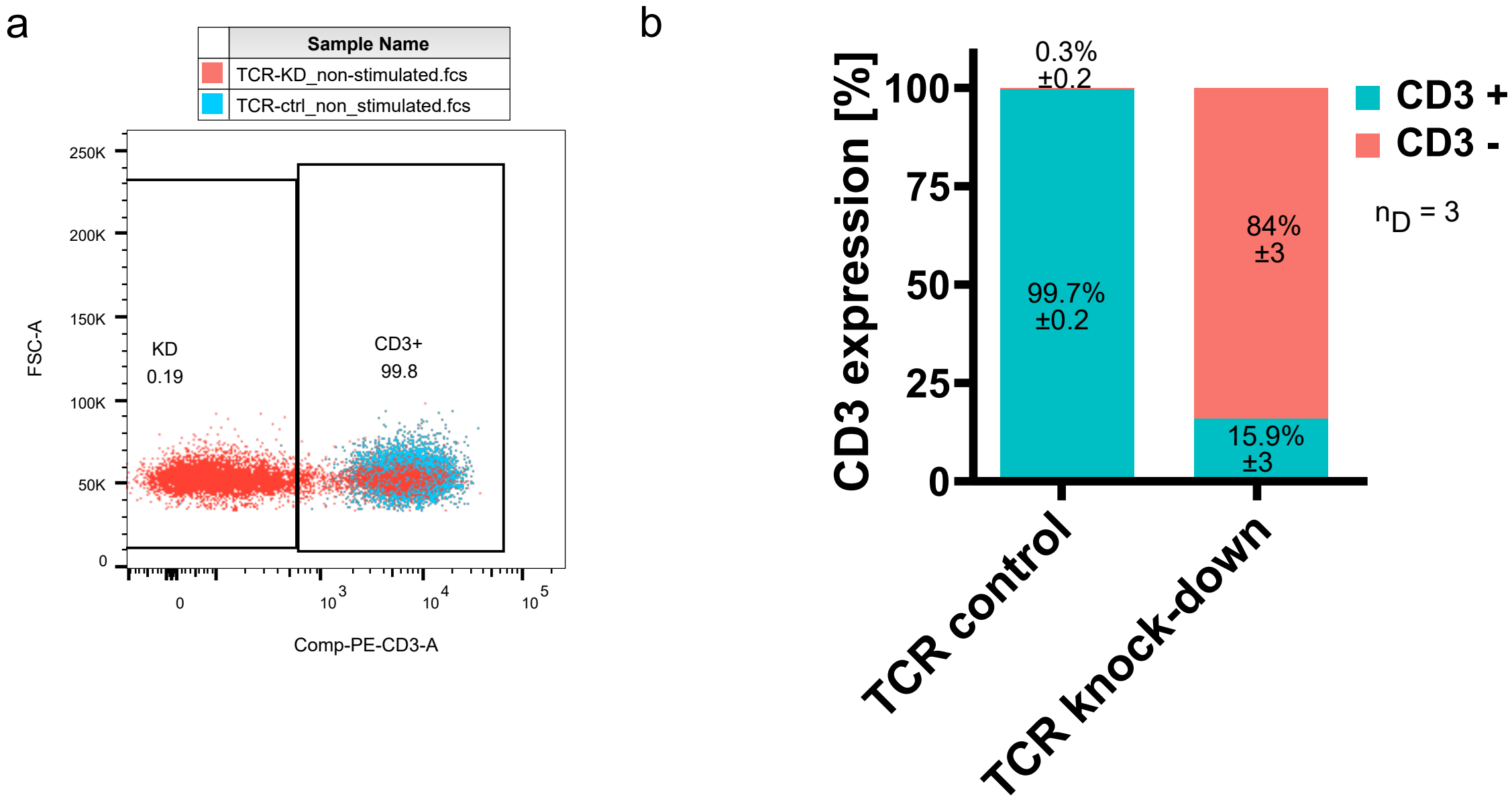

Supplementary Figure 20: TCR expression in primary human T cells after knockdown. A) Scatter plot displaying the CD3epsilon expression of primary human Pan T cells treated with (TCR-KD) and without (TCR-ctrl) RNPs against the TRAC locus several days (>3) after RNP electroporation. The gates were use for further analysis in B. B) Percentage of T cells from 3 donors expressing CD3epsilon on the cell surfaces.

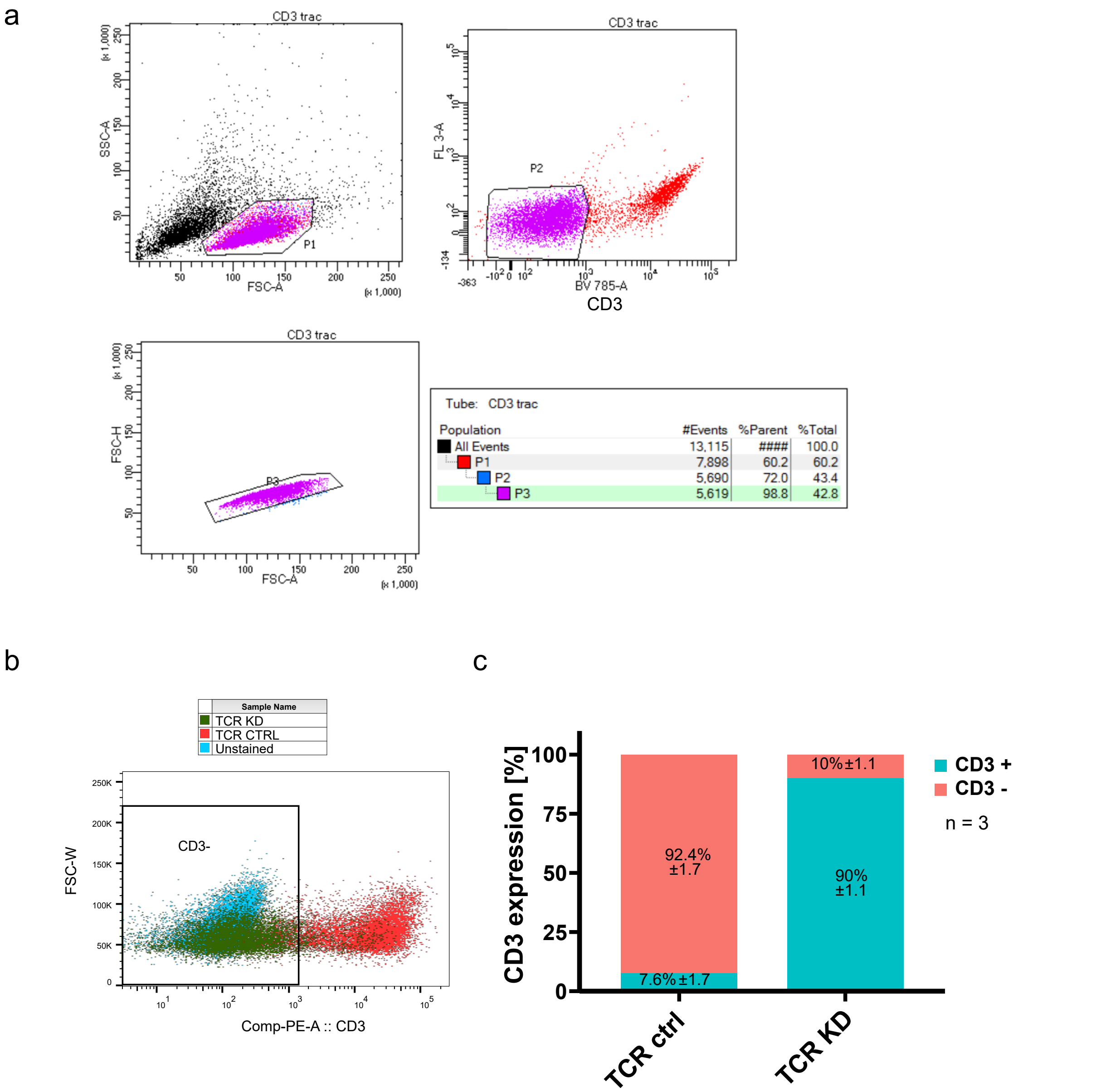

Supplementary Figure 21: TCR KD Jurkat T cell generation. A) Jurkat T-cells were sorted for gate P3 at the Flow Cytometry Core Facility. B) Scatter plot of CD3 expression more than 4 passages passages after sorting. C) Analysis of CD3 expression of wt and CD3- sorted Jurkat T-cells.

a

b

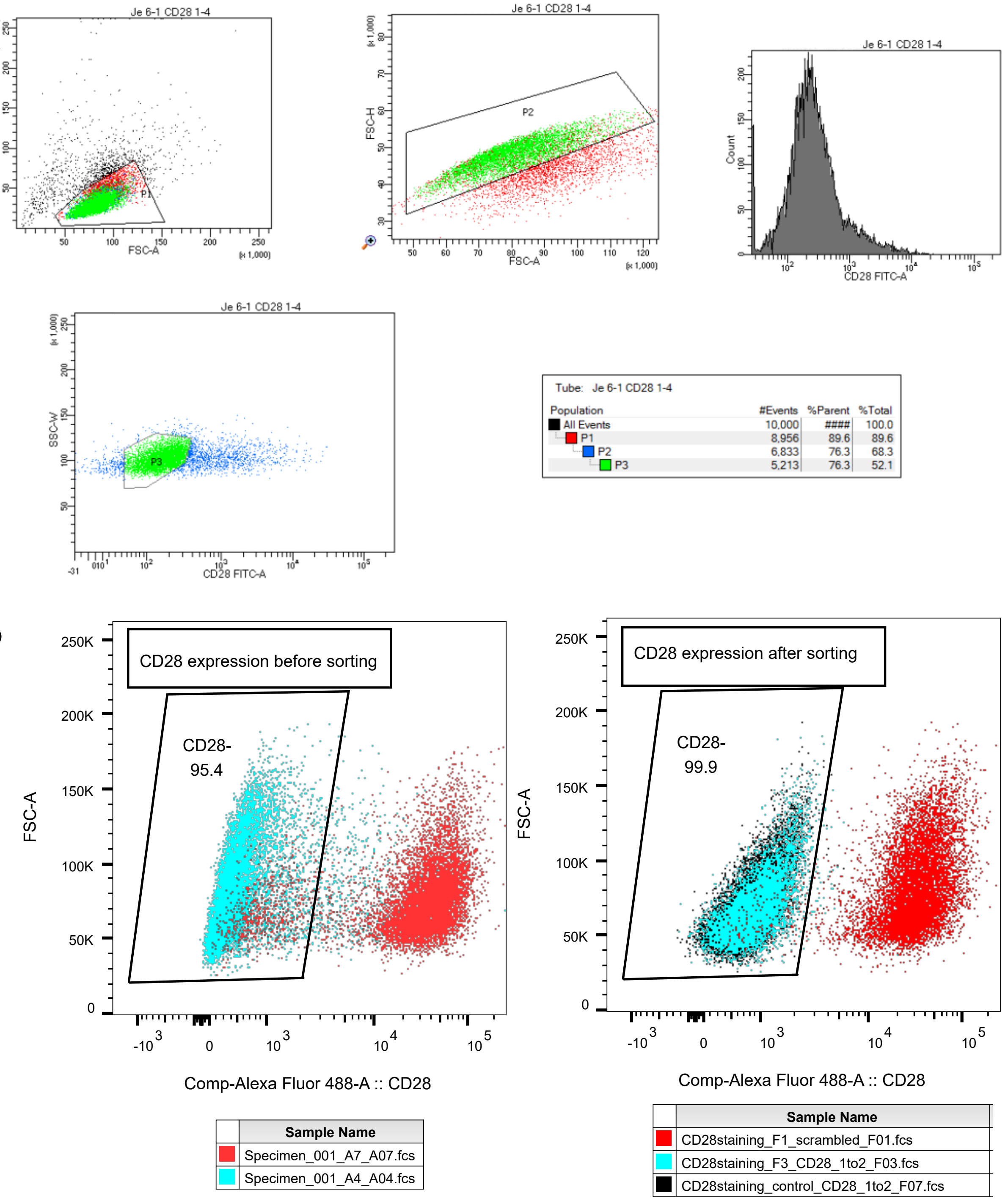

Supplementary Figure 22: CD28 KD Jurkat T cells generation. A) Jurkat T-cells were sorted for gate P3 at the Flow Cytometry Core Facility. B) Scatter plot of CD28 expression before and after sorting (> 4 passages).
